# Supplementary material for: Boron-chalcogen heterocycles and linear tetraboranes from a cyclic tetra(amino)tetraborane
Source: Nat Commun. 2025 Jun 12;16:5304. doi: 10.1038/s41467-025-60549-z (PMC12163077; doi:10.1038/s41467-025-60549-z)
Supplement: Supplementary file 1 — Supplementary Information [file 41467_2025_60549_MOESM1_ESM.pdf]

Supplementary Information for

Boron-Chalcogen Heterocycles and Linear Tetraboranes from a Cyclic Tetra(amino)tetraborane

Eva Beck[1,2], Diana Bröllos[1,2], Ivo Krummenacher[1,2], Thomas Kupfer[1,2], Maximilian Dietz[1,2], Tim Wellnitz[1,2], Cornelius Mihm[1,2] & Holger Braunschweig[1,2 *]

[1]Institut für Anorganische Chemie, Julius-Maximilians-Universität Würzburg, Am Hubland, 97074 Würzburg, Germany. [2]Institute for Sustainable Chemistry & Catalysis with Boron, Julius-Maximilians-Universität Würzburg, Am Hubland, 97074 Würzburg, Germany.

Index

SUPPLEMENTARY METHODS S2

GENERAL PROCEDURES S2

EXPERIMENTAL PROCEDURES S3

NMR SPECTRA S10

CYCLIC VOLTAMMETRY S50

UV-VIS SPECTRUM S52

EPR SPECTRUM S53

SINGLE-CRYSTAL X-RAY DIFFRACTION DATA S54

COMPUTATIONAL DETAILS S64

SUPPLEMENTARY REFERENCES S66

Supplementary Methods

General Procedures

All manipulations were performed either under an atmosphere of dry argon or *in vacuo* using standard Schlenk line or glovebox techniques. Deuterated solvents were dried over molecular sieves and degassed by three freeze-pump-thaw cycles prior to use. All other solvents were distilled and degassed from appropriate drying agents. Both deuterated and non-deuterated solvents were stored under argon over activated 4 Å molecular sieves. Liquid-phase NMR spectra were acquired on a Bruker Avance 400 (^{1}H: 400.1 MHz, ^{11}B:128.5 MHz, ^{13}C: 100.7 MHz), Bruker Avance 500 (^{11}B: 160.5 MHz, ^{13}C: 125.7 MHz), or Bruker Avance 600 (^{1}H: 600.1 MHz, ^{11}B: 192.6 MHz, ^{13}C: 150.9 MHz, ^{19}F: 564.7 MHz, ^{77}Se: 114.5 MHz, ^{125}Te: 189.3 MHz) spectrometers. Chemical shifts (δ) are reported in ppm and internally referenced to the carbon nuclei ($^{13}C\{^{1}H\}$) or residual protons (^{1}H) of the solvent. Heteronuclei NMR spectra are referenced to external standards (^{11}B: $BF_3{\cdot}OEt_2$, ^{19}F: CCl_3F, ^{77}Se: Me_2Se, ^{125}Te: Me_2Te). Resonances are given as singlet (s), multiplet (m) or broad (br). High-resolution mass spectrometry (HRMS) data were obtained from a Thermo Scientific Exactive Plus spectrometer. EPR measurements at X-band (9.86 GHz) were carried out using a Bruker ELEXSYS E580 CW EPR spectrometer. The spectral simulations were performed using MATLAB 9.12.0.1884302 (R2022a) and the EasySpin 5.2.33 toolbox[1]. Cyclic voltammetry experiments were performed using a Gamry Instruments Reference 600 potentiostat. A standard three-electrode cell configuration was employed using a platinum disk working electrode, a platinum wire counter electrode, and a silver wire, separated by a Vycor tip, serving as the reference electrode. Formal redox potentials are referenced to the ferrocene/ferrocenium ($[Cp_2Fe]^{+/0}$) redox couple by using decamethylferrocene ($[Cp^*_2Fe]$; $E_{1/2}$ = −0.427 V in THF) as an internal standard. Tetra(*n*-butyl)ammonium hexafluorophosphate ($[nBu_4N][PF_6]$) was employed as the supporting electrolyte. Compensation for resistive losses (iR drop) was employed for all measurements. Photolysis reactions were carried out on an Hg/Xe arc lamp (I = 19 A, U = 26 V, P = 500 W, λ = 210–600 nm) from the company LOT-QuantumDesign. Solvents, hexamethyldisilazane (HMDS), Ph_2S_2, Ph_2Se_2, Ph_2Te_2, SbF_3, C_2Cl_6, Br_2, and AgOTf (OTf = trifluoromethanesulfonate) were purchased from Sigma-Aldrich, ABCR or Alfa Aesar. $[Ag][Al\{OC(CF_3)_3\}_4]$ was provided by the group of Prof. Ingo Krossing (University of Freiburg, Germany).

Experimental procedures

Synthesis of $B_4(NCy_2)_4$ (1). $B_4(NCy_2)_4$ (**1**) was prepared by the following procedure[2]: $(Cy_2N)BCl_2$ (200 mg, 760 µmol) and sodium sand (69.9 mg, 3.04 mmol, 4.0 equiv.) were combined in pentane (10 mL) and stirred for 3 d at room temperature, during which time a purple color gradually developed, accompanied by the precipitation of a purple solid. After filtration, all volatile components were removed in vacuo, and the resulting residue was washed with dme (3 × 5 mL) and dried under reduced pressure. Thus, **1** was isolated as a blue solid (60.0 mg, 78.4 µmol, 41%).

$^1H\{^{11}B\}$ NMR (400.1 MHz, C_6D_6, 297 K): δ = 3.39–3.27 (m, 8H, C*H*), 2.04–1.94 (m, 16H, *o*-CH_2), 1.88–1.80 (m, 16H, *m*-CH_2), 1.71–1.58 (m, 24H, *o*-/*p*-CH_2), 1.46–1.32 (m, 16H, *m*-CH_2), 1.25–1.12 (m, 8H, *p*-CH_2) ppm. **$^{13}C\{^1H\}$ NMR** (100.7 MHz, C_6D_6, 297 K): δ = 63.9 (*C*H), 35.7 (*o*-*C*H$_2$), 26.9 (*m*-*C*H$_2$), 26.2 (*p*-*C*H$_2$) ppm. **^{11}B NMR** (128.5 MHz, C_6D_6, 297 K): δ = 68.0 (br s) ppm. **HRMS LIFDI** for $[C_{48}H_{88}N_4B_4]^+$ = $[M]^+$: calc. 764.7376, found 764.7372. Analytical data are in agreement with literature values[2].

Synthesis of $B_4(NCy_2)_4S$ (2S). $B_4(NCy_2)_4$ (**1**, 100 mg, 131 µmol) and diphenyl disulfide (14.3 mg, 65.4 µmol, 0.5 equiv.) were combined in benzene (6 mL) and stirred for 2 d under UV irradiation, resulting in the decolorization of the blue solution. After removing all volatiles from the reaction mixture under vacuum, the residue was washed with DME (4 × 5 mL), filtered, and then dried under reduced pressure. This yielded product **2S** as a colorless solid (85.0 mg, 106 µmol, 81%). Single crystals suitable for X-ray diffraction analysis were obtained by recrystallization from hexane at –30 °C.

Note: When $B_4(NCy_2)_4$ (**1**) is reacted with diphenyl sulfide, **2S** is obtained in 74% yield.

$^1H\{^{11}B\}$ NMR (600.1 MHz, C_6D_6, 297 K): δ = 3.39–3.30 (m, 4H, C*H*), 3.26–3.14 (m, 2H, CH_2), 3.07–3.00 (m, 2H, C*H*), 2.97–2.76 (m, 2H, C*H*), 2.02–1.63 (m, 40H, CH_2), 1.61–1.47 (m, 12H, CH_2), 1.44–1.18 (m, 22H, CH_2), 1.13–1.00 (m, 4H, CH_2) ppm. **$^{13}C\{^1H\}$ NMR** (125.7 MHz, C_6D_6, 297 K): δ = 66.3 (*C*H), 66.1 (*C*H), 60.5 (*C*H), 57.0 (*C*H), 36.5 (*C*H$_2$), 36.0 (*C*H$_2$), 34.8 (*C*H$_2$), 34.0 (*C*H$_2$), 33.8 (*C*H$_2$), 33.5 (*C*H$_2$), 33.3 (*C*H$_2$), 32.7 (*C*H$_2$), 27.6 (*C*H$_2$), 27.5 (*C*H$_2$), 27.2 (*C*H$_2$), 27.1 (*C*H$_2$), 27.1 (*C*H$_2$), 26.6 (*C*H$_2$), 26.5 (*C*H$_2$), 26.3 (*C*H$_2$), 26.3 (*C*H$_2$), 26.2 (*C*H$_2$), 26.0 (*C*H$_2$) ppm. **^{11}B NMR** (160.5 MHz, C_6D_6, 297 K): δ = 55.5 (br s), 45.9 (br s) ppm. **HRMS LIFDI** for $[C_{48}H_{88}B_4\ N_4S]^+$ = $[M]^+$: calc. 796.7092; found 796.7096.

Synthesis of $B_4(NCy_2)_4Se$ (2Se). $B_4(NCy_2)_4$ (**1**, 100 mg, 131 µmol) and diphenyl diselenide (20.4 mg, 65.4 µmol, 0.5 equiv.) were combined in benzene (5 mL) and stirred for 2 d at 80 °C, resulting in a color change of the reaction mixture from blue to yellow. After removing all volatiles from the reaction mixture under vacuum, the residue was washed with DME (4 × 5 mL), filtered, and dried under reduced pressure. This yielded product **2Se** as a colorless solid (66.3 mg, 78.6 µmol, 60%). Single crystals suitable for X-ray diffraction analysis were obtained by recrystallization from hexane at –30 °C.

$^1H\{^{11}B\}$ NMR (600.1 MHz, C_6D_6, 297 K): δ = 3.40–3.32 (m, 4H, C*H*), 3.08–3.00 (m, 2H, C*H*), 2.99–2.91 (m, 1H, C*H*), 2.88–2.78 (m, 2H, C*H*), 2.10–2.04 (m, 4H, C*H*$_2$), 1.99–1.63 (m, 38H, C*H*$_2$), 1.61–1.46 (m, 12H, C*H*$_2$), 1.43–1.17 (m, 22H, C*H*$_2$), 1.13–0.98 (m, 4H, C*H*$_2$) ppm. **$^{13}C\{^1H\}$ NMR** (150.9 MHz, C_6D_6, 297 K): δ = 67.3 (*C*H), 66.6 (*C*H), 60.8 (*C*H), 57.5 (*C*H), 36.7 (*C*H$_2$), 36.3 (*C*H$_2$), 35.0 (*C*H$_2$), 33.8 (*C*H$_2$), 33.2 (*C*H$_2$), 32.6 (*C*H$_2$), 27.5 (*C*H$_2$), 27.4 (*C*H$_2$), 27.3 (*C*H$_2$), 27.2 (*C*H$_2$), 27.2 (*C*H$_2$), 27.1 (*C*H$_2$), 26.6 (*C*H$_2$), 26.5 (*C*H$_2$), 26.3 (*C*H$_2$), 26.3 (*C*H$_2$), 26.2 (*C*H$_2$), 25.9 (*C*H$_2$) ppm. **^{11}B NMR** (192.6 MHz, C_6D_6, 297 K): δ = 55.4 (br s), 46.8 (br s) ppm. **$^{77}Se\{^{11}B,^1H\}$ NMR** (114.5 MHz, C_6D_6, 297 K): δ = 254 ppm. **HRMS LIFDI** for $[C_{48}H_{88}B_4N_4Se]^+$ = $[M]^+$: calc. 843.6569; found 843.6577.

Synthesis of $B_4(NCy_2)_4Te$ (2Te). $B_4(NCy_2)_4$ (**1**, 60.0 mg, 78.5 µmol) and diphenyl ditelluride (16.1 mg, 39.2 µmol, 0.5 equiv.) were combined in benzene (5 mL) and stirred for 1 d under UV irradiation, resulting in a color change of the reaction mixture from blue to brown. After removal of all volatiles from the reaction mixture in vacuo, the residue was washed with DME (3 × 5 mL), filtered, and dried under reduced pressure. This yielded product **2Te** as a colorless solid (34.6 mg, 38.8 µmol, 49%). Single crystals suitable for X-ray diffraction analysis were obtained by recrystallization from THF at –30 °C.

$^1H\{^{11}B\}$ NMR (600.1 MHz, C_6D_6, 297 K): δ = 3.56–3.35 (m, 6H, C*H and* C*H*$_2$), 3.11–2.95 (m, 4H, C*H*), 2.86–2.75 (m, 2H, C*H*$_2$), 2.28–2.20 (m, 2H, C*H*$_2$), 2.01–1.46 (m, 52H, C*H*$_2$), 1.40–1.15 (m, 18H, C*H*$_2$), 1.12–0.98 (m, 4H, C*H*$_2$) ppm. **$^{13}C\{^1H\}$ NMR** (150.9 MHz, C_6D_6, 297 K): δ = 68.7 (*C*H), 66.9 (*C*H), 61.1 (*C*H), 57.6 (*C*H), 36.5 (*C*H$_2$), 36.5 (*C*H$_2$), 35.0 (*C*H$_2$), 33.6 (*C*H$_2$), 33.2 (*C*H$_2$), 32.8 (*C*H$_2$), 32.7 (*C*H$_2$), 32.6 (*C*H$_2$), 27.1 (*C*H$_2$), 27.0 (*C*H$_2$), 27.0 (*C*H$_2$), 26.9 (*C*H$_2$), 26.2 (*C*H$_2$), 26.0 (*C*H$_2$), 25.9 (*C*H$_2$) ppm. **^{11}B NMR** (192.6 MHz, C_6D_6, 297 K): δ = 58.7 (br s), 44.5 (br s) ppm. **^{125}Te NMR** (189.3 MHz, C_6D_6, 297 K): δ = 15.6 ppm. **HRMS LIFDI** for $[C_{48}H_{88}B_4N_4Te]^+$ = $[M]^+$: calc. 893.6443; found 893.6481.

Synthesis of $B_2(NCy_2)_2S_2$ (3S). $B_4(NCy_2)_4$ (30.0 mg, 39.2 µmol) and S_8 (5.03 mg, 19.6 µmol, 0.5 equiv.) were combined in benzene (3 mL) and stirred for 5 d at 85 °C, resulting in a color change of the reaction mixture from blue to yellow. After removing all volatiles from the reaction mixture under vacuum, the residue was washed with pentane (2 × 2 mL), filtered, and dried under reduced pressure. This yielded **3S** as a colorless solid (5.30 mg, 11.9 µmol, 15%). Single crystals suitable for X-ray diffraction analysis were obtained by recrystallization from benzene at room temperature.

$^1H\{^{11}B\}$ NMR (400.1 MHz, C_6D_6, 297 K): δ = 3.39–3.23 (m, 4H, C*H*), 1.95–1.70 (m, 16H, *o*-C*H₂*), 1.68–1.59 (m, 8H, m-C*H or p*-CH₂), 1.51–1.40 (m, 4H, m-C*H or p*-CH₂), 1.24–1.10 (m, 8H, *m*-C*H or p*-CH₂), 1.05–0.91 (m, 4H, *m*-C*H or p*-CH₂ ppm. **$^{13}C\{^1H\}$ NMR** (100.7 MHz, C_6D_6, 297 K): δ = 58.2 (*C*H), 34.0 (*o*-*C*H₂), 26.7 (*C*H₂), 25.8 (*C*H₂) ppm. **^{11}B NMR** (128.5 MHz, C_6D_6, 297 K): δ = 37.5 (br s) ppm. **HRMS LIFDI** for $[C_{24}H_{44}B_2N_2S_2]^+$ = $[M]^+$; *m/z*: calc. 446.3127; found 446.3119.

Synthesis of $[B_4(NCy_2)_4S][Al\{OC(CF_3)_3\}_4]$ ($[\mathbf{2S}][Al\{OC(CF_3)_3\}_4]$). To a solution of **2S** (100 mg, 125 µmol) in 1,2-difluorobenzene (8 mL), $[Ag][Al(OC(CF_3)_3)_4]$ (134 mg, 125 µmol, 1.0 equiv.) was added and stirred for 20 min at room temperature under exclusion of light, resulting in the reaction mixture turning dark green. The insoluble materials in the reaction mixture were subsequently removed by filtration, and the solvent from the green filtrate was evaporated under reduced pressure. The residue was washed with benzene (2 × 5 mL) and dried under reduced pressure, yielding [**2S**]$[Al\{OC(CF_3)_3\}_4]$ as a dark green solid (175 mg, 99.2 µmol, 79%). Single crystals suitable for X-ray diffraction analysis were obtained by crystallization in a mixture of toluene and DME at –30 °C.

HRMS LIFDI for $[C_{48}H_{88}B_4N_4S_1]^+$ = $[M]^+$: calc. 796.7096; found 796.7089. **UV-vis** (1,2-difluorobenzene): λ_1 = 422 nm, λ_2 = 673 nm (shoulder).

Synthesis of $B_4(NCy_2)_4S(OTf)_2$ (4S). $B_4(NCy_2)_4S$ (**2S**, 50 mg, 62.8 µmol) and AgOTf (32.3 mg, 125.5 µmol, 2.0 equiv.) were combined in benzene (4 mL) and stirred for 17 h at 50 °C, causing the reaction mixture to change from colorless to yellow. The insoluble materials in the reaction mixture were separated by filtration. After the removal of all volatiles from the reaction mixture in vacuo, the residue was extracted with cold pentane (4 × 1 mL, –30 °C), filtered again, and the solvent from the filtrate was removed under reduced pressure. The extraction in cold pentane was repeated until the filtrate became colorless, yielding **4S** as a colorless solid (23.0

mg, 21.0 μmol, 33%). Single crystals suitable for X-ray diffraction analysis were obtained by crystallization in toluene at –30 °C.

Note: In solution, a mixture of temperature-dependent conformational isomers is present, which cannot be distinguished from each other.

$^{1}H\{^{11}B\}$ NMR (600.1 MHz, *d*$_8$-toluene, 298.15 K): δ = 4.17–4.02 (m, 1H, C*H*), 3.52–3.39 (m, 2H, C*H*), 3.23–3.07 (m, 2H, C*H*), 2.82–2.60 (m, 2H, C*H*), 2.45–2.33 (m, 1H, C*H*), 2.06–0.82 (m, 80H, C*H*$_2$) ppm. **$^{13}C\{^{1}H\}$ NMR** (150.9 MHz, *d*$_8$-toluene, 293.15 K): δ = 131.4 (*C*F$_3$), 122.4 (*C*F$_3$), 120.3 (*C*F$_3$), 118.2 (*C*F$_3$), 116.1 (*C*F$_3$), 71.1 (*C*H), 68.4 (*C*H), 66.3 (*C*H), 61.0 (*C*H), 60.8 (*C*H), 60.4 (*C*H), 59.8 (*C*H), 59.4 (*C*H), 57.0 (*C*H), 56.0 (*C*H), 56.0 (*C*H), 55.8 (*C*H), 55.2 (*C*H), 54.3 (*C*H), 54.1 (*C*H), 38.2 (*C*H$_2$), 37.8 (*C*H$_2$), 37.1 (*C*H$_2$), 36.6 (*C*H$_2$), 36.0 (*C*H$_2$), 35.1 (*C*H$_2$), 34.7 (*C*H$_2$), 34.5 (*C*H$_2$), 34.1 (*C*H$_2$), 33.9 (*C*H$_2$), 33.8 (*C*H$_2$), 33.8 (*C*H$_2$), 33.7 (*C*H$_2$), 33.6 (*C*H$_2$), 33.5 (*C*H$_2$), 33.2 (*C*H$_2$), 32.9 (*C*H$_2$), 32.5 (*C*H$_2$), 32.1 (*C*H$_2$), 31.4 (*C*H$_2$), 29.1 (*C*H$_2$), 28.0 (*C*H$_2$), 27.7 (*C*H$_2$), 27.6 (*C*H$_2$), 27.6 (*C*H$_2$), 27.4 (*C*H$_2$), 27.3 (*C*H$_2$), 27.2 (*C*H$_2$), 27.2 (*C*H$_2$), 27.2 (*C*H$_2$), 27.1 (*C*H$_2$), 27.1 (*C*H$_2$), 27.1 (*C*H$_2$), 27.0 (*C*H$_2$), 26.9 (*C*H$_2$), 26.7 (*C*H$_2$), 26.6 (*C*H$_2$), 26.6 (*C*H$_2$), 26.6 (*C*H$_2$), 26.5 (*C*H$_2$), 26.5 (*C*H$_2$), 26.4 (*C*H$_2$), 26.3 (*C*H$_2$), 26.3 (*C*H$_2$), 26.3 (*C*H$_2$), 26.2 (*C*H$_2$), 26.2 (*C*H$_2$), 26.1 (*C*H$_2$), 26.0 (*C*H$_2$), 25.7 (*C*H$_2$), 25.6 (*C*H$_2$), 25.4 (*C*H$_2$), 25.3 (*C*H$_2$), 25.1 (*C*H$_2$), 25.0 (*C*H$_2$), 24.8 (*C*H$_2$), 24.6 (*C*H$_2$) ppm. **^{11}B NMR** (192.6 MHz, *d*$_8$-toluene, 293.15 K): δ = 42.1 (br s), 34.4 (br s) ppm. **^{11}B NMR** (192.6 MHz, *d*$_8$-toluene, 193.15 K): δ = 36.3 (br s), 26.2 (br s) ppm. **^{19}F NMR** (564.7 MHz, *d*$_8$-toluene, 293.15 K): δ = –74.99, –76.29, –76.61, –76.93, –77.08, –78.20 ppm. **^{19}F NMR** (564.7 MHz, *d*$_8$-toluene, 193.15 K): δ = –75.41, –75.98, –76.16, –76.37, –76.48, –76.66, –76.76, –76.83, –77.17, –77.39, –78.01 ppm. **HRMS LIFDI** for $[C_{49}H_{88}B_4F_3N_4O_3S_2]^+$ = $[M – CSF_3O_3]^+$: calc. 945.6617; found 945.6616.

Synthesis of $B_4(NCy_2)_4F_2$ (5F). $B_4(NCy_2)_4$ (**1**, 50.0 mg, 65.4 μmol) and SbF_3 (23.4 mg, 131 μmol, 2.0 equiv.) were combined in a flask (silanized with HMDS) and dissolved in benzene (5 mL). After stirring for 20 h at 85 °C, the initially blue solution underwent decolorization. The insoluble materials in the reaction mixture were subsequently removed by filtration. After removal of all volatiles from the reaction mixture in vacuo, the residue was washed with DME (4 × 2 mL), filtered, and dried under reduced pressure. This yielded product **5F** as a colorless solid (25.0 mg, 31.2 μmol, 47%). Single crystals suitable for X-ray diffraction analysis were obtained by crystallization in benzene at room temperature.

Note: In solution, a mixture of temperature-dependent conformational isomers is present, which cannot be distinguished from one another.

$^{1}H\{^{11}B\}$ NMR (600.1 MHz, d_8-toluene, 383.15 K): δ = 3.41–3.33 (m, 2H, C*H*), 3.30–3.19 (m, 2H, C*H*), 3.16–3.08 (m, 2H, C*H*), 2.72–2.63 (m, 2H, CH_2), 2.20–2.09 (m, 2H, CH_2), 2.01–0.99 (m, 76H, CH_2) ppm. **$^{13}C\{^{1}H\}$ NMR** (150.9 MHz, d_8-toluene, 383.15 K): δ = 68.1 (*C*H), 62.9 (*C*H), 58.6 (*C*H), 58.6 (*C*H), 55.5 (*C*H), 37.5 (CH_2), 35.4 (CH_2), 33.7 (CH_2), 28.0 (CH_2), 27.7 (CH_2), 27.5 (CH_2), 27.1 (CH_2), 26.8 (CH_2), 26.7 (CH_2), 26.6 (CH_2) ppm. **^{11}B NMR** (192.6 MHz, d_8-toluene, 383.15 K): δ = 56.9 (br s), 37.9 (br s) ppm. **^{19}F NMR** (564.7 MHz, d_8-toluene, 403.15 K): δ = –79.81 ppm. **^{19}F NMR** (564.7 MHz, d_8-toluene, 298.15 K): δ = –76.18, –77.04, –84.96, –87.05, –88.53 ppm. **HRMS LIFDI** for $[C_{48}H_{88}B_4F_2N_4]^+$ = $[M]^+$: calc. 802.7344; found 802.7329.

Synthesis of $B_4(NCy_2)_4Cl_2$ (5Cl). $B_4(NCy_2)_4$ (**1**, 50 mg, 65.4 μmol) and hexachloroethane (15.5 mg, 65.4 μmol, 1.0 equiv.) were combined in benzene (3 mL) and stirred for 3 h at 60 °C, resulting in the decolorization of the blue solution. The insoluble materials in the reaction mixture were subsequently removed by filtration, and the solvent of the filtrate was removed under reduced pressure. This produced **5Cl** as a colorless solid (43.0 mg, 51.5 μmol, 78%). Single crystals suitable for X-ray diffraction analysis were obtained by crystallization in DME at –30 °C.

Note: Selective oxidation of **1** to **5Cl** was also observed when **1** was reacted with one equivalent of $AlCl_3$ or $GaCl_3$.

$^{1}H\{^{11}B\}$ NMR (600.1 MHz, C_6D_6, 297 K): δ = 3.97–3.88 (m, 2H, C*H*), 3.79–3.67 (m, 2H, C*H*), 3.60–3.52 (m, 2H, C*H*), 3.06–2.54 (m, 4H, C*H* and CH_2), 2.43–0.93 (m, 78H, CH_2) ppm. **$^{13}C\{^{1}H\}$ NMR** (150.9 MHz, d_8-toluene, 383.15 K): δ = 68.0 (*C*H), 67.1 (*C*H), 63.7 (*C*H), 57.8 (*C*H), 39.2 (CH_2), 38.7 (CH_2), 35.8 (CH_2), 35.4 (CH_2), 34.5 (CH_2), 34.1 (CH_2), 33.6 (CH_2), 28.0 (CH_2), 28.0 (CH_2), 27.9 (CH_2), 27.9 (CH_2), 27.8 (CH_2), 27.2 (CH_2), 27.1 (CH_2), 27.0 (CH_2), 26.7 (CH_2), 26.5 (CH_2), 26.4 (CH_2) ppm. **^{11}B NMR** (192.6 MHz, d_8-toluene, 383.15 K): δ = 57.5 (br s), 43.4 (br s) ppm. **HRMS LIFDI** for $[C_{48}H_{88}B_4Cl_2N_4]^+$ = $[M]^+$: calc. 834.6751; found 834.6753.

Synthesis of $B_4(NCy_2)_4Br_2$ (5Br). To a solution of $B_4(NCy_2)_4$ (**1**, 50 mg, 65.4 μmol) in pentane (20 mL) cooled to –60 °C, a pentane solution (5 mL) of Br_2 (10.5 mg, 0.20 mL of 0.325 M in benzene 65.4 μmol, 1.0 equiv.) at –60 °C was added dropwise. The reaction mixture was stirred for 24 h while slowly warming to room temperature, resulting in the decolorization of the blue solution. After removal of all volatiles from the reaction mixture in vacuo, the residue was

washed with cold hexane (2 mL, –60 °C) and dried under reduced pressure. This yielded **5Br** as a colorless solid (43.0 mg, 46.5 µmol, 71%). Single crystals for X-ray diffraction analysis were obtained by crystallization in pentane at –30 °C.

^{1}H{^{11}B} NMR (600.1 MHz, d_8-toluene, 383.15 K): δ = 3.94–3.88 (m, 2H, C*H*), 3.65–3.56 (m, 4H, C*H*), 3.51–3.27 (m, 2H, C*H*), 3.63–2.44 (m, 2H, C*H*$_2$), 3.43–2.35 (m, 2H, C*H*$_2$), 2.28–2.20 (m, 2H, C*H*$_2$), 2.15–2.09 (m, 2H, C*H*$_2$), 1.94–0.99 (m, 72H, C*H*$_2$) ppm. **^{13}C{^{1}H} NMR** (150.9 MHz, d_8-toluene, 383.15 K): δ = 68.6 (*C*H), 66.8 (*C*H), 64.8 (*C*H), 59.3 (*C*H), 39.0 (*C*H$_2$), 38.9 (*C*H$_2$), 35.9 (*C*H$_2$), 35.4 (*C*H$_2$), 34.5 (*C*H$_2$), 34.1 (*C*H$_2$), 33.8 (*C*H$_2$), 28.0 (*C*H$_2$), 27.9 (*C*H$_2$), 27.8 (*C*H$_2$), 27.7(*C*H$_2$), 27.1 (*C*H$_2$), 27.0 (*C*H$_2$), 26.9 (*C*H$_2$), 26.7 (*C*H$_2$), 26.5 (*C*H$_2$), 26.4 (*C*H$_2$) ppm. **^{11}B NMR** (192.6 MHz, d_8-toluene, 383.15 K): δ = 57.0 (br s), 43.3 (br s) ppm. **HRMS LIFDI** for $[C_{48}H_{88}B_4BrN_4]^+$ = $[M - Br]^+$: calc. 844.6575; found 844.6564.

Synthesis of $B_4(NCy_2)_4(OTf)_2$ (5OTf). $B_4(NCy_2)_4$ (**1**, 50 mg, 65.4 µmol) and AgOTf (33.6 mg, 130.8 µmol, 2.0 equiv.) were combined in benzene (4 mL) and stirred for 1 h at room temperature, resulting in the decolorization of the blue solution. The insoluble materials in the reaction mixture were subsequently separated by filtration, and the solvent of the filtrate was removed under reduced pressure, yielding **5OTf** as a colorless solid (64.0 mg, 65.4 µmol, 92%).

Note: Single crystals of **5OTf** suitable for X-ray diffraction analysis could not be obtained despite numerous crystallization attempts. Attempts involved systematic variations of solvents, temperatures, and crystallization techniques, yet the resulting crystals consistently exhibited excessive disorder, preventing reliable X-ray diffraction analysis. Note: In solution, a mixture of temperature-dependent conformational isomers is present, which cannot be distinguished from one another.

^{1}H{^{11}B} NMR (600.1 MHz, C_6D_6, 393.15 K): δ = 4.01–3.94 (m, 1H, C*H*), 3.76–3.55 (m, 4H, C*H*), 3.29–3.22 (m, 1H, C*H*), 3.16–3.05 (m, 1H, C*H*), 3.00–2.92 (m, 1H, C*H*), 2.54–2.43 (m, 3H, C*H*$_2$), 2.37–2.31 (m, 1H, C*H*$_2$), 2.25–2.16 (m, 4H, C*H*$_2$), 2.06–1.02 (m, 72H, C*H*$_2$) ppm. **^{11}B NMR** (192.6 MHz, d_8-toluene, 393.15 K): δ = 55.5 (br), 36.2 (br) ppm. **^{13}C{^{1}H,^{11}B} NMR** (150.9 MHz, d_8-toluene, 383.15 K): δ = 120.7 (*C*F$_3$), 118.6 (*C*F$_3$), 71.8 (*C*H), 67.6 (*C*H), 67.5 (*C*H), 60.1 (*C*H), 60.1 (*C*H), 57.4 (*C*H), 56.8 (*C*H), 39.0 (*C*H$_2$), 38.7 (*C*H$_2$), 38.1 (*C*H$_2$), 37.5 (*C*H$_2$), 36.3 (*C*H$_2$), 36.1 (*C*H$_2$), 35.8 (*C*H$_2$), 34.2 (*C*H$_2$), 33.6 (*C*H$_2$), 31.2 (*C*H$_2$), 30.2 (*C*H$_2$), 28.5 (*C*H$_2$), 28.4 (*C*H$_2$), 28.2 (*C*H$_2$), 27.9 (*C*H$_2$), 27.8 (*C*H$_2$), 27.6 (*C*H$_2$), 27.2 (*C*H$_2$), 27.0 (*C*H$_2$), 26.9 (*C*H$_2$), 26.7 (*C*H$_2$), 26.3 (*C*H$_2$), 26.2 (*C*H$_2$), 26.0 (*C*H$_2$), 25.9 (*C*H$_2$), 25.9 (*C*H$_2$) ppm. **^{19}F NMR**

(470.6 MHz, d_8-toluene, 297.15 K): δ = –75.66, –75.83, –75.95, –76.20, –77.26, –78.10 ppm. **^{19}F NMR** (564.7 MHz, d_8-toluene, 173.15 K): δ = –76.20, –77.62, –77.81 ppm. **HRMS LIFDI** for $[C_{49}H_{88}B_4N_4F_3O_3S_1]^+$ = $[M – CSF_3O_3]^+$: calc. 913.6896; found 913.6889.

Synthesis of $B_2(NCy_2)_2Br_2$ (6). To a solution of $B_4(NCy_2)_4$ (**1**, 50 mg, 65.4 µmol) in benzene (10 mL), a chilled solution of Br_2 (20.9 mg, 0.40 mL of 0.235 M in benzene, 130 µmol, 2.0 equiv.) in benzene at 0 °C was added and stirred for 2 h. During this time, decolorization of the blue reaction mixture was observed. After removal of all volatiles from the reaction mixture in vacuo, the residue was washed with cold hexane (2 mL, –30 °C) and dried under reduced pressure, yielding **6** as a colorless solid (39.8 mg, 73.4 µmol, 56%). Single crystals suitable for X-ray diffraction analysis were obtained by crystallization in hexane at –30 °C.

^{1}H{^{11}B} NMR (600.1 MHz, d_8-toluene, 383.15 K): δ = 3.36–3.30 (m, 2H, C*H*), 3.28–3.21 (m, 2H, C*H*), 2.44–2.33 (m, 4H, CH_2), 1.99–1.93 (m, 2H, CH_2), 1.72–1.60 (m, 16H, CH_2), 1.52–1.41 (m, 6H, CH_2), 1.29–1.06 (m, 10H, CH_2), 1.02–0.91 (m, 2H, CH_2) ppm. **^{13}C{^{1}H} NMR** (150.9 MHz, d_8-toluene, 383.15 K): δ = 65.5 (*C*H), 58.8 (*C*H), 33.6 (CH_2), 33.5 (CH_2), 27.1 (CH_2), 27.0 (CH_2), 26.7 (CH_2), 26.5 (CH_2), 25.9 (CH_2), 25.8 (CH_2) ppm. **^{11}B NMR** (192.6 MHz, d_8-toluene, 383.15 K): δ = 37.8 (s) ppm. **HRMS LIFDI** for $[C_{24}H_{44}B_2Br_2N_2]^+$ = $[M]^+$: calc. 542.2031; found 542.2018.

NMR spectra

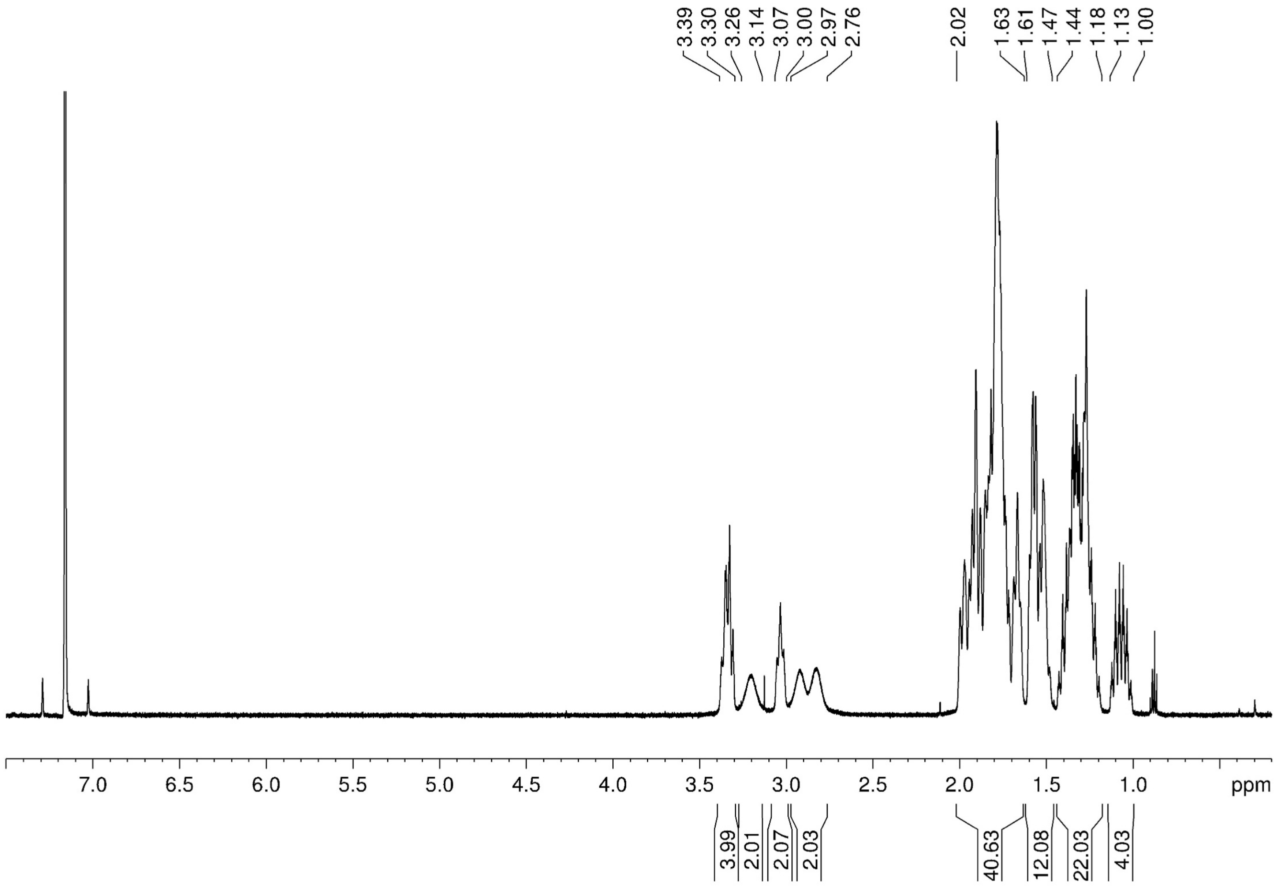

Supplementary Fig. S1 | $^{1}H\{^{11}B\}$ NMR (600.1 MHz) spectrum **2S** in C_6D_6.

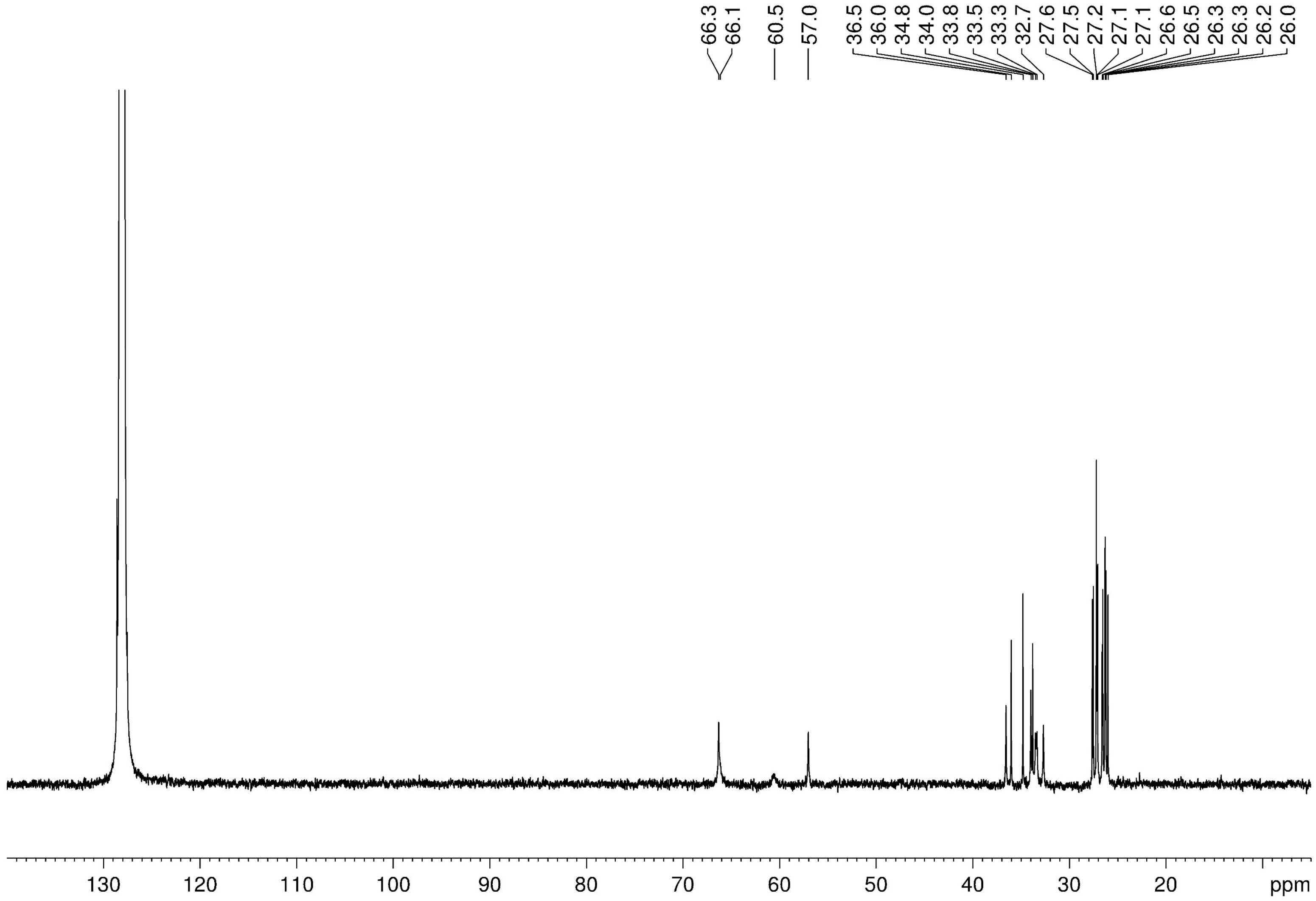

Supplementary Fig. S2 | $^{13}C\{^1H\}$ NMR (125.7 MHz) spectrum of **2S** in C_6D_6.

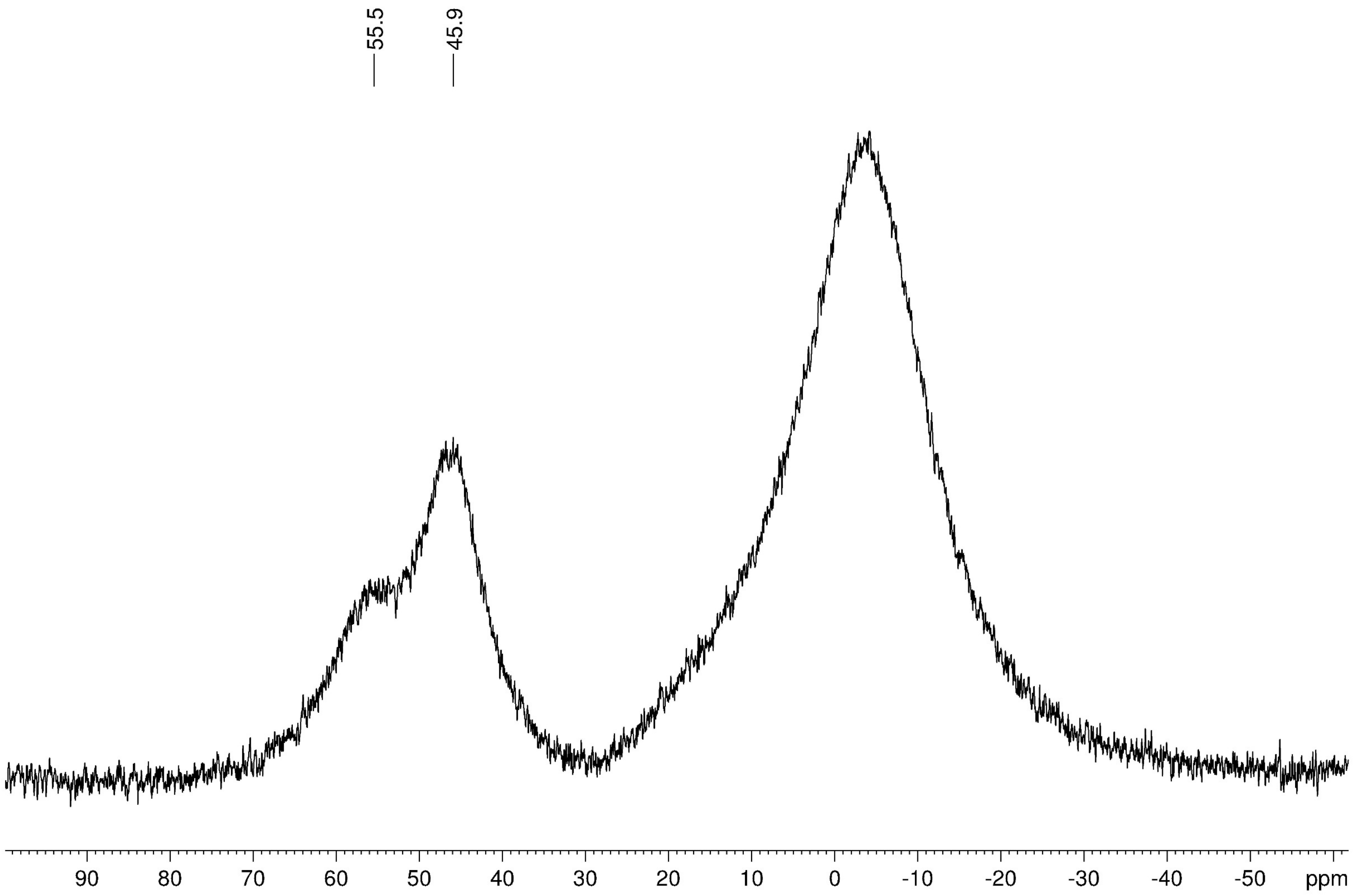

Supplementary Fig. S3 | ^{11}B NMR (160.5 MHz) spectrum of **2S** in C_6D_6.

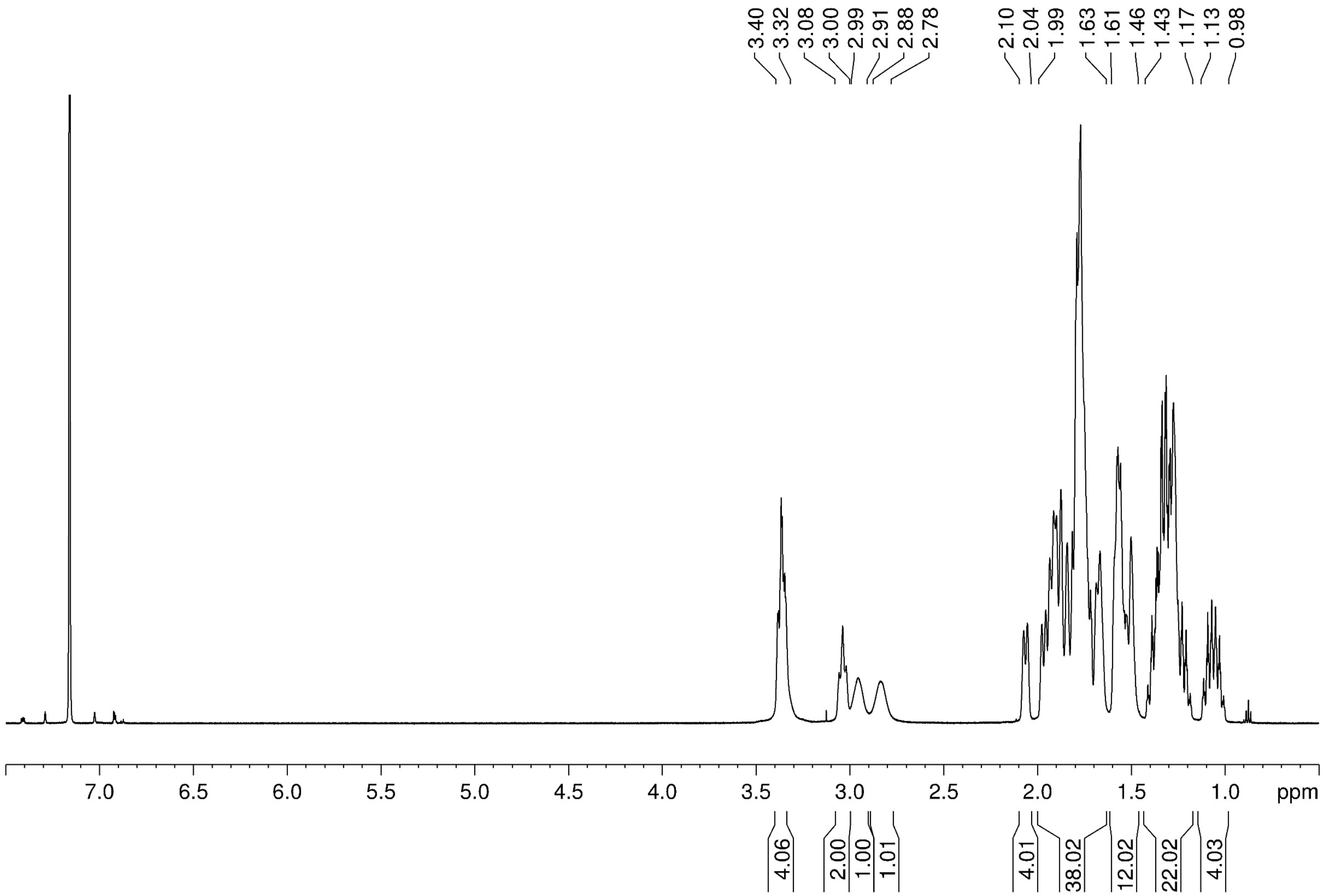

Supplementary Fig. S4 | $^{1}H\{^{11}B\}$ NMR (600.1 MHz) spectrum of **2Se** in C_6D_6.

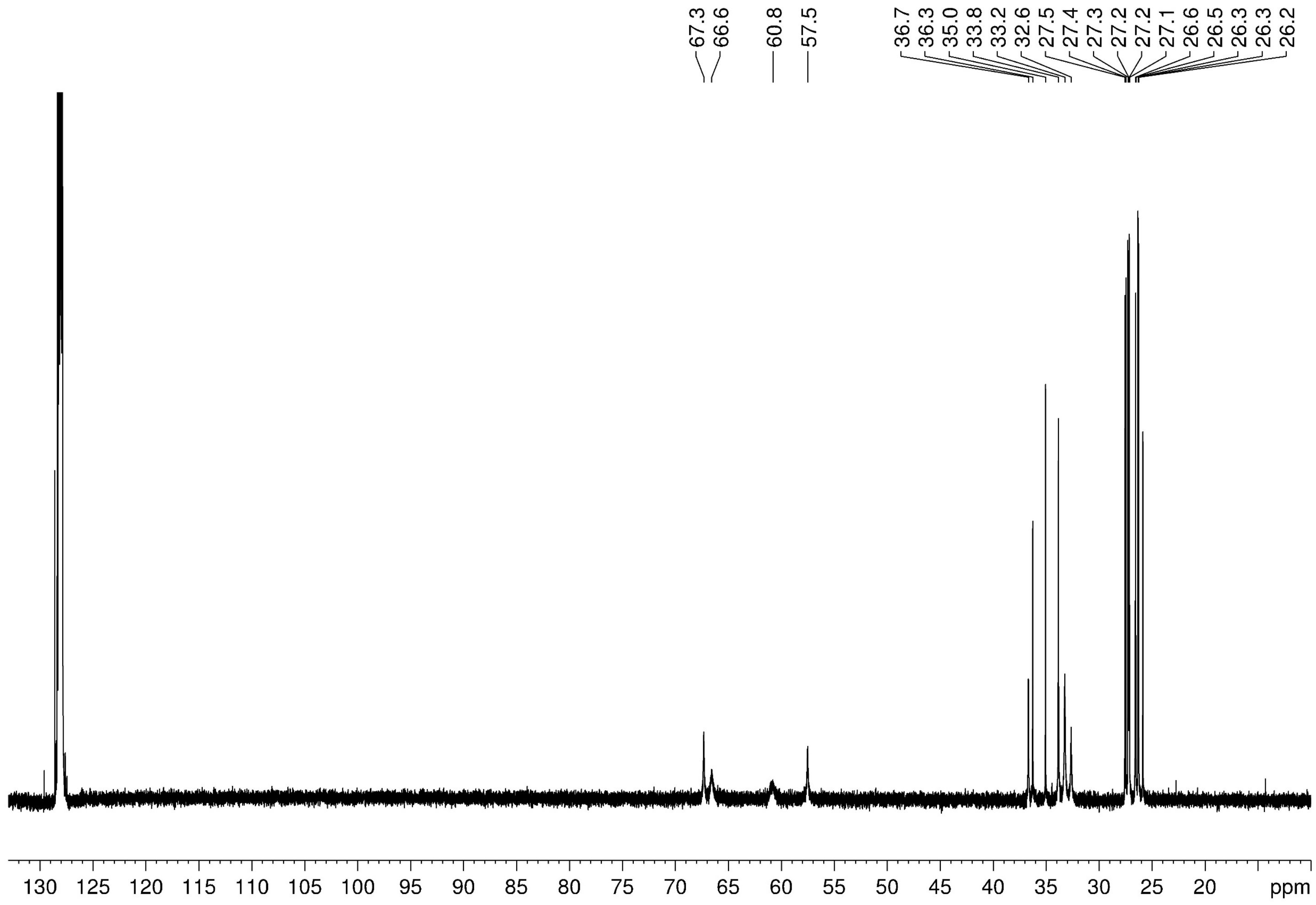

Supplementary Fig. S5 | $^{13}C\{^{1}H\}$ NMR (150.9 MHz) spectrum of **2Se** in C_6D_6.

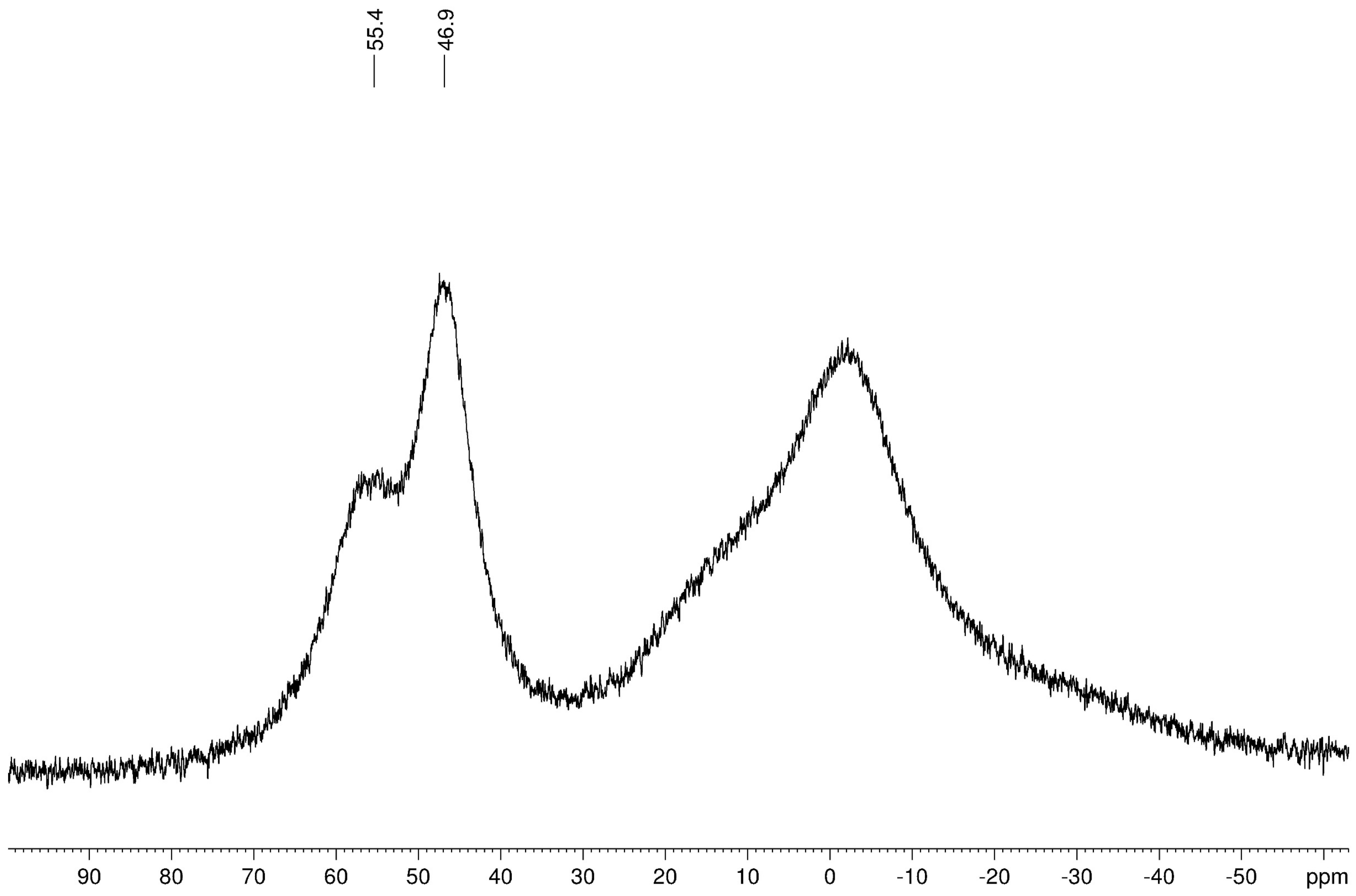

Supplementary Fig. S6 | ^{11}B NMR (192.6 MHz) spectrum of **2Se** in C_6D_6.

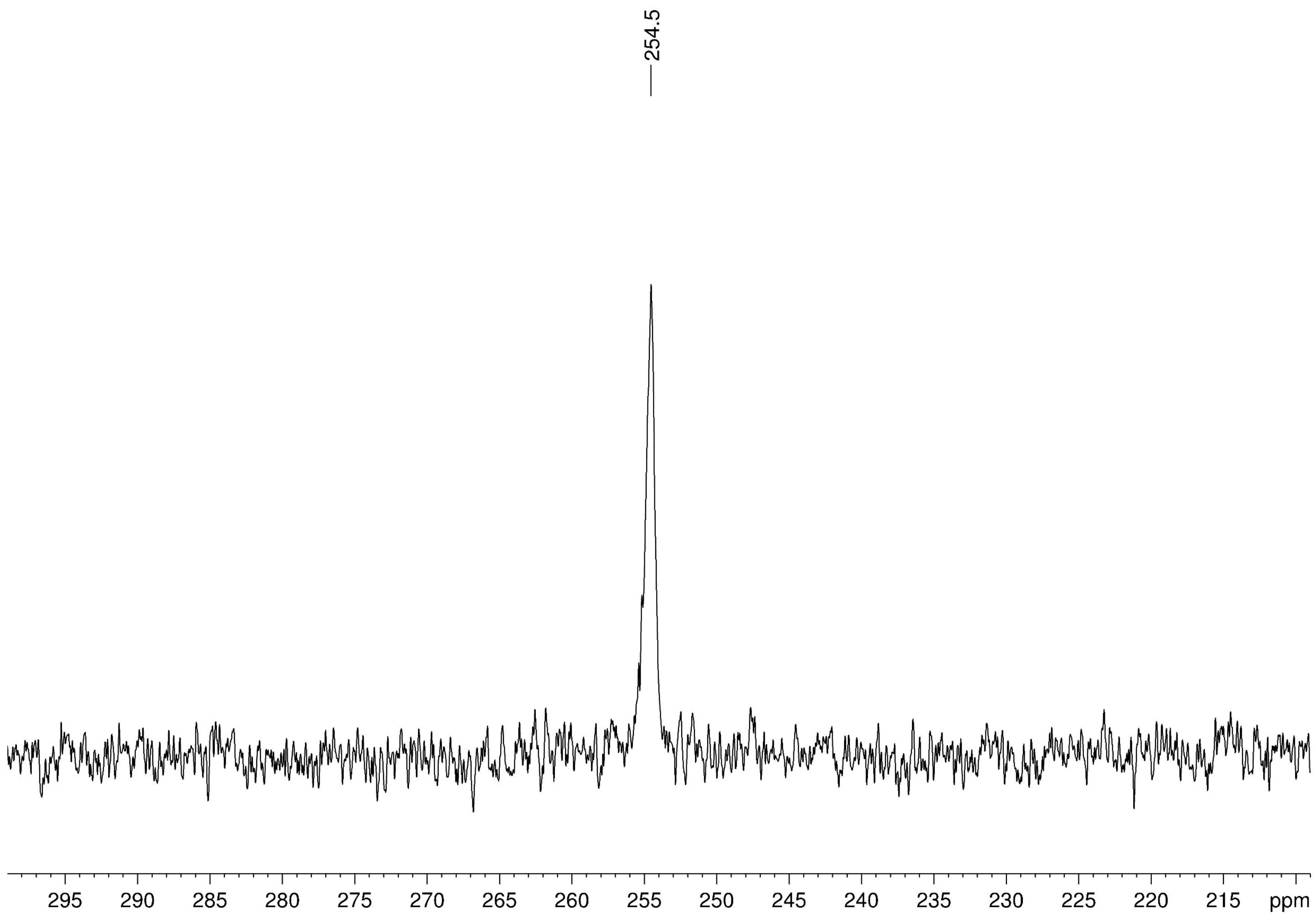

Supplementary Fig. S7 | $^{77}Se\{^{1}H,^{11}B\}$ NMR (114.5 MHz) spectrum of **2Se** in C_6D_6.

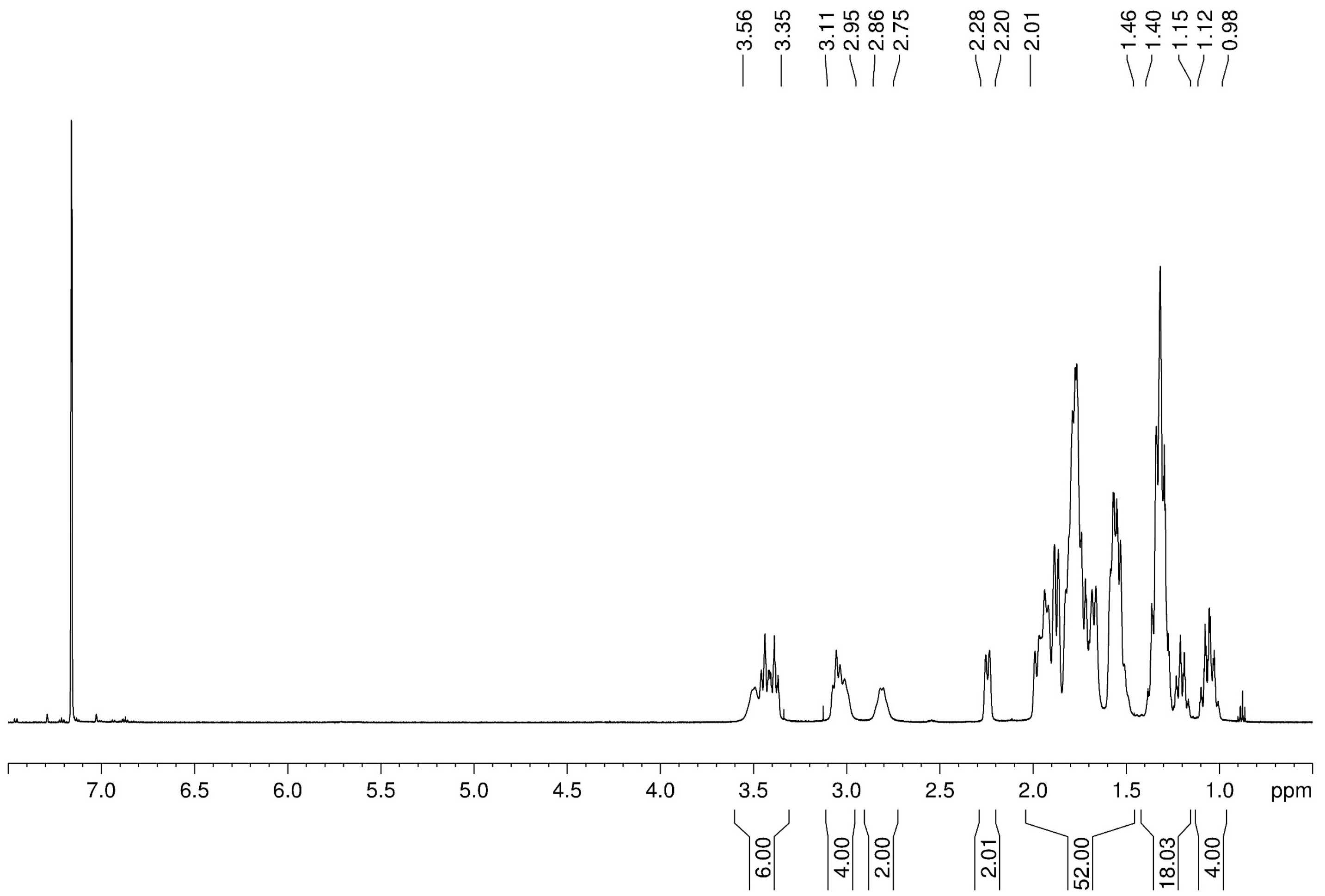

Supplementary Fig. S8 | $^{1}H\{^{11}B\}$ NMR (600.1 MHz) spectrum of **2Te** in C_6D_6.

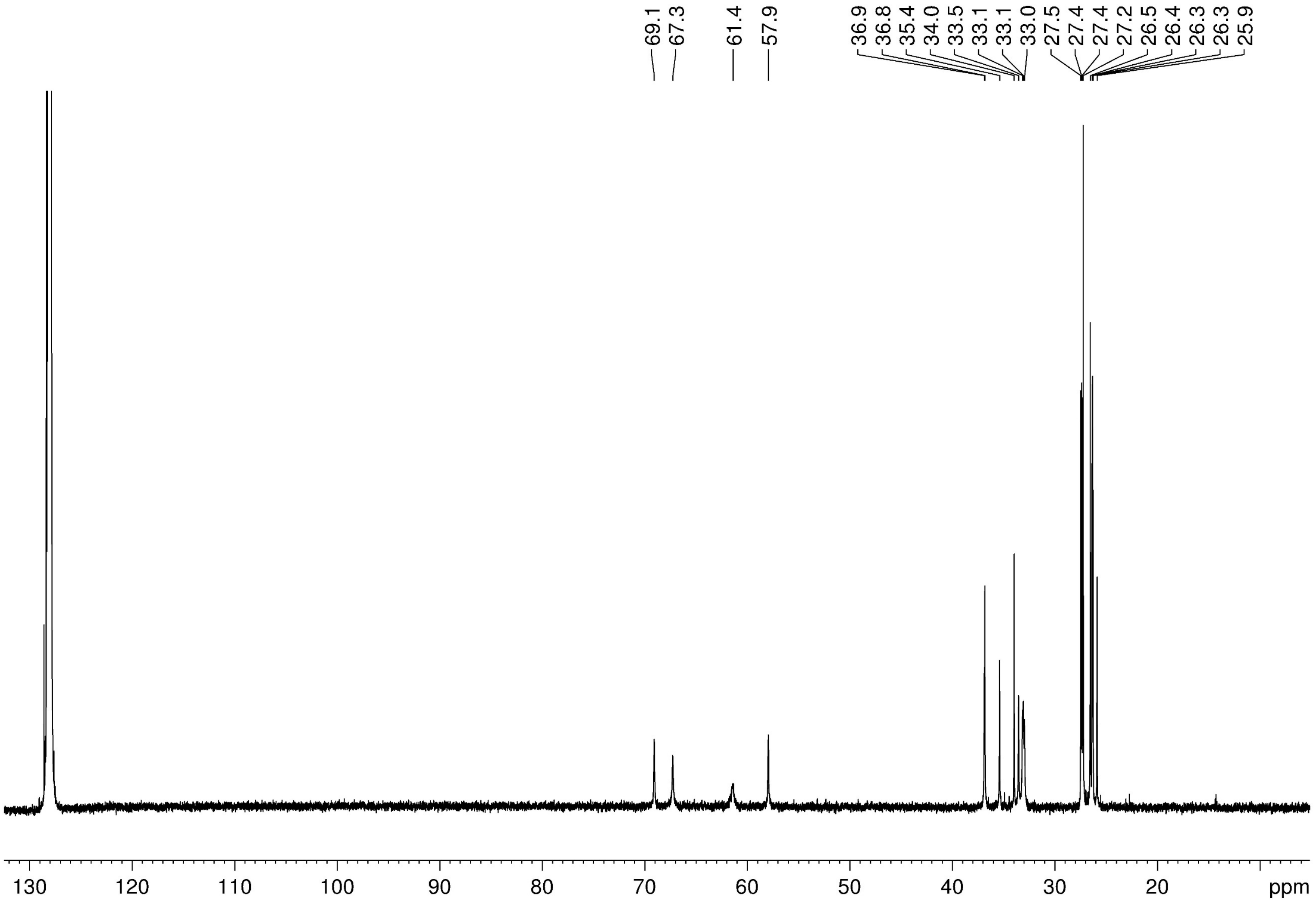

Supplementary Fig. S9 | $^{13}C\{^1H\}$ NMR (150.9 MHz) spectrum of **2Te** in C_6D_6.

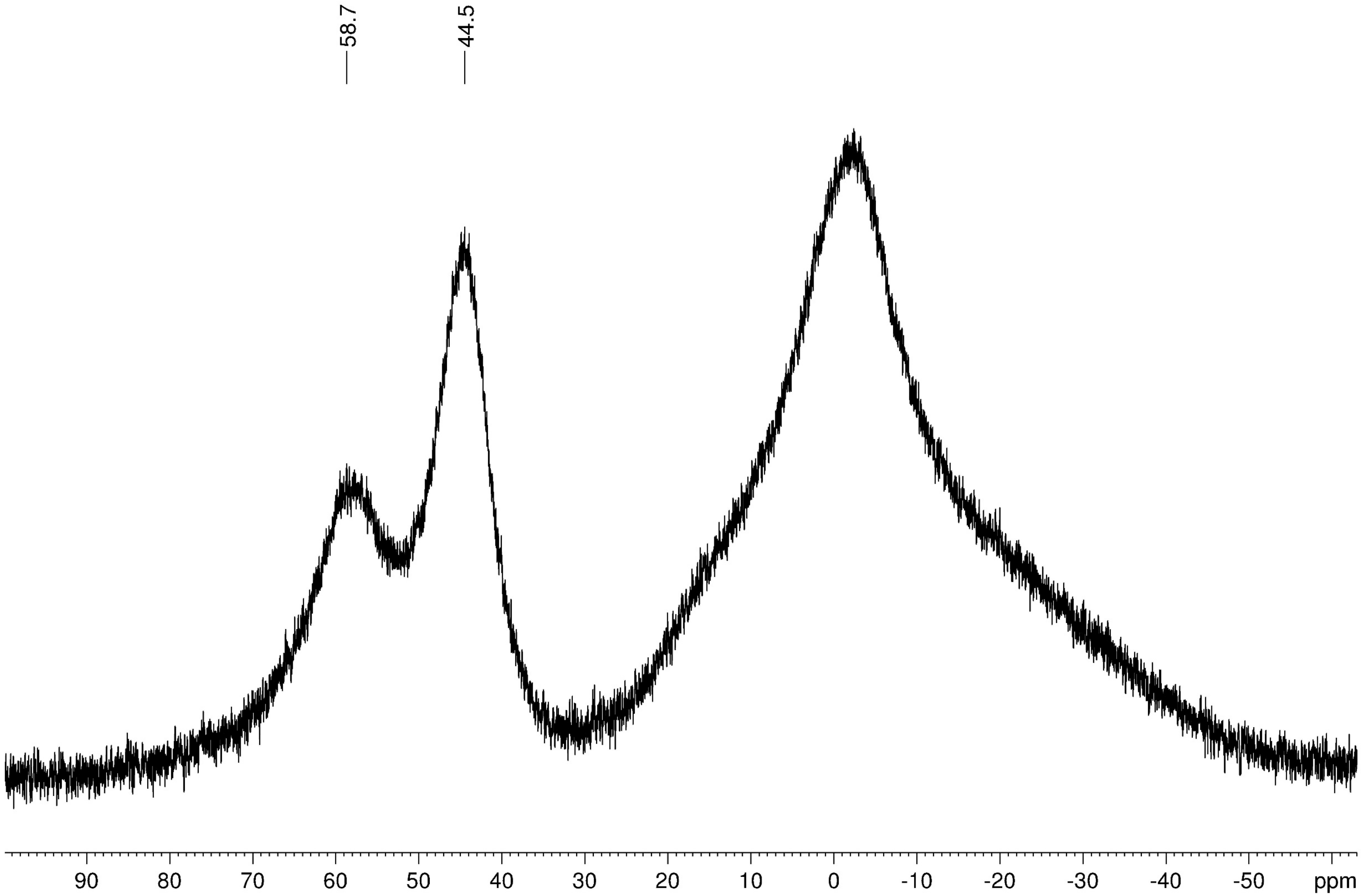

Supplementary Fig. S10 | ^{11}B NMR (192.6 MHz) spectrum of **2Te** in C_6D_6.

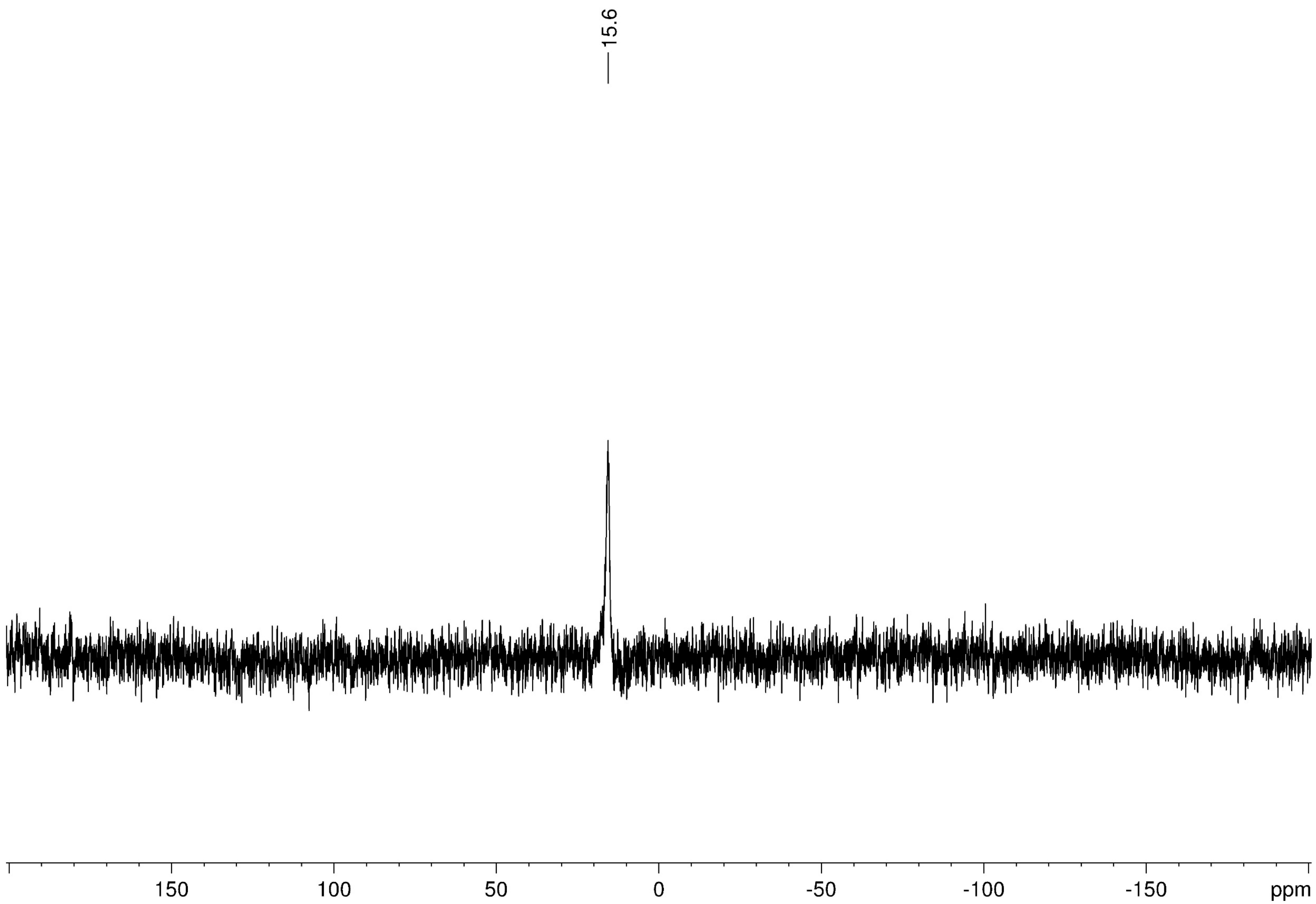

Supplementary Fig. S11 | ^{125}Te NMR (189.3 MHz) spectrum of **2Te** in C_6D_6.

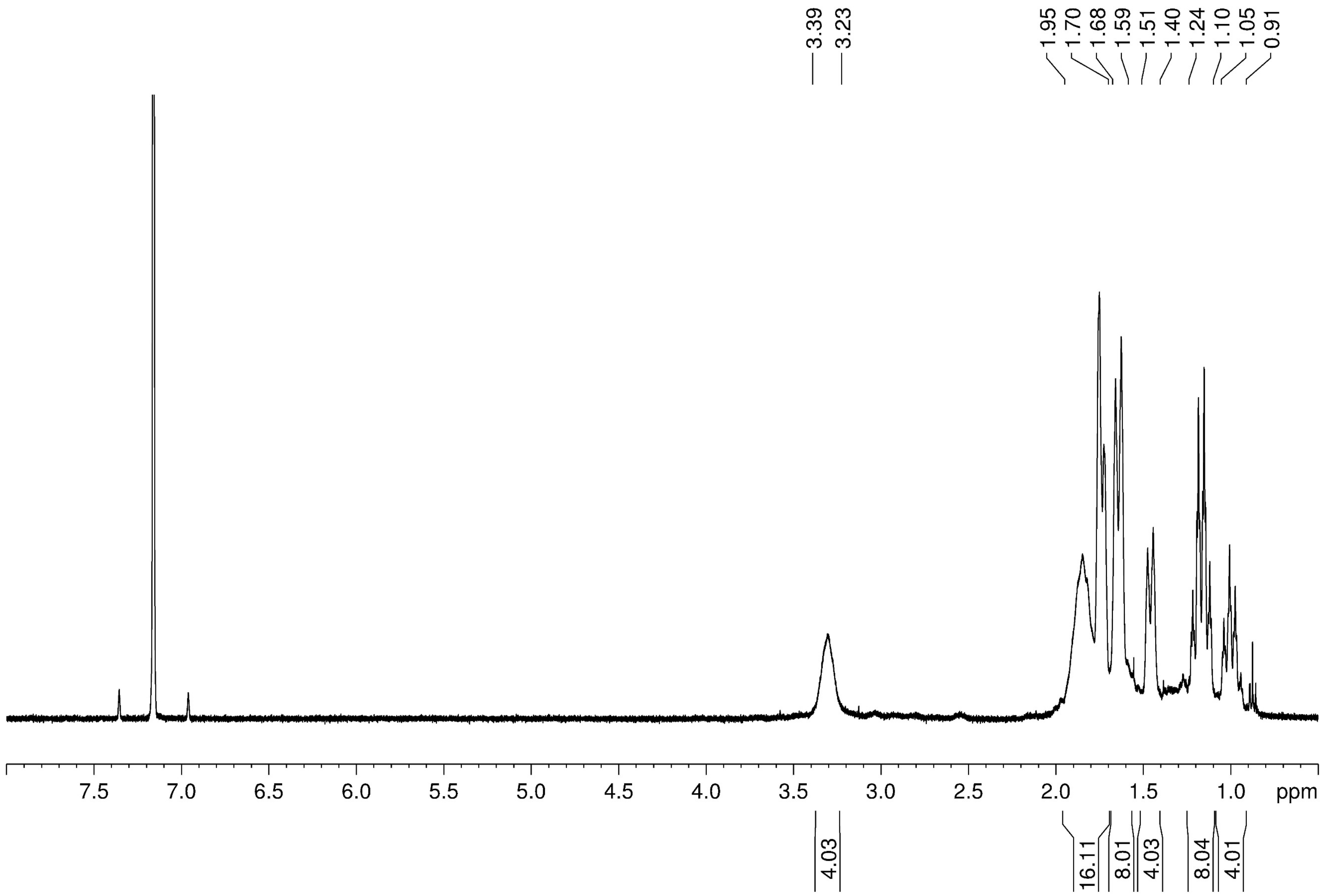

Supplementary Fig. S12 | $^{1}H\{^{11}B\}$ NMR (400.1 MHz) spectrum of **3S** in C_6D_6.

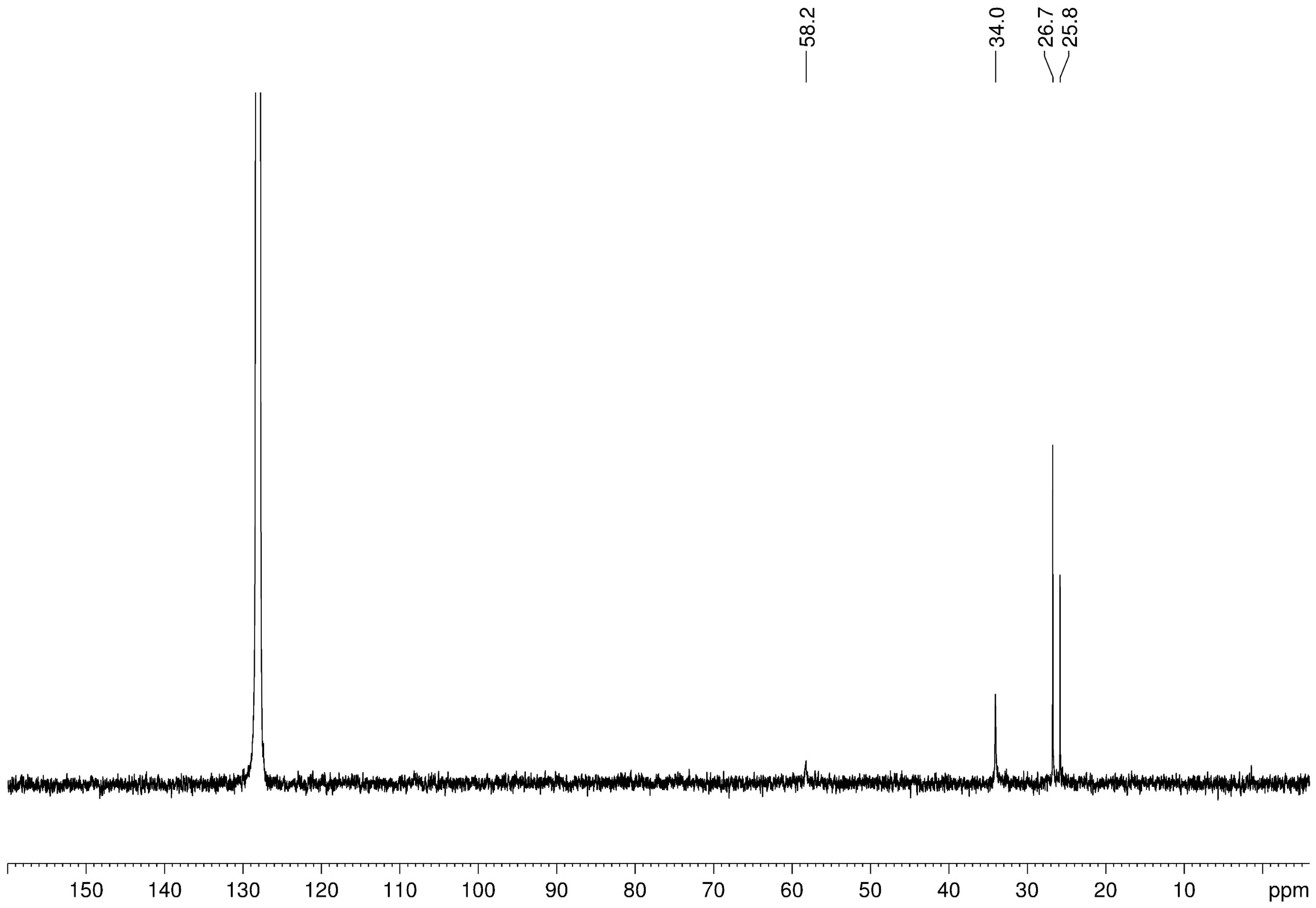

Supplementary Fig. S13 | $^{13}C\{^1H\}$ NMR (100.7 MHz) spectrum of **3S** in C_6D_6.

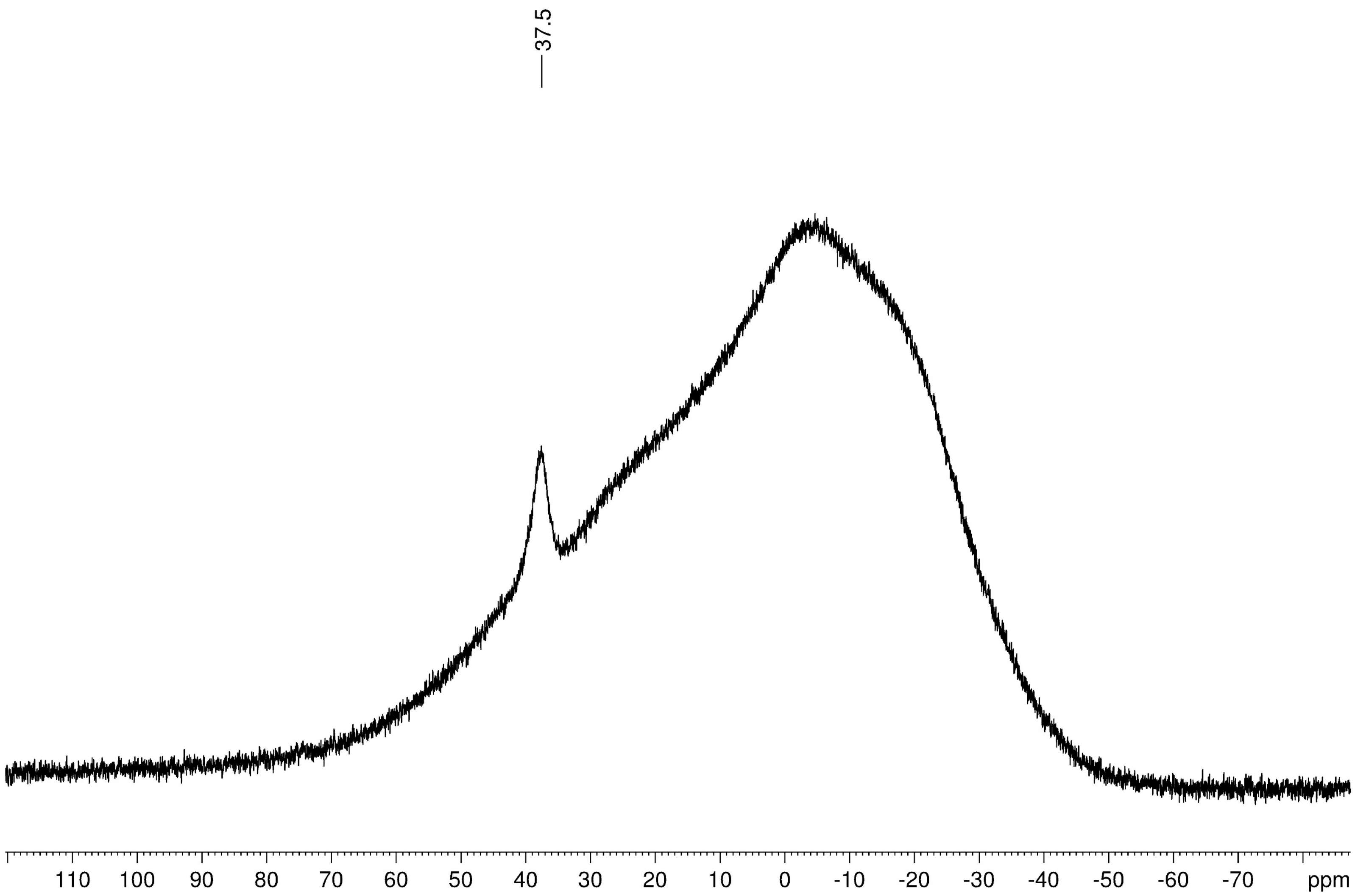

Supplementary Fig. S14 | ^{11}B NMR (128.5 MHz) spectrum of **3S** in C_6D_6.

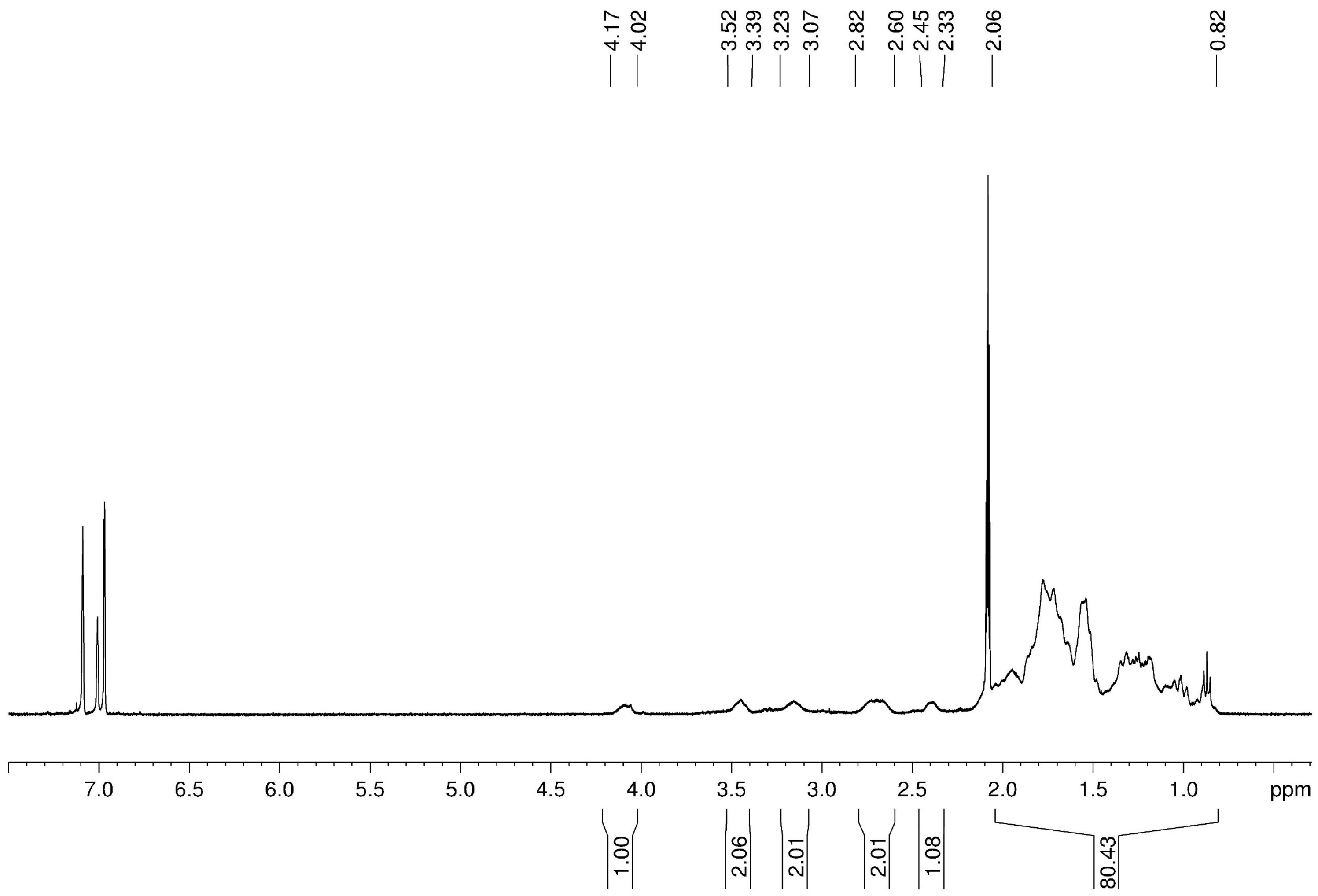

Supplementary Fig. S15 | $^{1}H\{^{11}B\}$ NMR (600.1 MHz) spectrum of **4S** in *d*$_8$-toluene.

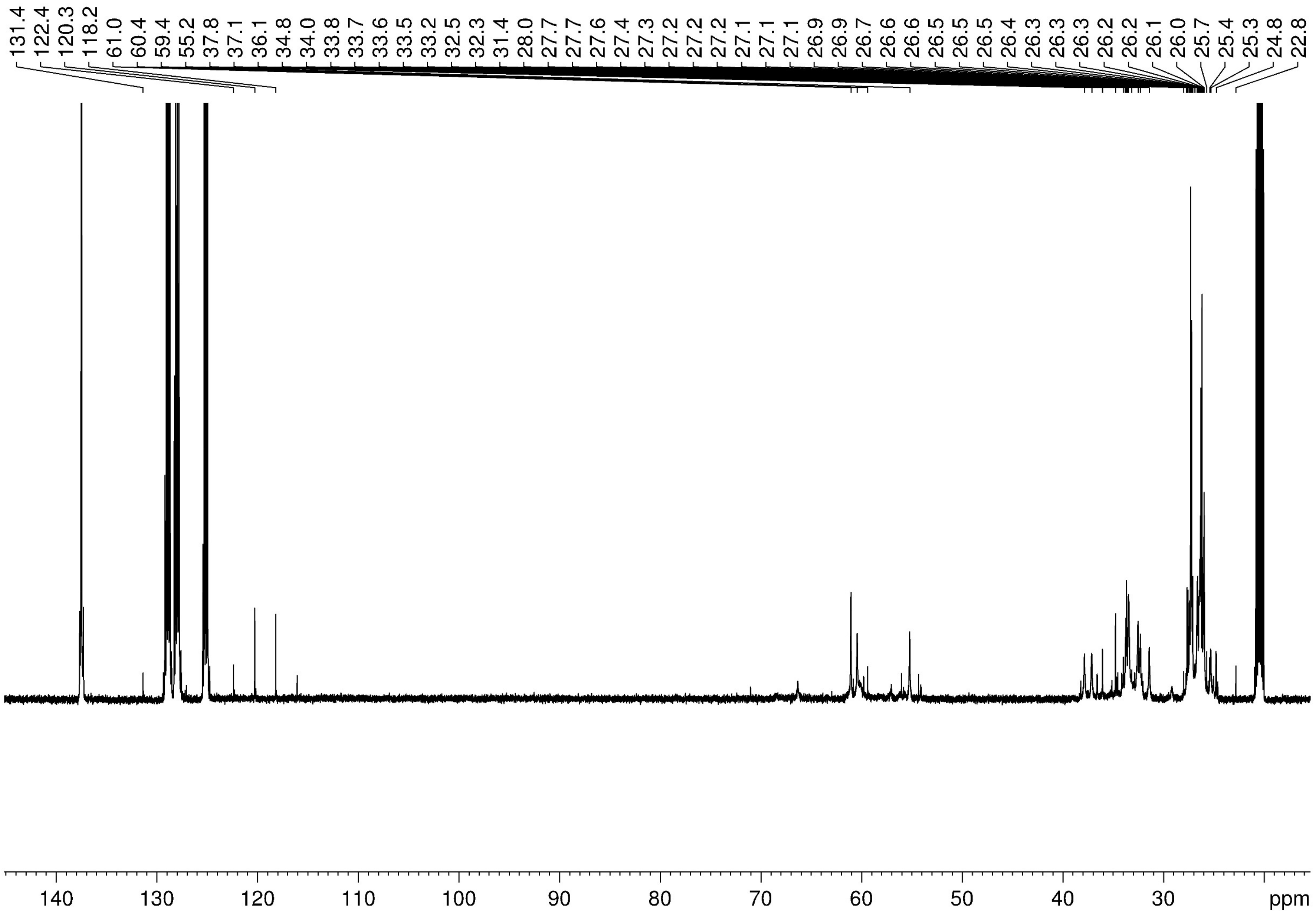

Supplementary Fig. S16 | $^{13}C\{^1H\}$ NMR (150.9 MHz) spectrum of **4S** in d_8-toluene.

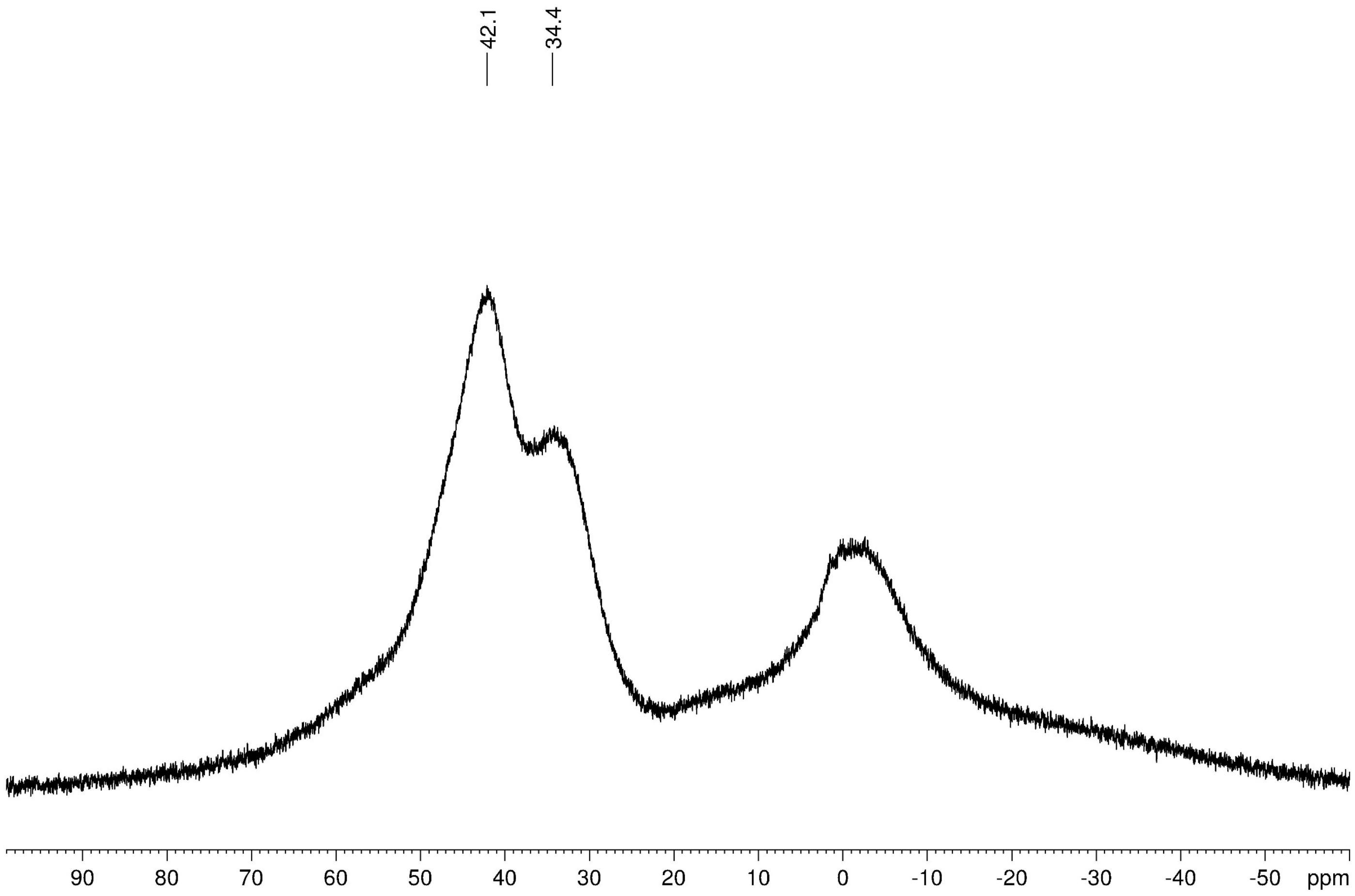

Supplementary Fig. S17 | ^{11}B NMR (192.6 MHz) spectrum of **4S** in d_8-toluene at 293.15 K.

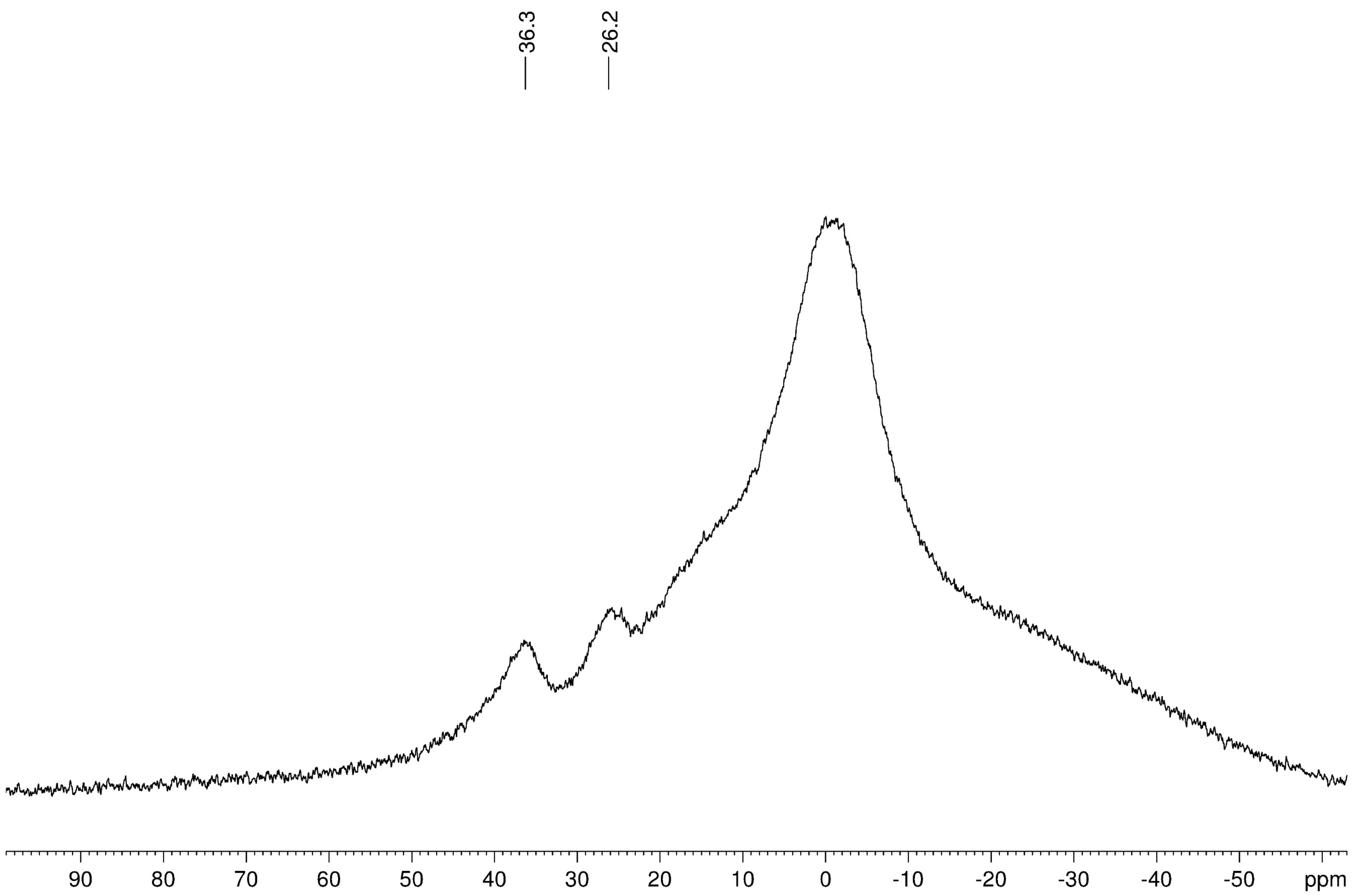

Supplementary Fig. S18 | ^{11}B NMR (192.6 MHz) spectrum of **4S** in *d$_8$*-toluene at 193.15 K.

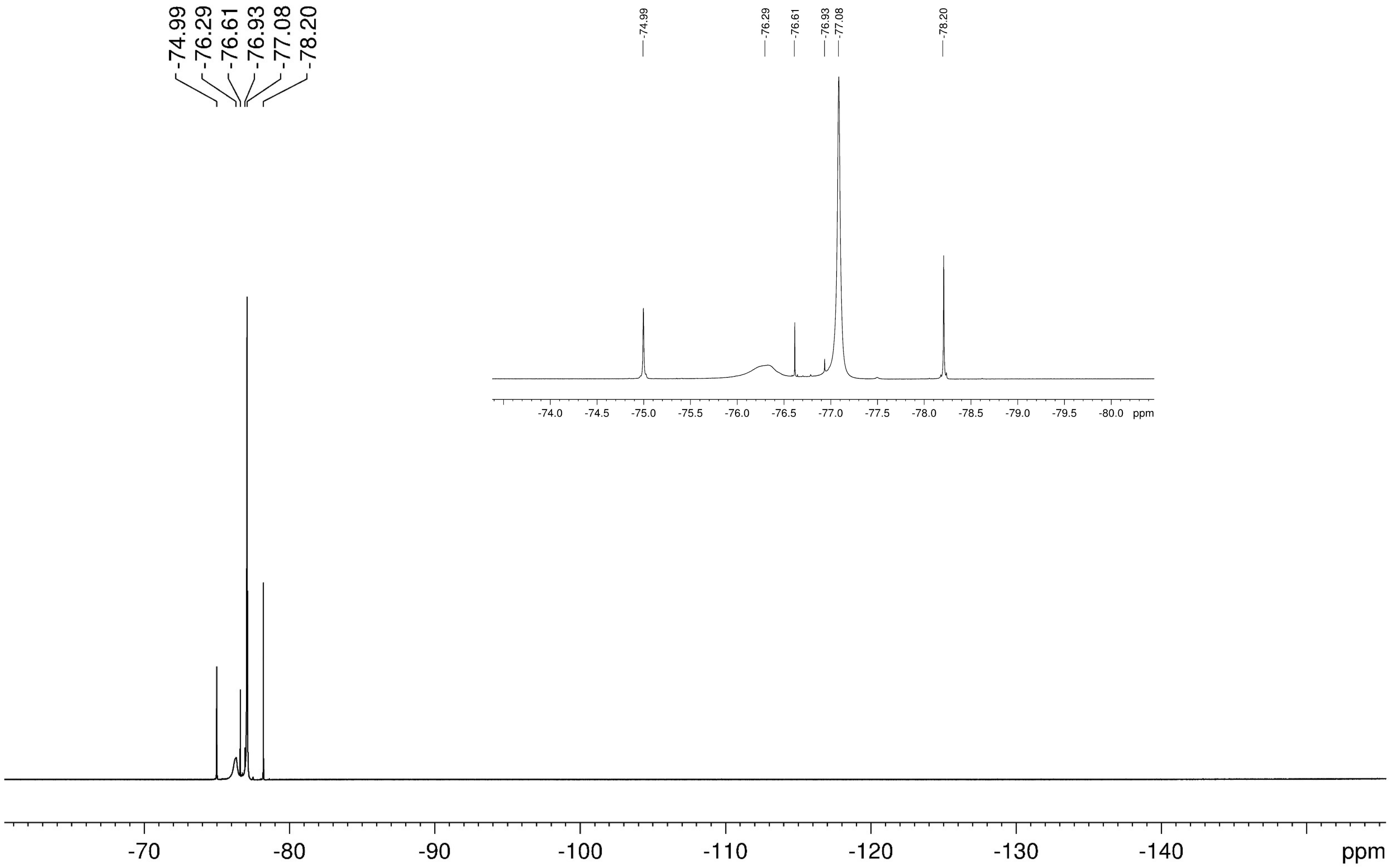

Supplementary Fig. S19 | ^{19}F NMR (564.7 MHz) spectrum of **4S** in d_8-toluene.

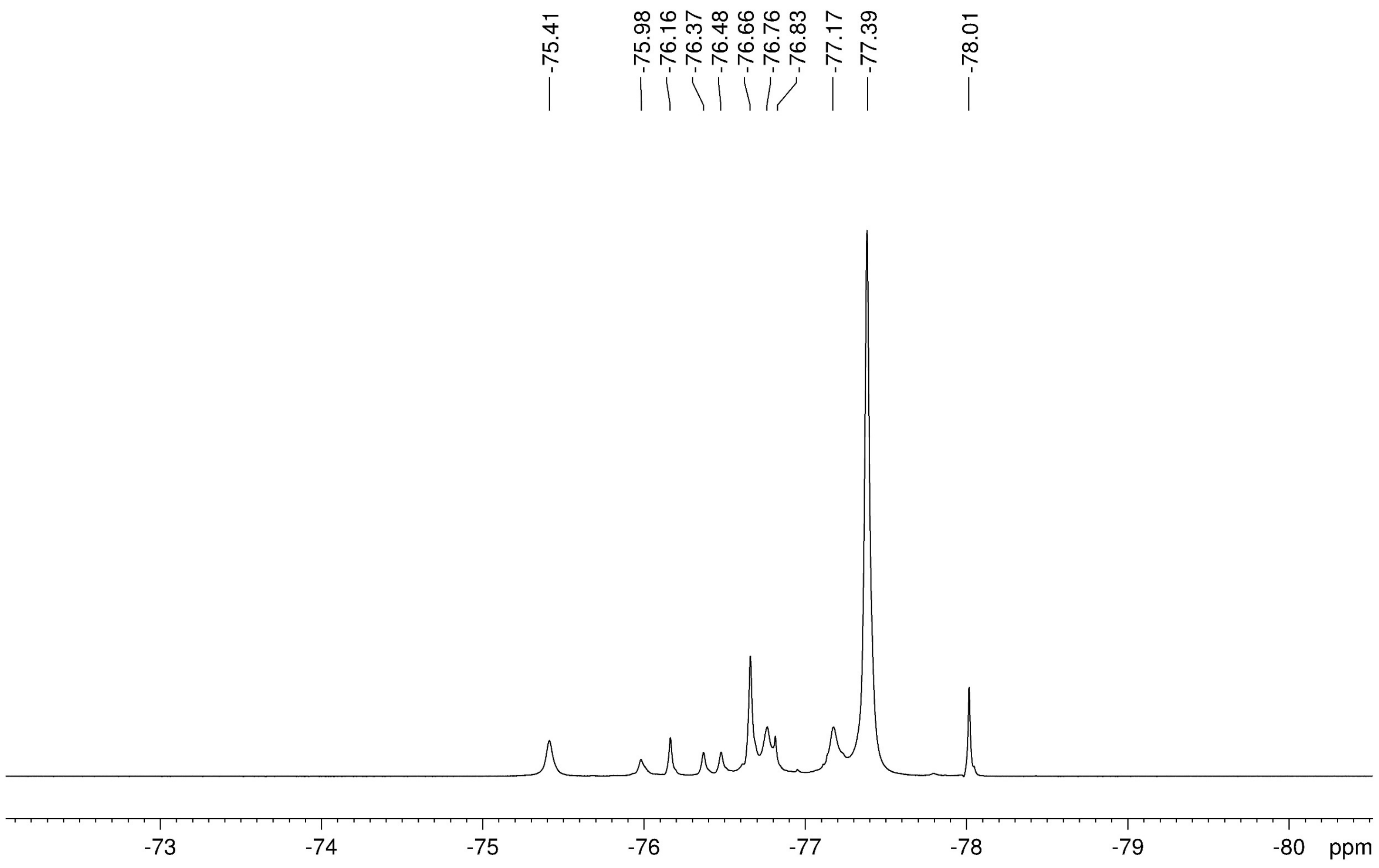

Supplementary Fig. S20 | ^{19}F NMR (564.7 MHz) spectrum of **4S** in d_8-toluene at 193 K.

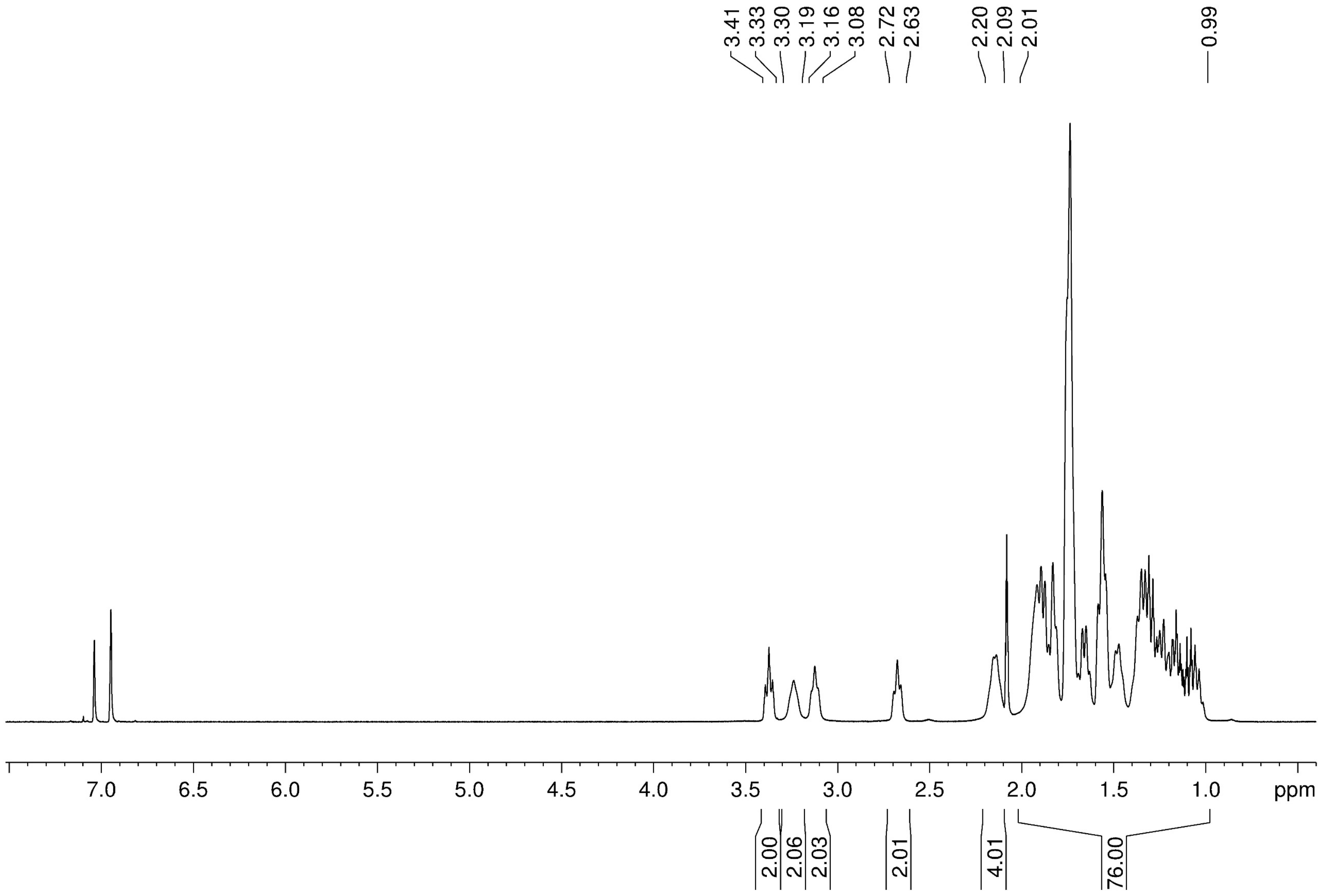

Supplementary Fig. S21 | $^{1}H\{^{11}B\}$ NMR (600.1 MHz) spectrum of **5F** in d_8-toluene at 383 K.

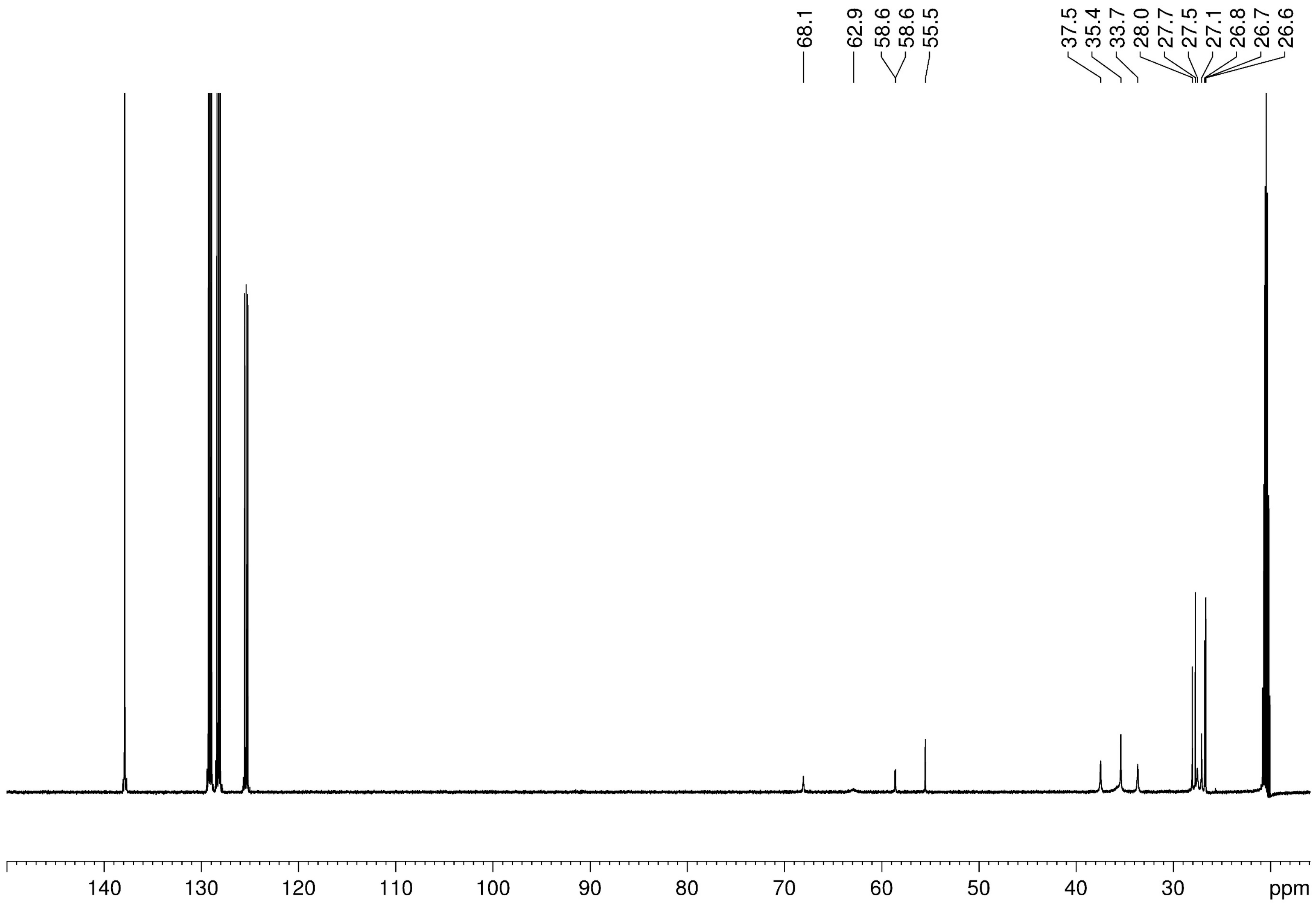

Supplementary Fig. S22 | $^{13}C\{^{1}H\}$ NMR (150.9 MHz) spectrum of **5F** in d_8-toluene at 383 K.

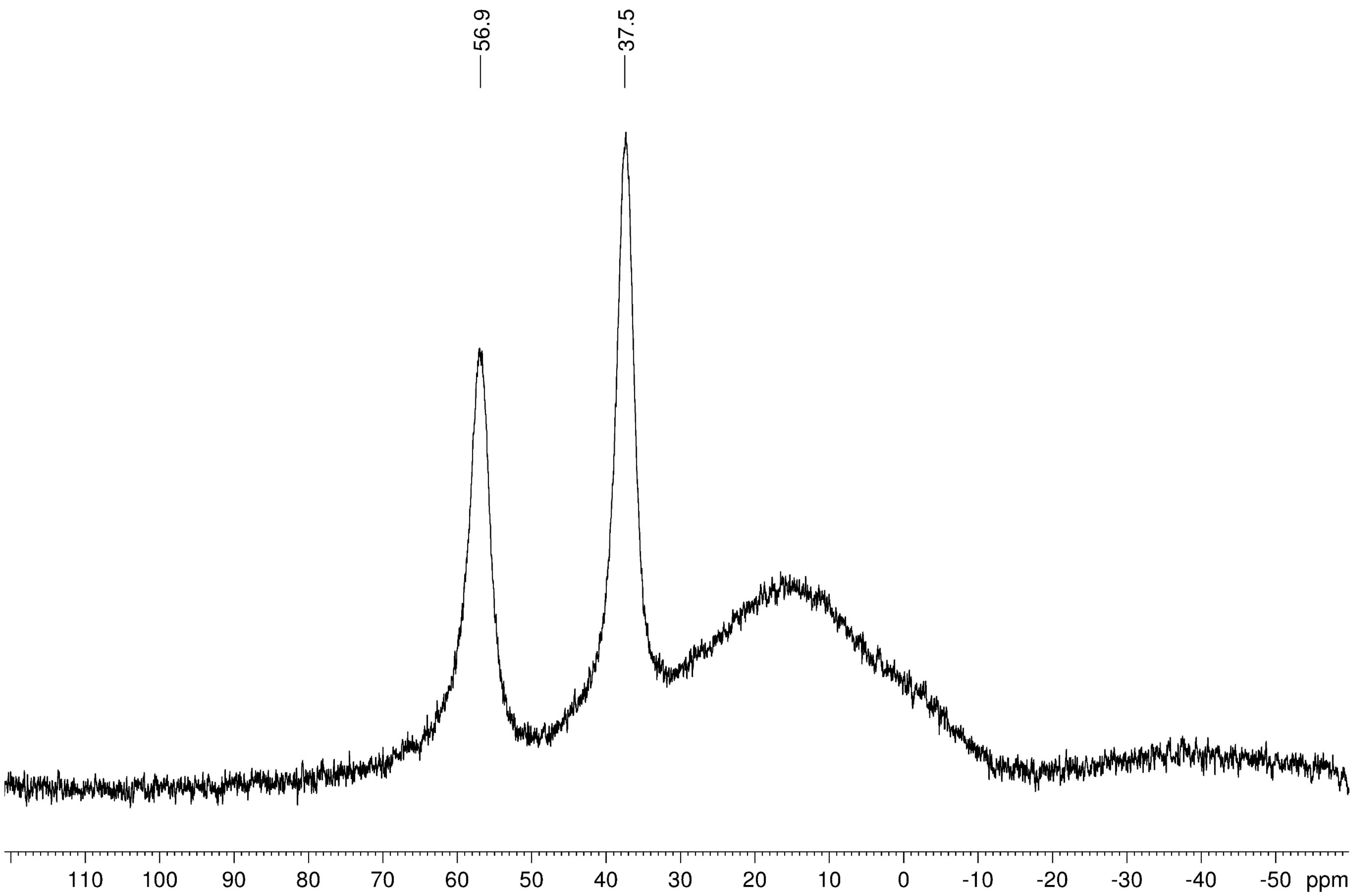

Supplementary Fig. S23 | ^{11}B NMR (192.6 MHz) spectrum of **5F** in d_8-toluene at 383 K.

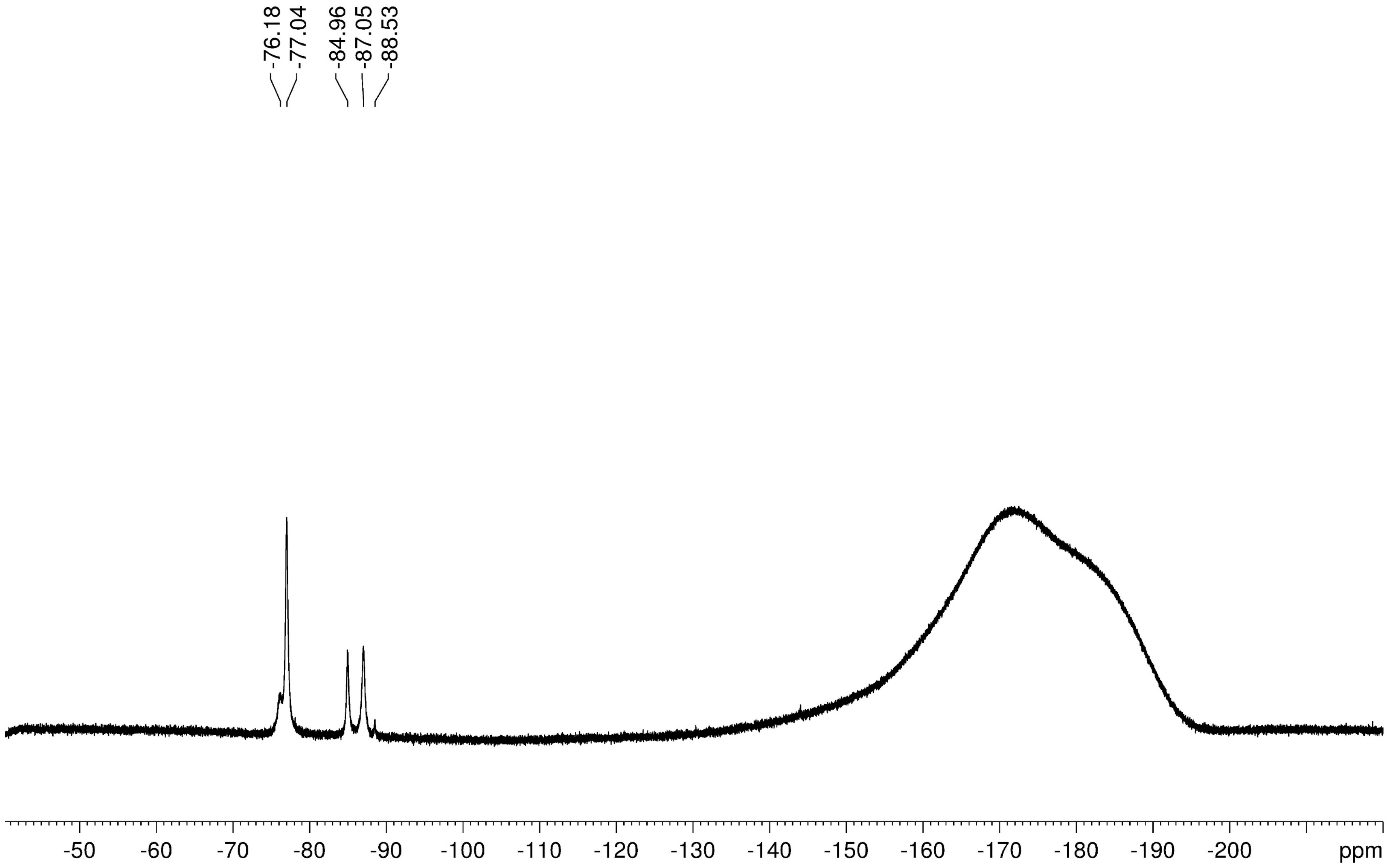

Supplementary Fig. S24 | ^{19}F NMR (564.7 MHz) spectrum of **5F** in d_8-toluene at 298 K.

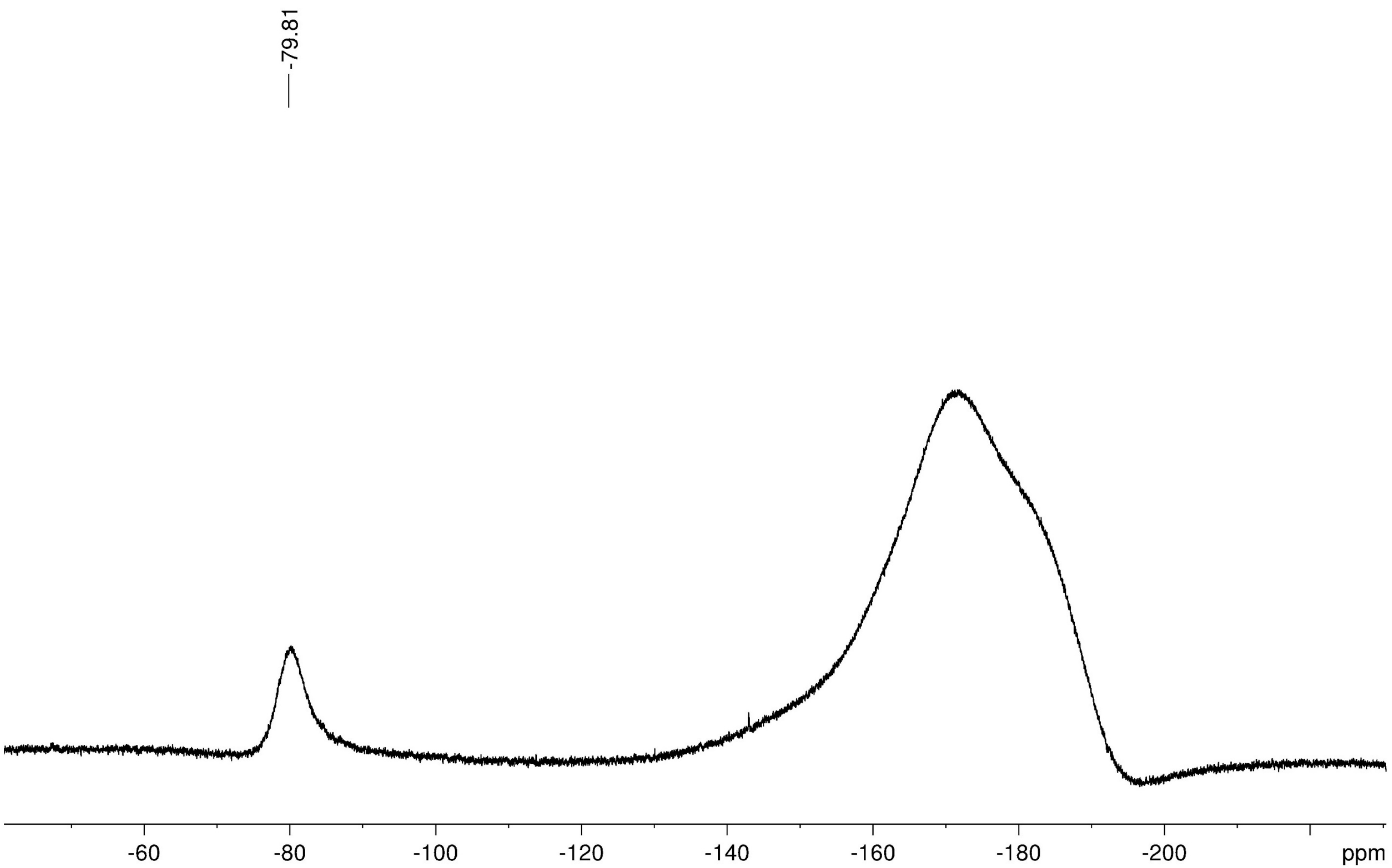

Supplementary Fig. S25 | ^{19}F NMR (564.7 MHz) spectrum of **5F** in d_8-toluene at 383 K.

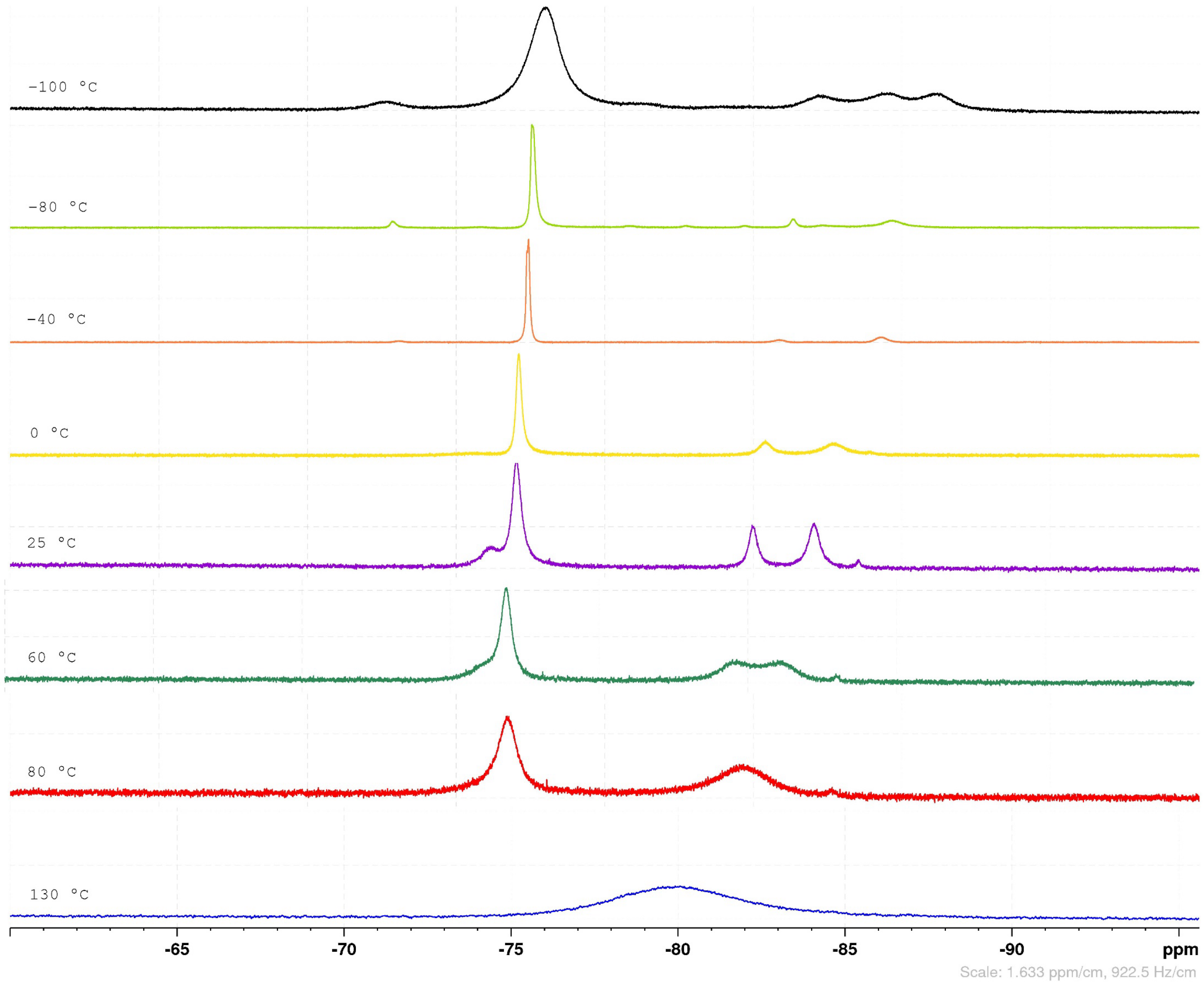

Supplementary Fig. S26 | ^{19}F NMR (564.7 MHz) spectra of **5F** in d_8-toluene from low to high temperatures.

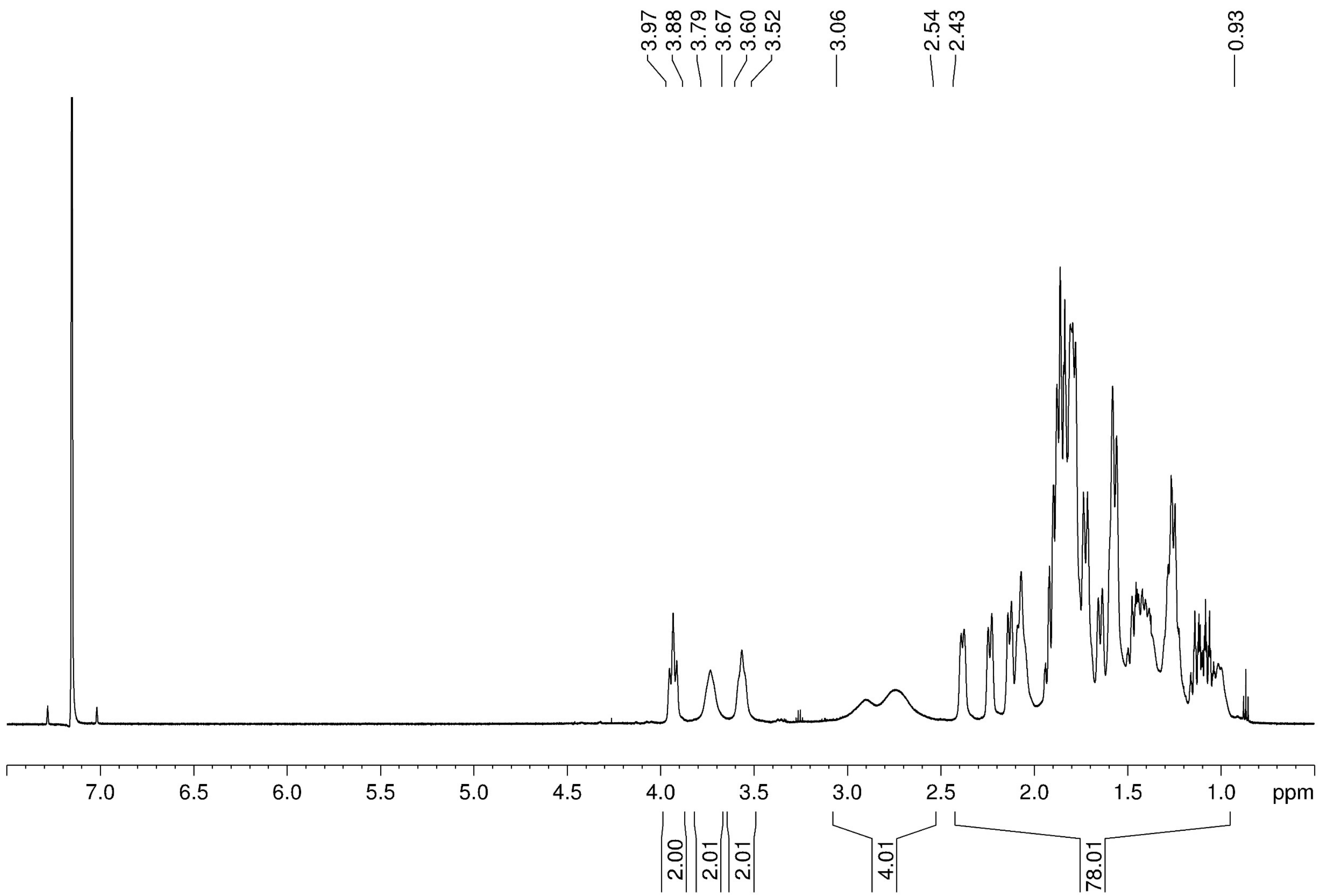

Supplementary Fig. S27 | $^{1}H\{^{11}B\}$ NMR (600.1 MHz) spectrum of **5Cl** in C_6D_6.

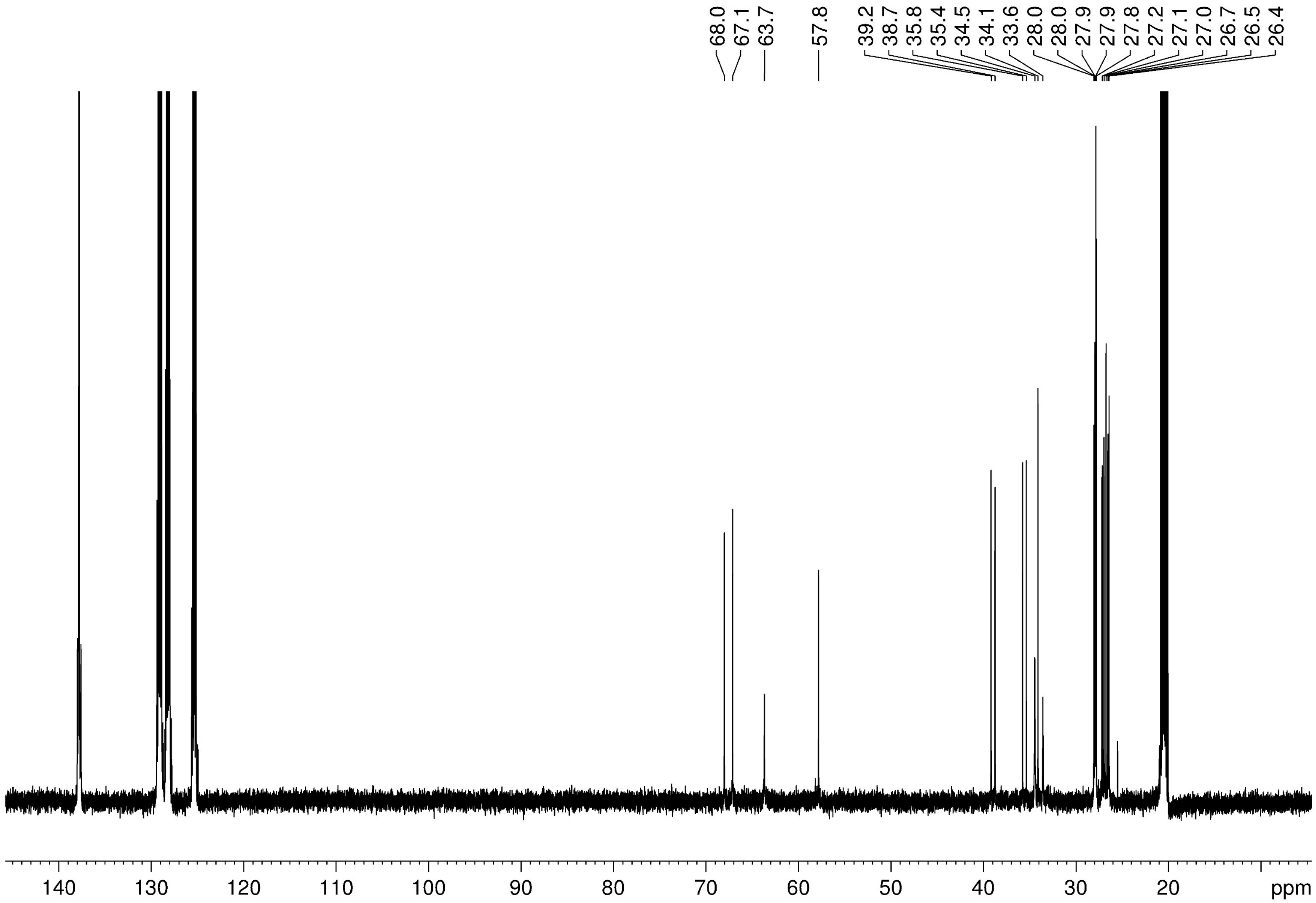

Supplementary Fig. S28 | $^{13}C\{^1H\}$ NMR (150.9 MHz) spectrum of **5Cl** in d_8-toluene at 383 K.

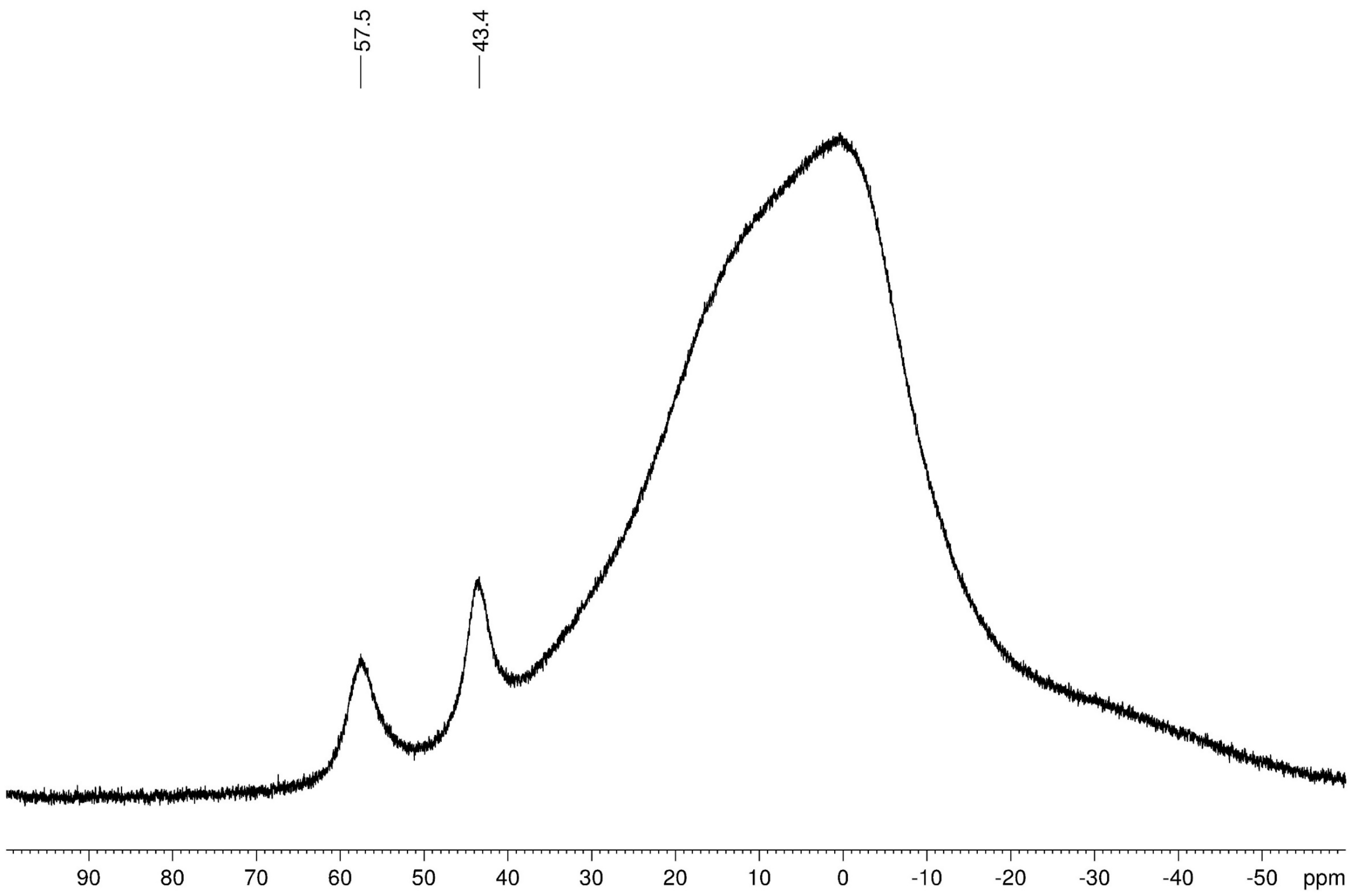

Supplementary Fig. S29 | ^{11}B NMR (192.6 MHz) spectrum of **5Cl** in d_8-toluene at 383 K.

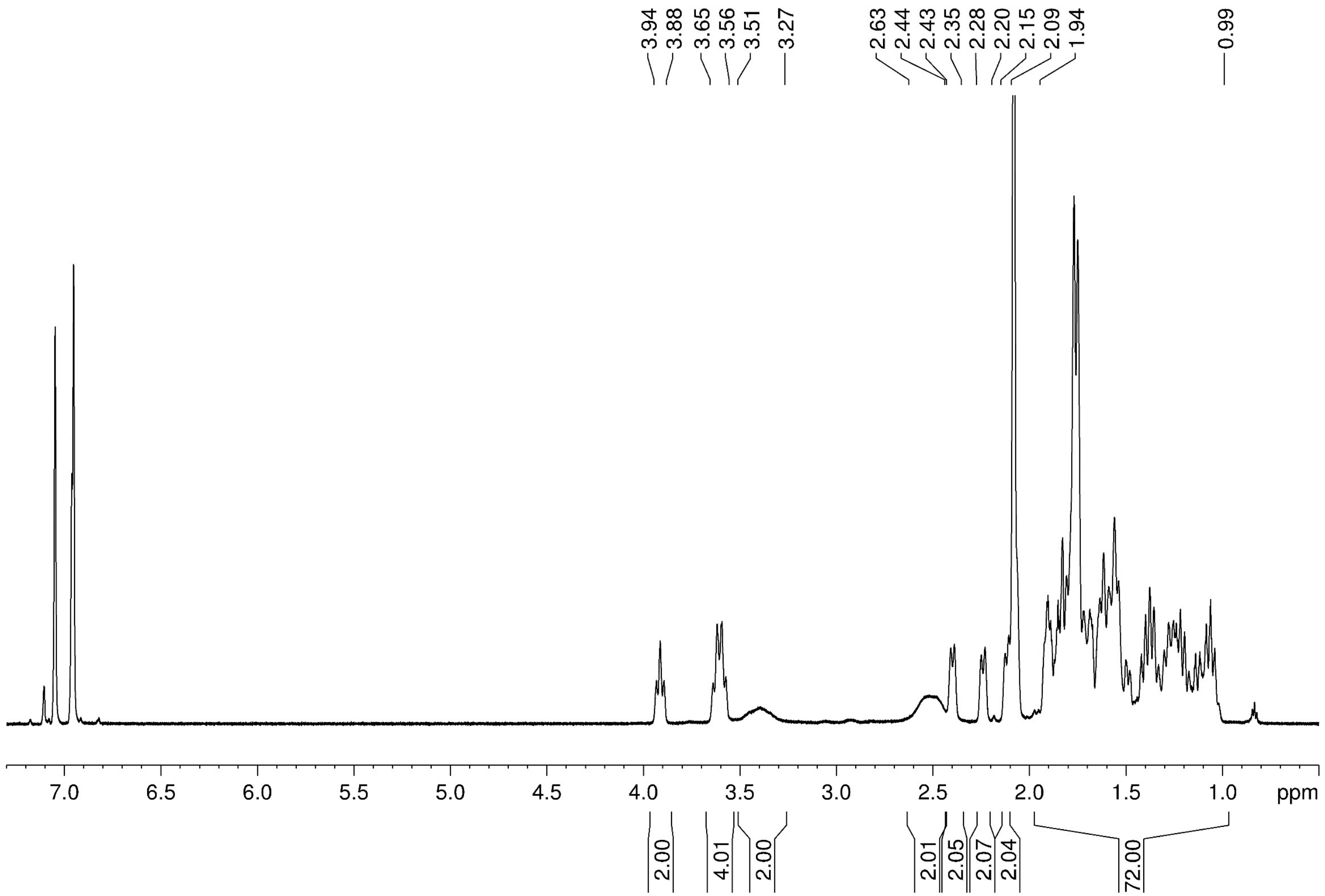

Supplementary Fig. S30 | $^{1}H\{^{11}B\}$ NMR (600.1 MHz) spectrum of **5Br** in d_8-toluene at 383 K.

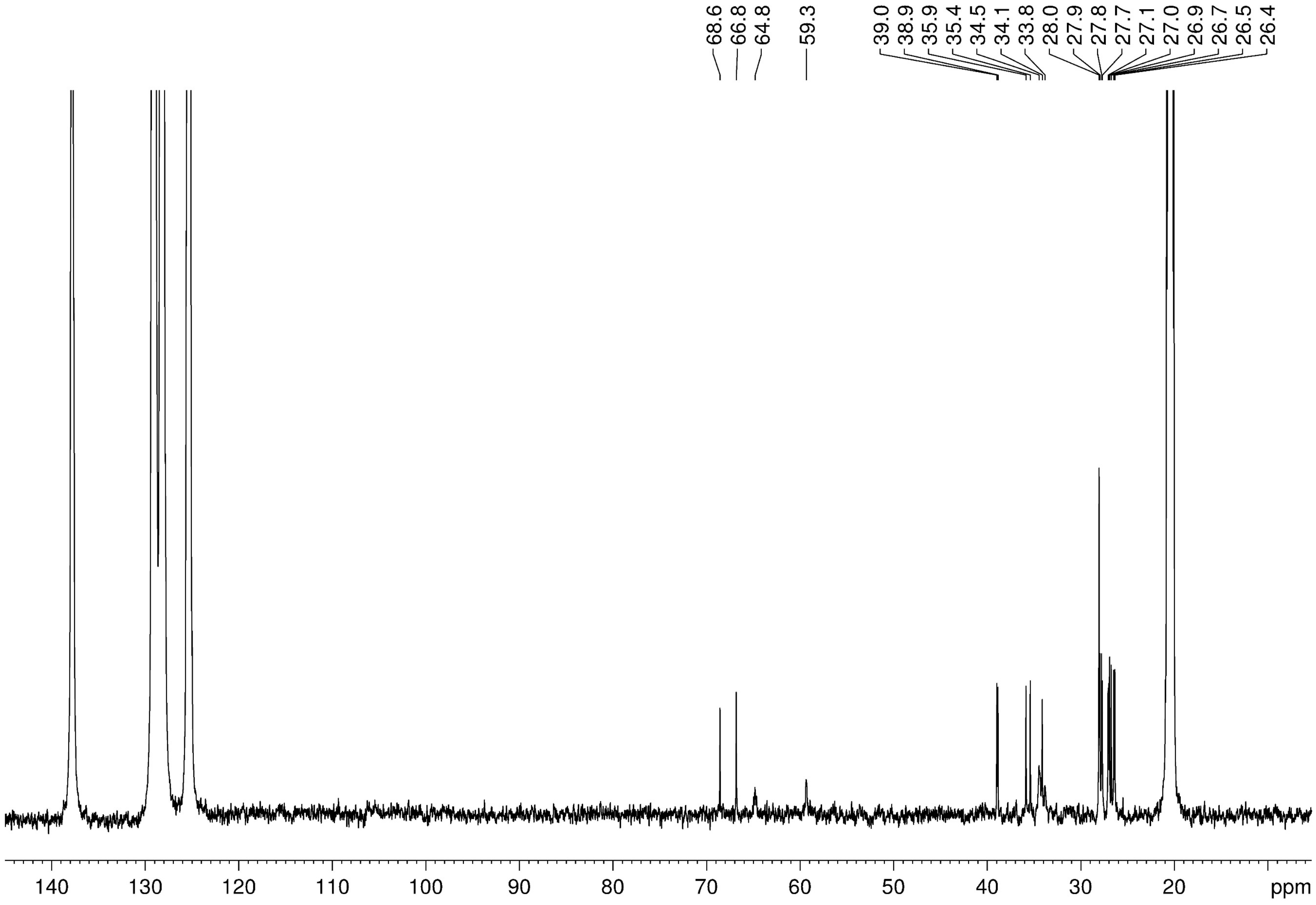

Supplementary Fig. S31 | $^{13}C\{^{1}H\}$ NMR (150.9 MHz) spectrum of **5Br** in d_8-toluene at 383 K.

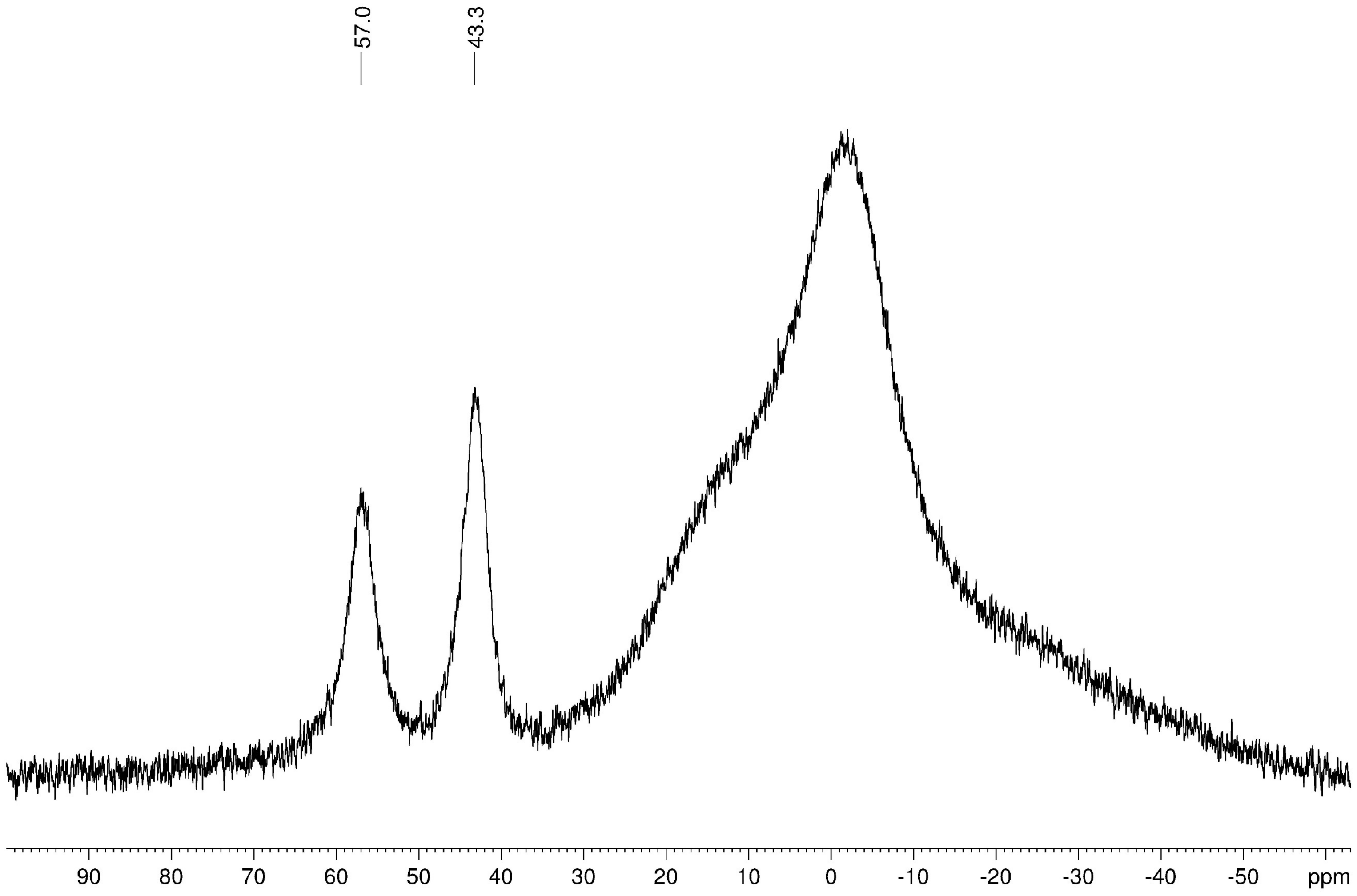

Supplementary Fig. S32 | ^{11}B NMR (192.6 MHz) spectrum of **5Br** in d_8-toluene at 383 K.

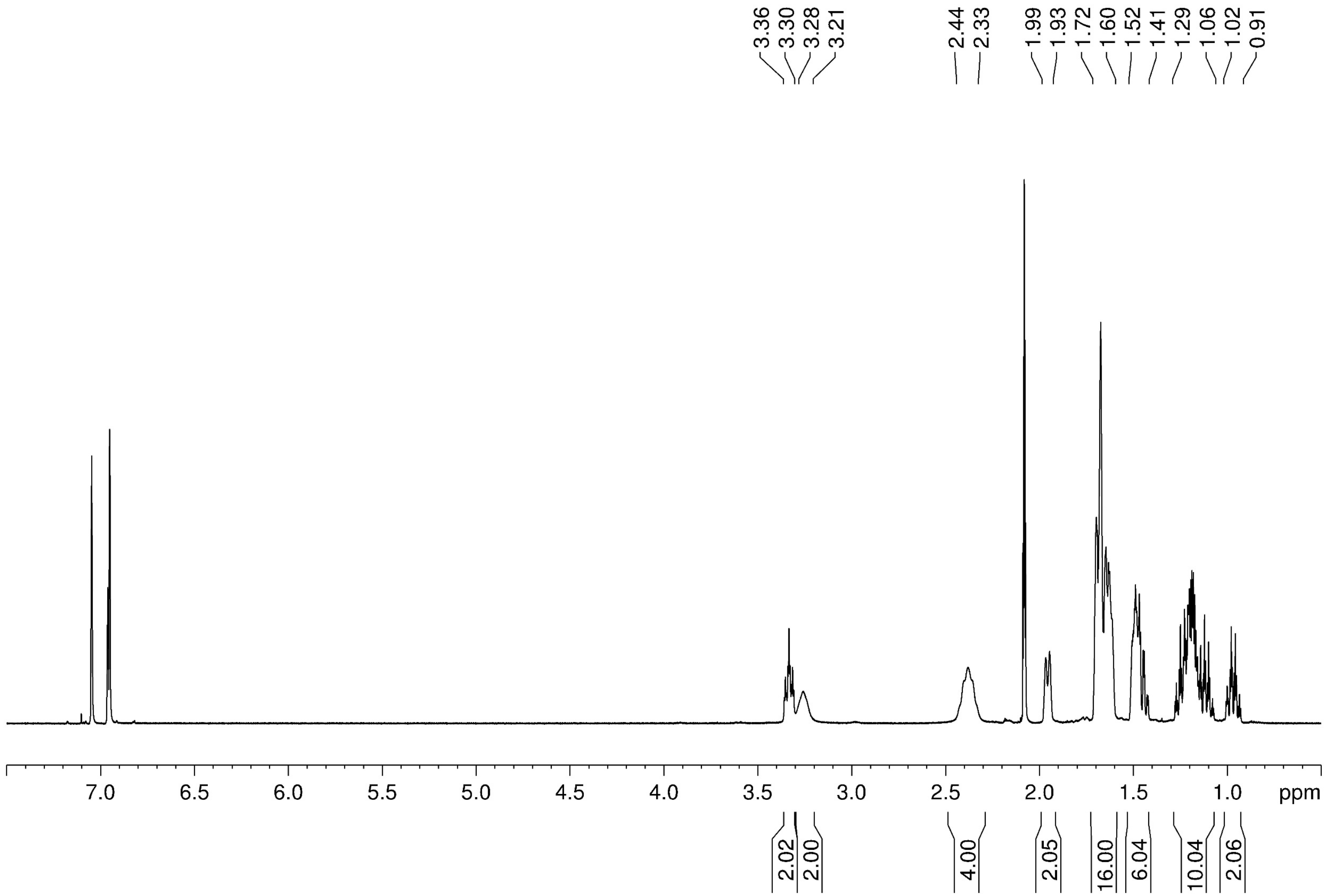

Supplementary Fig. S33 | $^{1}H\{^{11}B\}$ NMR (600.1 MHz) spectrum of **6** in d_8-toluene at 383 K.

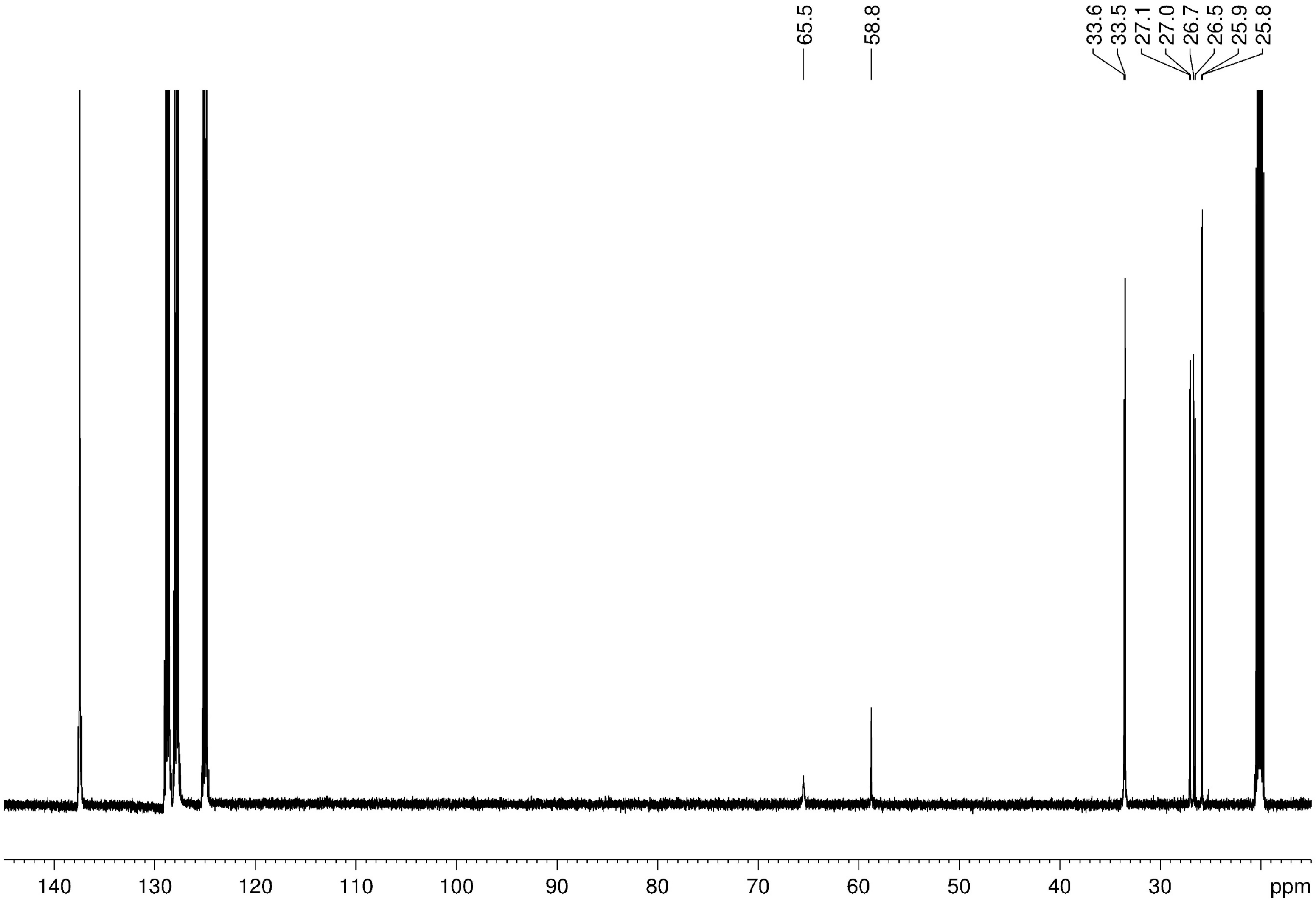

Supplementary Fig. S34 | $^{13}C\{^{1}H\}$ NMR (150.9 MHz) spectrum of **6** in d_8-toluene at 383 K.

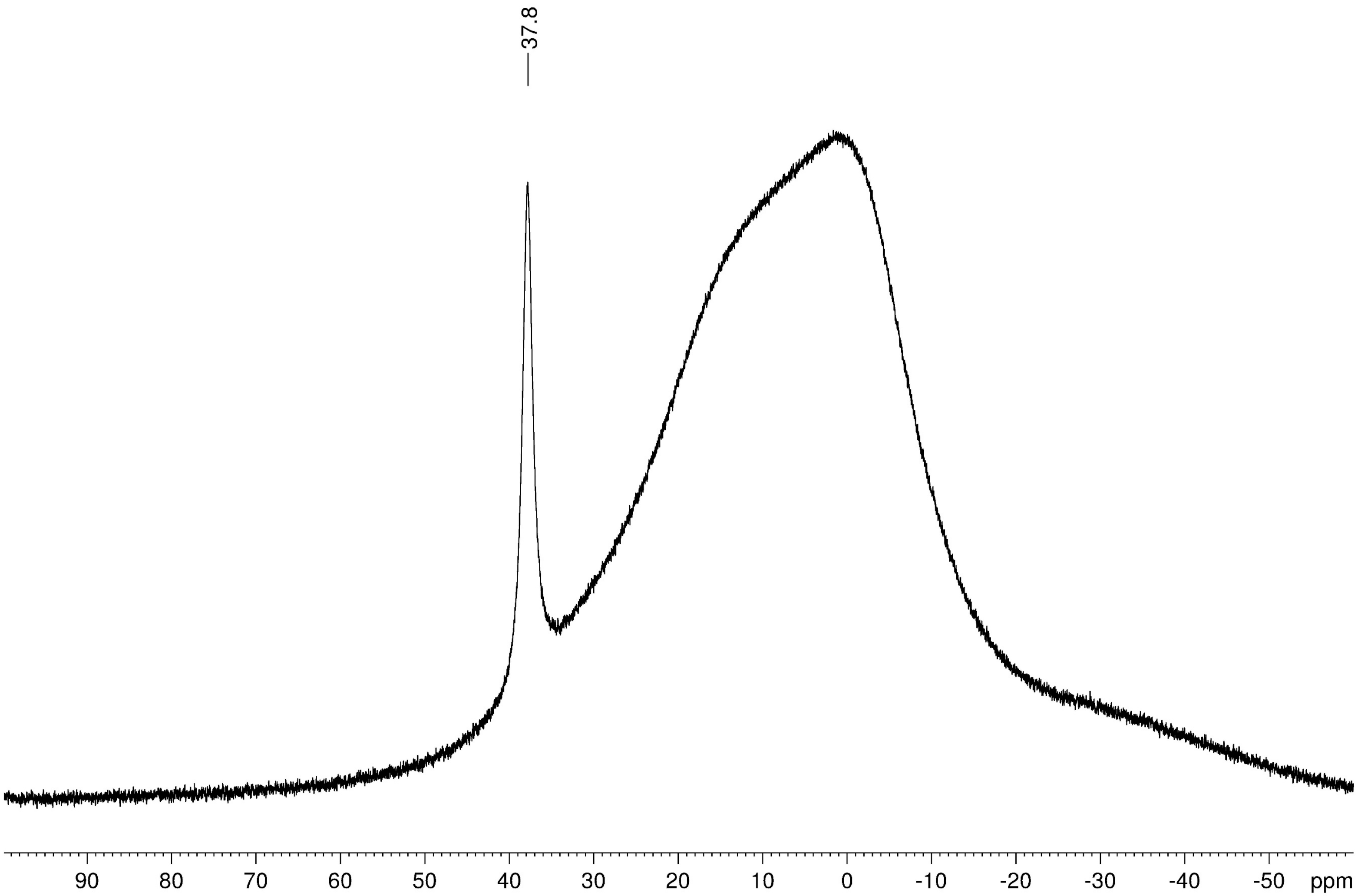

Supplementary Fig. S35 | ^{11}B NMR (192.6 MHz) spectrum of **6** in d_8-toluene at 383 K.

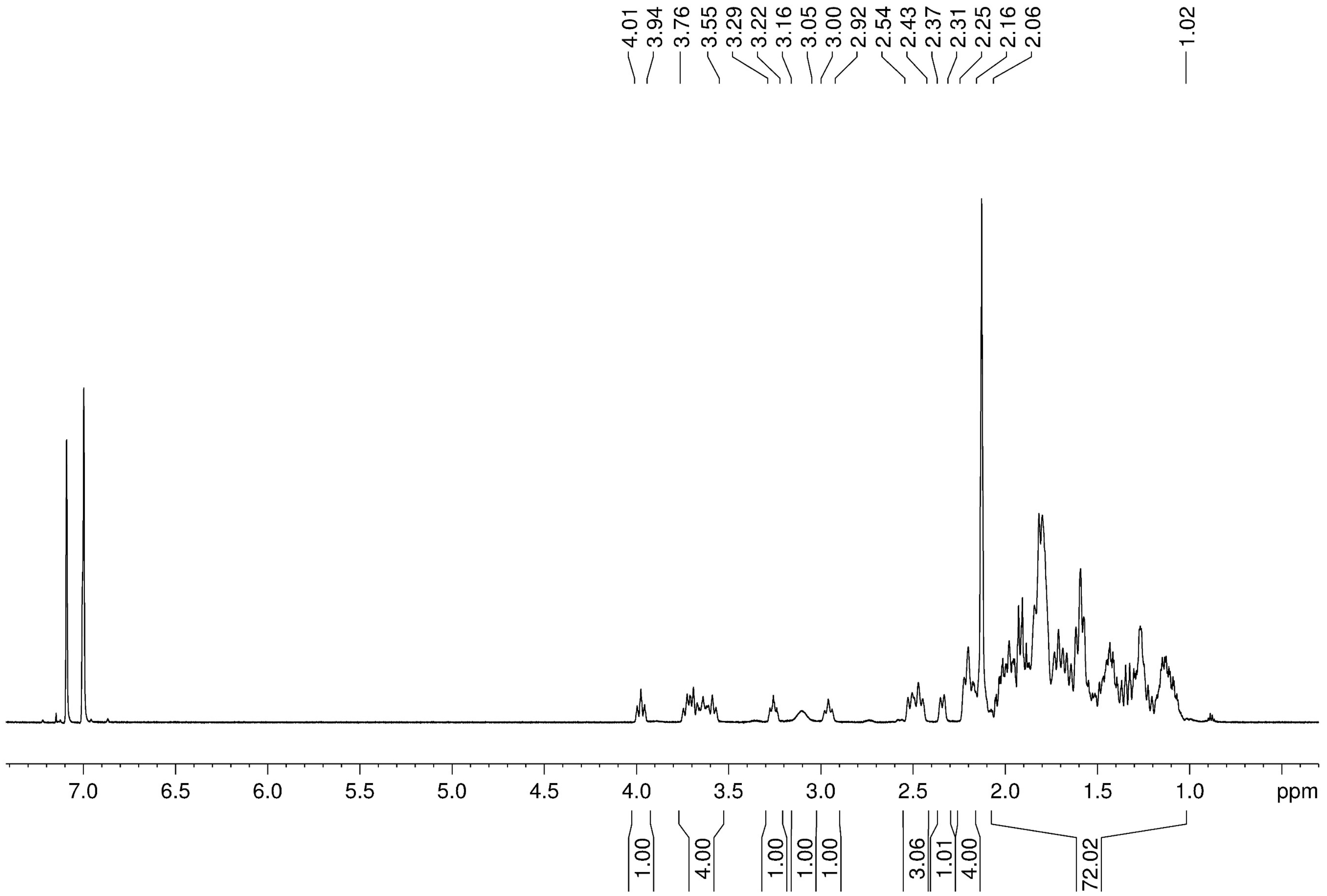

Supplementary Fig. S36 | $^{1}H\{^{11}B\}$ NMR (600.1 MHz) spectrum of **5OTf** in d_8-toluene at 393 K.

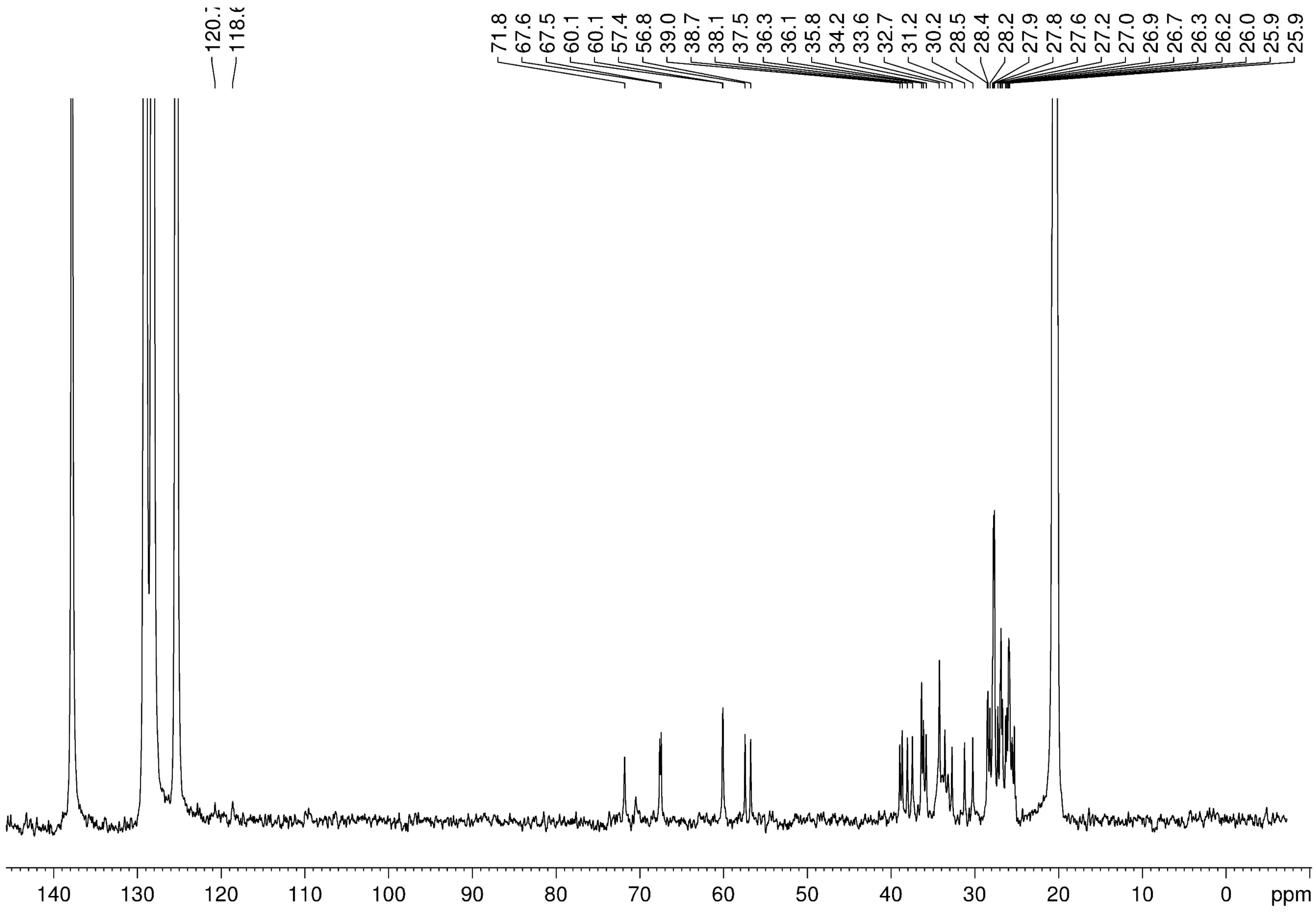

Supplementary Fig. S37 | $^{13}C\{^{1}H\}$ NMR (150.9 MHz) spectrum of **5OTf** in d_8-toluene at 383 K.

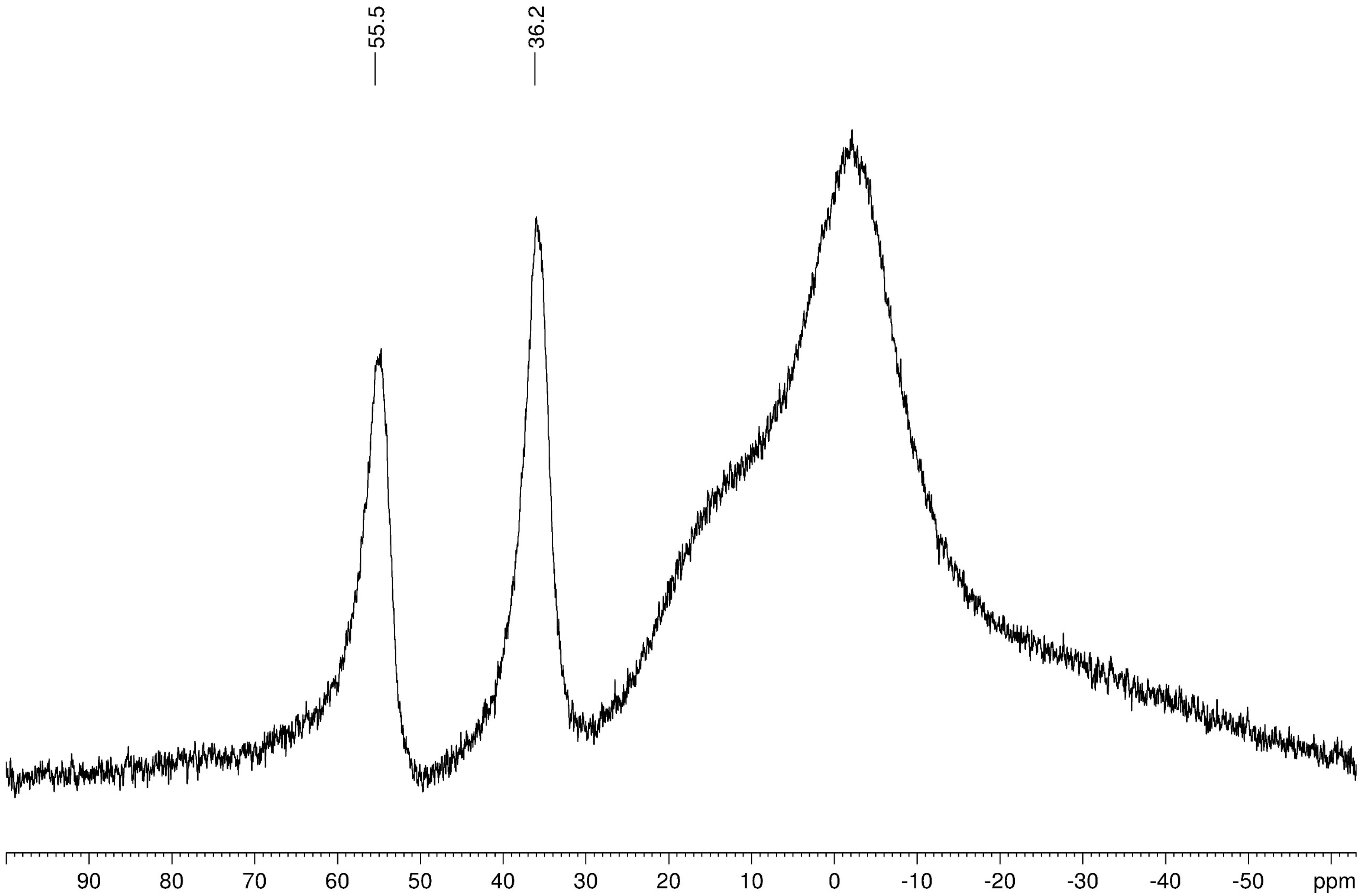

Supplementary Fig. S38 | ^{11}B NMR (192.6 MHz) spectrum of **5OTf** in d_8-toluene at 393 K.

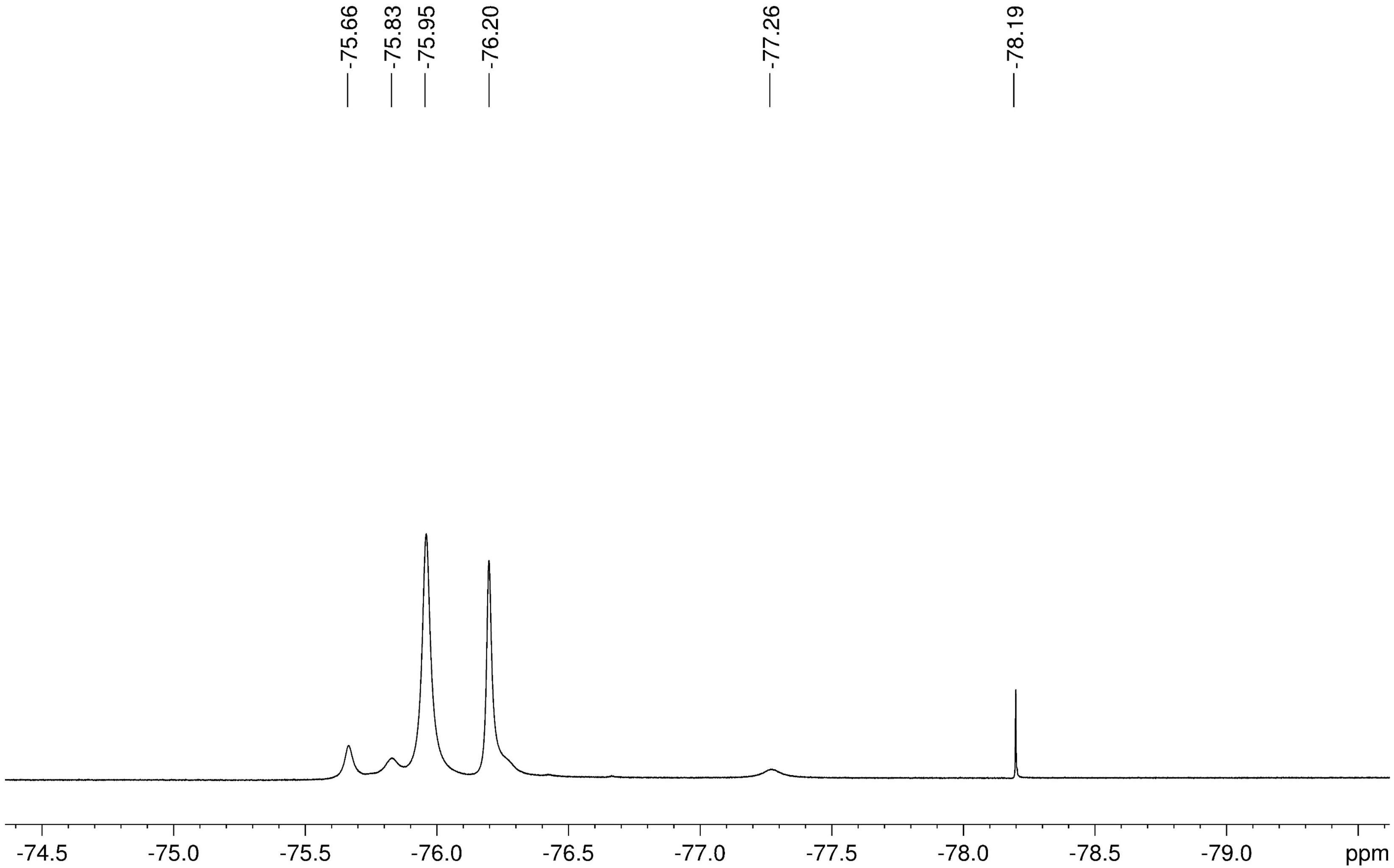

Supplementary Fig. S39 | ^{19}F NMR (564.7 MHz) spectrum of **5OTf** in d_8-toluene at 297 K.

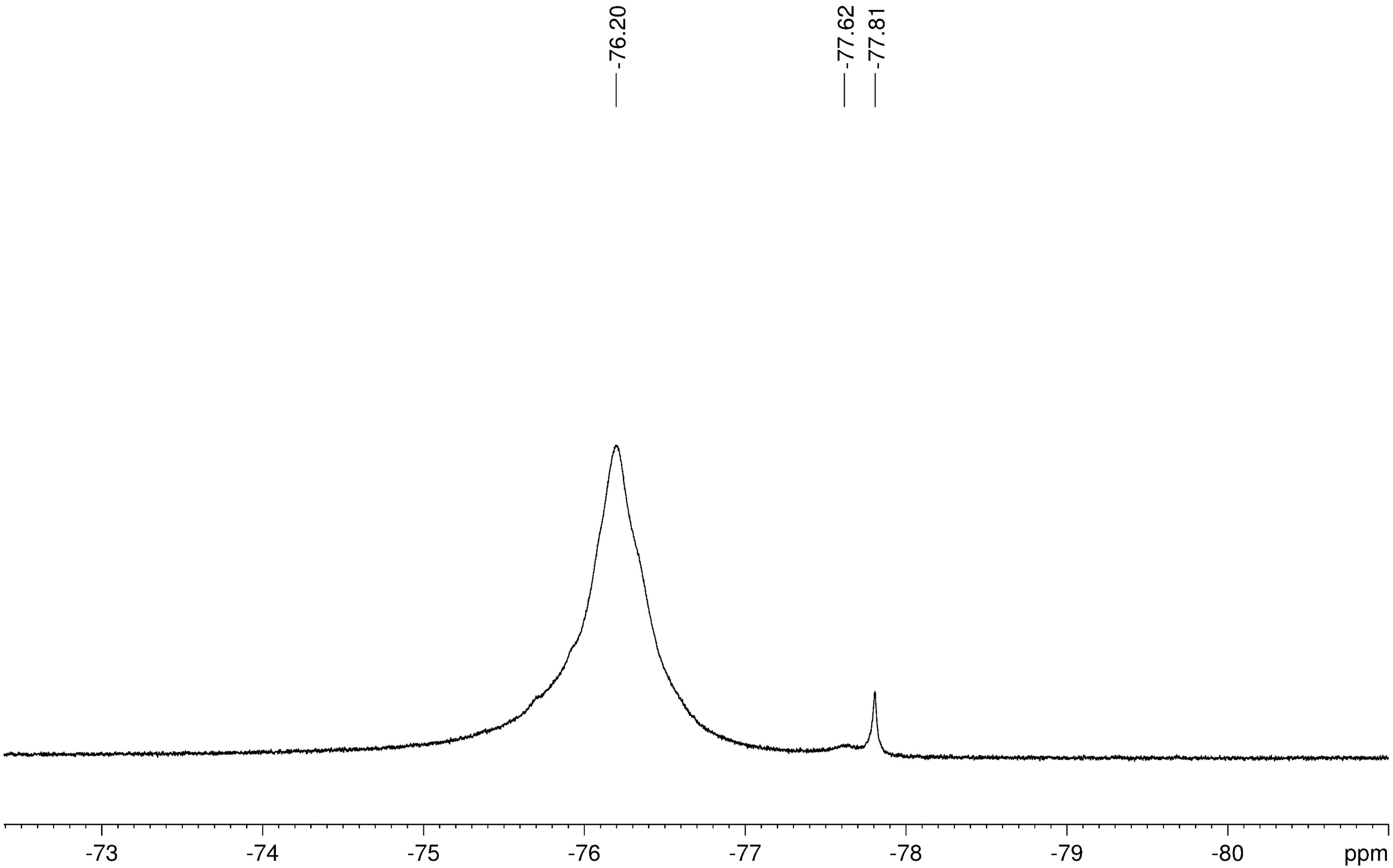

Supplementary Fig. S40 | ^{19}F NMR (564.7 MHz) spectrum of **5OTf** in d_8-toluene at 173 K.

Cyclic voltammetry

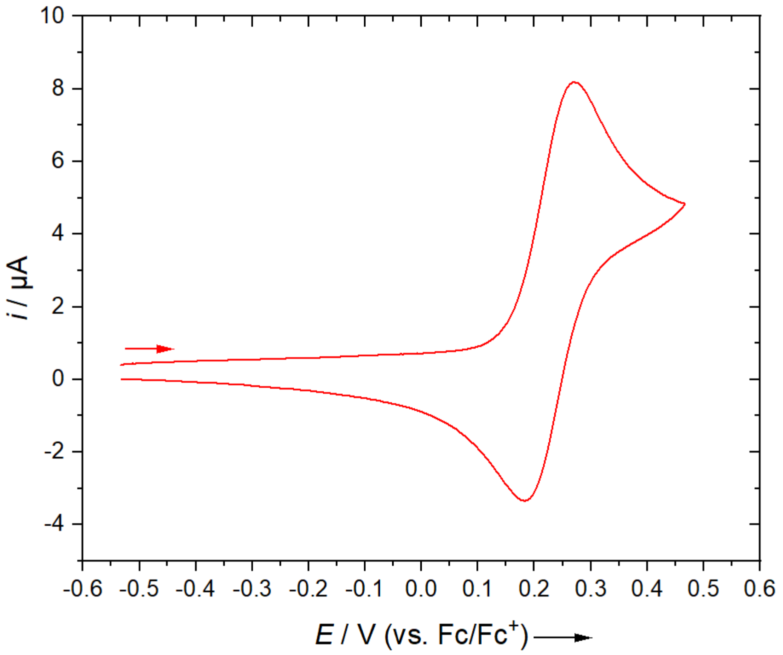

Supplementary Fig. S41 | Cyclic voltammogram of **2S** in THF/0.1 M [*n*Bu_4N][PF_6] measured at 250 mV s^{-1}. Formal potential: $E_{1/2}$ = 0.23 V [3].

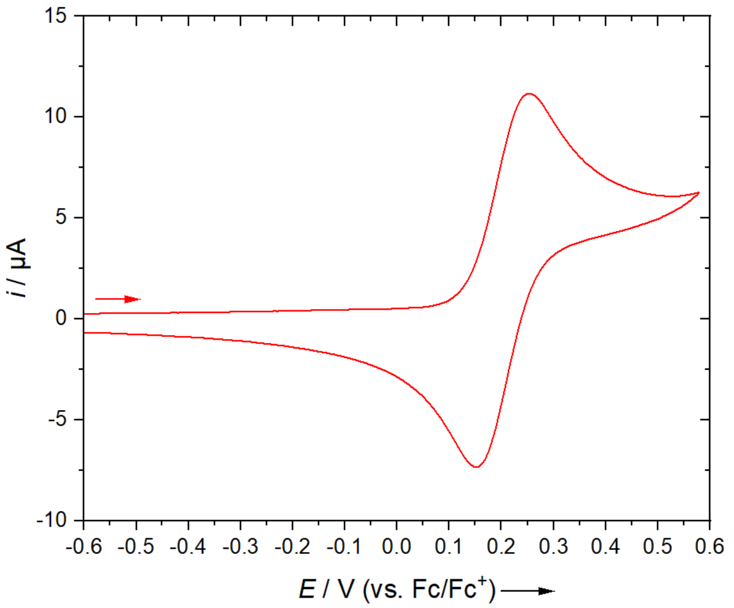

Supplementary Fig. S42 | Cyclic voltammogram of **2Se** in THF/0.1 M [*n*Bu_4N][PF_6] measured at 250 mV s^{-1}. Formal potential: $E_{1/2}$ = 0.20 V [3].

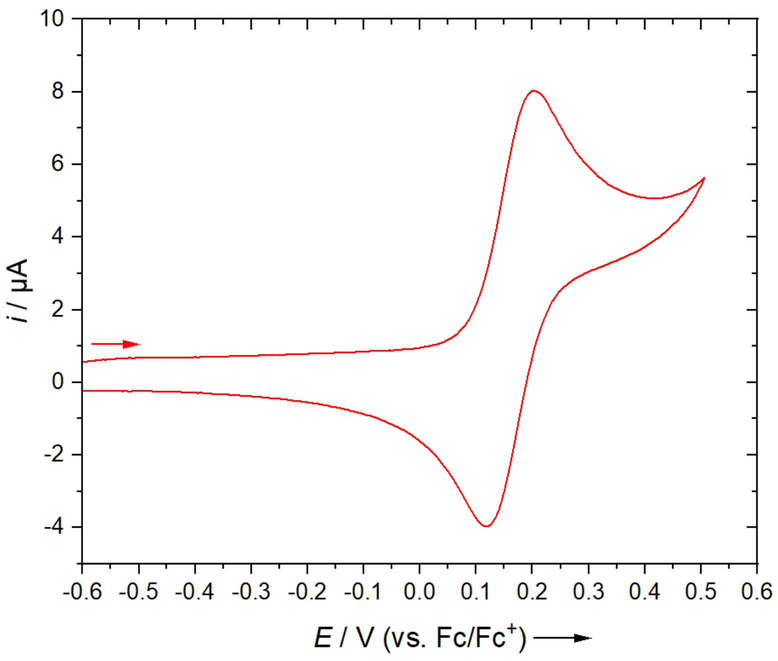

Supplementary Fig. S43 | Cyclic voltammogram of **2Te** in THF/0.1 M [nBu$_4$N][PF$_6$] measured at 250 mV s^{-1}. Formal potential: $E_{1/2}$ = 0.16 V [3].

UV-vis spectrum

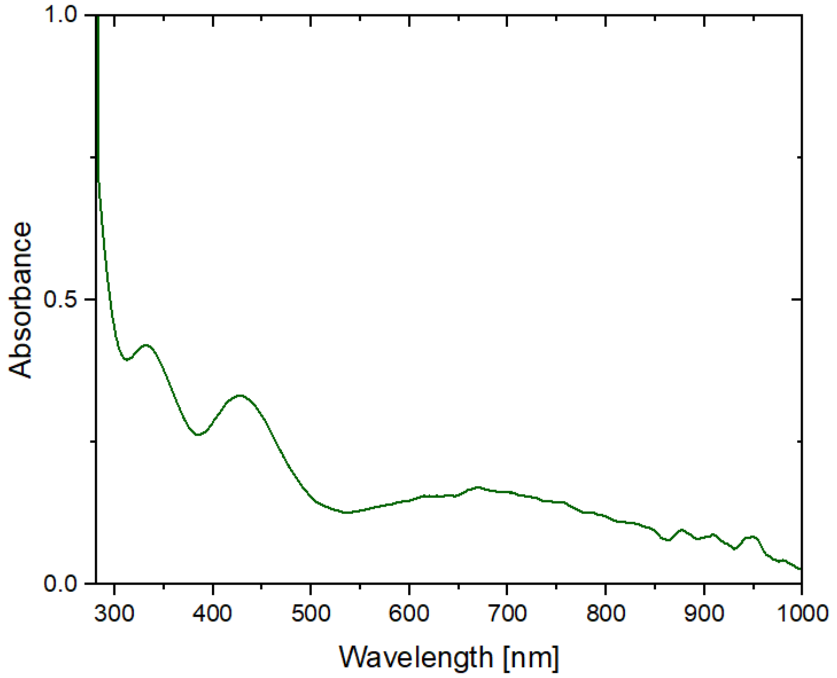

Supplementary Fig. S44 | UV-vis absorption spectrum of [**2S**][Al(OC(CF_3)$_3$)$_4$] in diethyl ether at 23 °C (λ_1 = 333 nm, λ_2 = 428 nm, and λ_3 = 700 nm (shoulder)). Weak absorption bands at higher wavelengths are likely due to impurities.

EPR spectrum

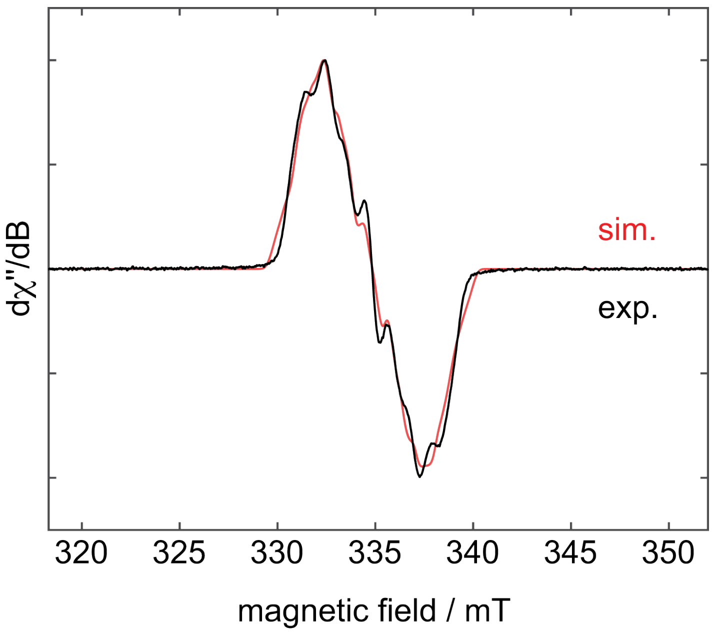

Supplementary Fig. S45 | Experimental (black) and simulated (red) continuous-wave (CW) X-band EPR spectra of the radical cation [**2S**][$Al(OC(CF_3)_3)_4$] in a mixture of toluene and 1,2-difluorobenzene at room temperature. The signal is centered at a *g*-value of 2.0022. Due to the low spectral resolution, reliable determination of the boron and nitrogen hyperfine coupling constants via spectral simulation proved challenging. A symmetric spin delocalization model involving two sets of equivalent boron atoms clearly fails to reproduce the experimental spectrum. In contrast, an unsymmetric spin distribution, as suggested by DFT calculations, produces a markedly improved fit. Yet some nuanced spectral features remain only partially accounted for, even when nitrogen couplings are included. The best-fit simulation parameters include couplings to ^{11}B nuclei of 37, 18, 17, and 12 MHz. Experimental parameters: microwave frequency = 9.38 GHz; microwave power = 1 mW; modulation amplitude = 0.5 G; conversion time = 20 ms; modulation frequency = 100 kHz.

Single-crystal X-ray diffraction data

The crystallographic data of **2S**, **2Se**, **2Te**, **$[Cy_2NBO]_3$**, **$[2S][Al(OC(CF_3)_3)_4]$**, **4S**, **5F**, **5Cl**, **5Br**, and **6** were collected on a XtaLAB Synergy Dualflex HyPix diffractometer with a Hybrid Pixel array detector and multi-layer mirror monochromated $Cu_{K\alpha}$ or $Mo_{K\alpha}$ radiation. The structures were solved using the intrinsic phasing method[4], refined with the ShelXL program[5] and expanded using Fourier techniques. All non-hydrogen atoms were refined anisotropically. Hydrogen atoms were included in structure factor calculations. All hydrogen atoms were assigned to idealized geometric positions.

Crystallographic data have been deposited with the Cambridge Crystallographic Data Center as supplementary publication numbers CCDC 2423870-2423879. These data can be obtained free of charge from The Cambridge Crystallographic Data Center *via* www.ccdc.cam.ac.uk/data_request/cif.

Refinement details for 2S: Two NCy_2 moieties showed disorder. The atomic displacement parameters (ADPs) of overlapping atoms from different PARTs (N1 of RESIs 4/14 and 7/17; C1 to C6 of RESIs 5/15, 6/16, 8/18 and 9/19) were restrained using the similarity restraint (SIMU) and rigid body restraint (RIGU). The 1-2 and 1-3 distances of the disordered cyclohexyl moieties were restrained using the same distance restraint (SAME). The N1_4/14-C1_5/15/6/16, B2_1-N1_4/14, C1_5/6-C1_15/16, N1_7/17-C1_8/18/9/19, B3_1-N1_7/17, C1_8/9-C1_18/19 distances of the disordered cyclohexyl moieties were restrained using the same distance restraint (SADI).

Crystal data for 2S: $C_{48}H_{88}B_4N_4S$, M_r = 796.52, clear colorless block, 0.120×0.080×0.030 mm^3, triclinic space group $P\bar{1}$, a = 10.6721(2) Å, b = 10.87370(10) Å, c = 22.1467(3) Å, α = 103.8420(10)°, β = 100.6280(10)°, γ = 91.4680(10)°, V = 2446.02(6) Å^3, Z = 2, ρ_{calcd} = 1.081 g·cm^{-3}, μ = 0.834 mm^{-1}, F(000) = 880, T = 100(2) K, R_1 = 0.0430, wR_2 = 0.0961, 8892 independent reflections [2θ≤136.49°] and 750 parameters.

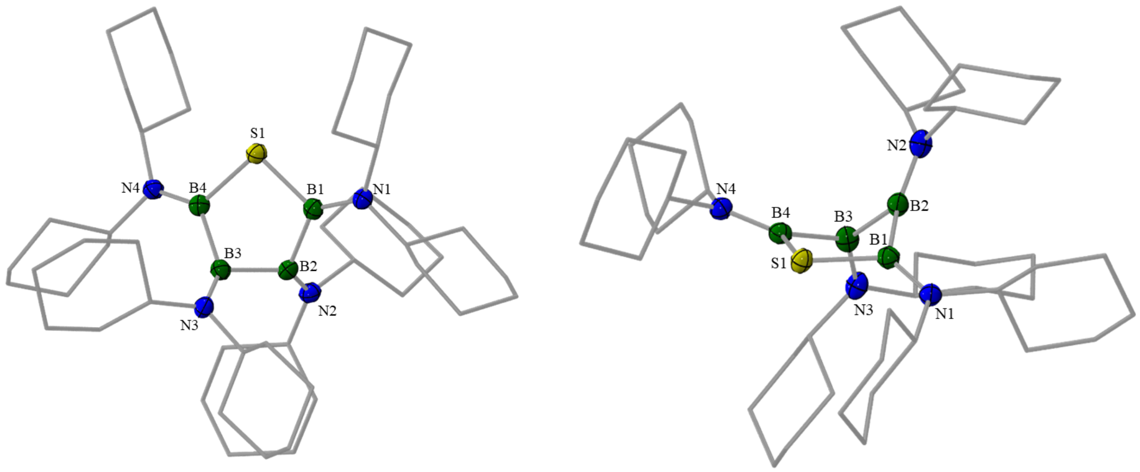

Supplementary Fig. S46 | Solid-state structure of **2S** viewed from two different perspectives. Atomic displacement ellipsoids represented at 50%. Ellipsoids at the ligand periphery and hydrogen atoms have been omitted for clarity.

Refinement details for 2Se: One NCy_2 moiety showed disorder. The atomic displacement parameters (ADPs) of overlapping atoms from different PARTs (N7 of RESIs 13/113; C1 to C6 of RESIs 14/114 and 15/115) were restrained using the similarity restraint (SIMU) and the rigid body restraint (RIGU). The 1-2 and 1-3 distances of the disordered cyclohexyl moieties were restrained using the same distance restraint (SAME). The N7-C1 and the B2-N7 distances of the disordered cyclohexyl moieties were restrained using the same distance restraint (SADI). The unit cell contains solvent molecules which have been treated as a diffuse contribution to the overall scattering without specific atom positions by SQUEEZE/PLATON. 1.5 equivalents of hexane were squeezed.

Crystal data for **2Se**: $C_{48}H_{88}B_4N_4Se$, M_r = 843.42, colorless plate, 0.130×0.110×0.030 mm^3, triclinic space group $P\bar{1}$, a = 12.5382(2) Å, b = 18.5764(3) Å, c = 26.2673(4) Å, α = 100.5810(10)°, β = 98.8540(10)°, γ = 104.3420(10)°, V = 5697.43(16) $Å^3$, Z = 4, ρ_{calcd} = 0.983 $g \cdot cm^{-3}$, μ = 1.104 mm^{-1}, $F(000)$ = 1832, T = 100(2) K, R_1 = 0.0501, wR_2 = 0.0880, 20674 independent reflections [$2\theta \leq 136.492°$] and 1145 parameters.

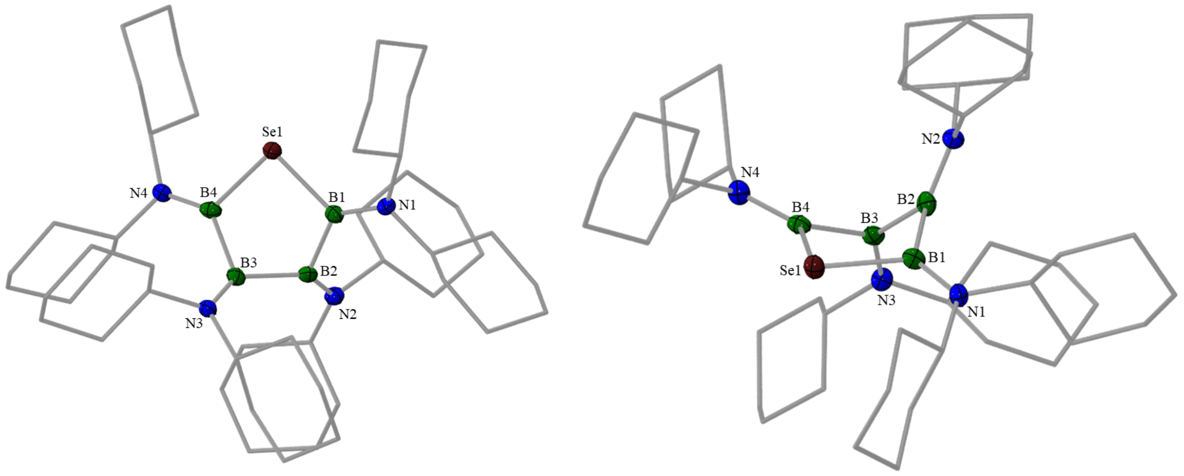

Supplementary Fig. S47 | Solid-state structure of **2Se** shown in two different views. Atomic displacement ellipsoids represented at 50%. Ellipsoids of the ligand periphery and hydrogen atoms have been omitted for clarity.

Crystal data for **2Te**: $C_{48}H_{88}B_4N_4Te$, M_r = 892.06, clear colorless block, 0.220×0.130×0.060 mm^3, monoclinic space group $P2_1/c$, a = 10.77820(10) Å, b = 41.8253(3) Å, c = 10.95180(10) Å, β = 92.7550(10)°, V = 4931.38(7) Å^3, Z = 4, ρ_{calcd} = 1.202 g·cm^{-3}, μ = 5.007 mm^{-1}, F(000) = 1904, T = 100(2) K, R_1 = 0.0245, wR_2 = 0.0598, 9714 independent reflections [$2\theta \leq 144.254°$] and 514 parameters.

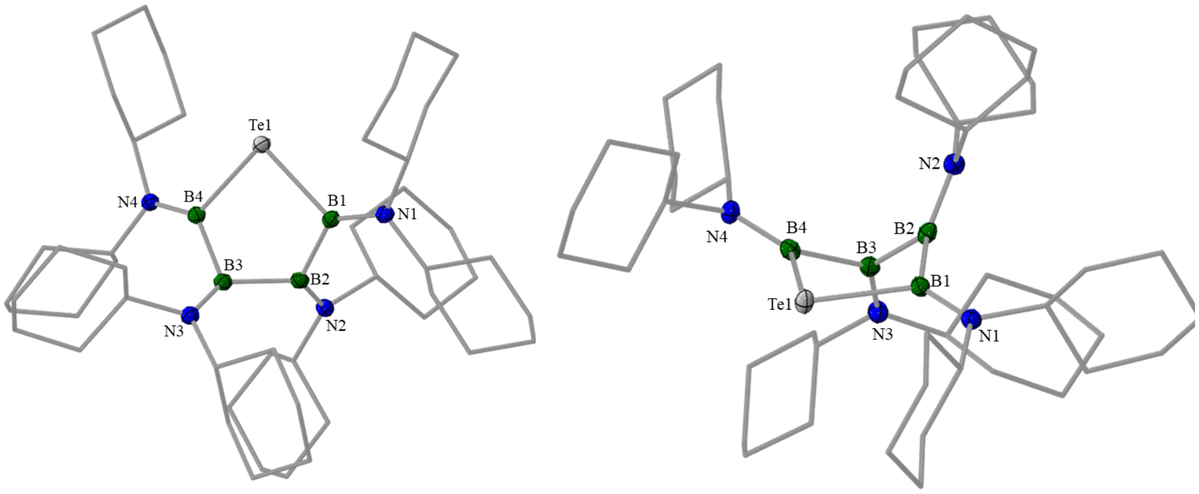

Supplementary Fig. S48 | Solid-state structure of **2Te** shown from two different perspectives. Atomic displacement ellipsoids represented at 50%. Ellipsoids of ligand periphery and hydrogen atoms omitted for clarity.

Refinement details for 3S: Refined as a two-component perfect inversion twin.

Crystal data for **3S**: $C_{24}H_{44}B_2N_2S_2$, M_r = 446.35, colorless block, 0.170×0.160×0.080 mm^3, orthorhombic space group $P2_12_12$, a = 11.3575(3) Å, b = 10.6505(3) Å, c = 10.6174(3) Å, V = 1284.31(6) Å^3, Z = 2, r_{calcd} = 1.154 g·cm^{-3}, m = 1.955 mm^{-1}, F(000) = 488, T = 100(2) K, R_1 = 0.0771, wR_2 = 0.1875, Flack parameter = 0.5, 2281 independent reflections [$2q \leq 133.186°$] and 137 parameters.

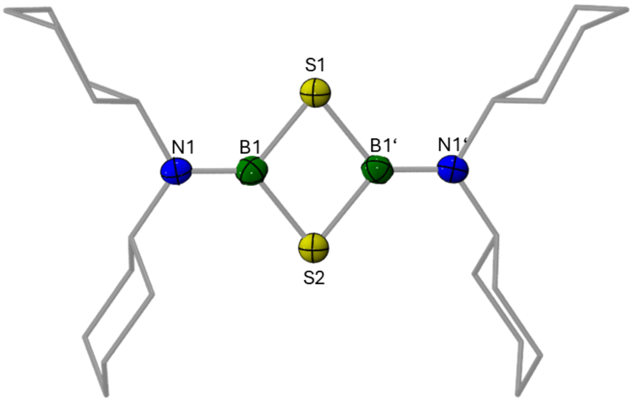

Supplementary Fig. S49 | Solid-state structure of **3S.** Atomic displacement ellipsoids represented at 50%. Ellipsoids of ligand periphery and hydrogen atoms omitted for clarity.

Refinement details for [2S][Al(OC(CF$_3$)$_3$)$_4$]: The displacement parameters of atoms F4 and F8 of the residue 17, atom F2 of the residue 18 and atom F2 of the residue 19 were constrained to the same value with the EADP keyword. The atomic displacement parameters of atoms O1, C1, C2, C3, C4 , F1, F2, F3, F4, F5, F6, F7, F8 and F9 of the residues 11, 12, 13, 14, 15, 16, 17, 18, 19 and 20 were restrained using the DELU keyword in the ShelXL input ('rigid bond' restraint for the bonds in the connectivity list). The atomic displacement parameters of atoms O1, C1, C2, C3, C4 , F1, F2, F3, F4, F5, F6, F7, F8 and F9 of the residues 11, 12, 13, 14, 15, 16, 17, 18, 19 and 20 were restrained with the RIGU keyword in the ShelXL input ('enhanced rigid bond' restraint for all bonds in the connectivity list). The displacement parameters of atoms O1, C1, C2, C3, C4 , F1, F2, F3, F4, F5, F6, F7, F8 and F9 of the residues 11, 12, 13, 14, 15, 16, 17, 18, 19 and 20 were restrained to the same value with the similarity restraint SIMU. The U_{ii} displacement parameters of atoms C1, C2, C3, C4, C5, C6 and C7 of the toluene were restrained with the ISOR keyword to approximate isotropic behavior. The 1–2 and 1–3 distances in all CCF_3 residues were restrained to the same values with SAME. The unit cell

contains solvent molecules (106 electrons found in S.A.V) which have been treated as a diffuse contribution to the overall scattering without specific atom positions by SQUEEZE/PLATON.

Crystal data for **[2S][Al(OC(CF$_3$)$_3$)$_4$]**: $C_{135}H_{184}Al_2B_8F_{72}N_8O_8S_2$, M_r = 3619.45, dark green, 0.350×0.230×0.130 mm^3, triclinic space group $P\bar{1}$, a = 15.2550(3) Å, b = 15.3814(3) Å, c = 19.3068(4) Å, α = 77.308(2)°, β = 86.739(2)°, γ = 86.987(2)°, V = 4408.63(16) Å^3, Z = 1, ρ_{calcd} = 1.363 g·cm^{-3}, μ = 1.512 mm^{-1}, F(000) = 1860, T = 100(2) K, R_1 = 0.0749, wR_2 = 0.1704, 17266 independent reflections [2θ≤150.29°] and 1881 parameters.

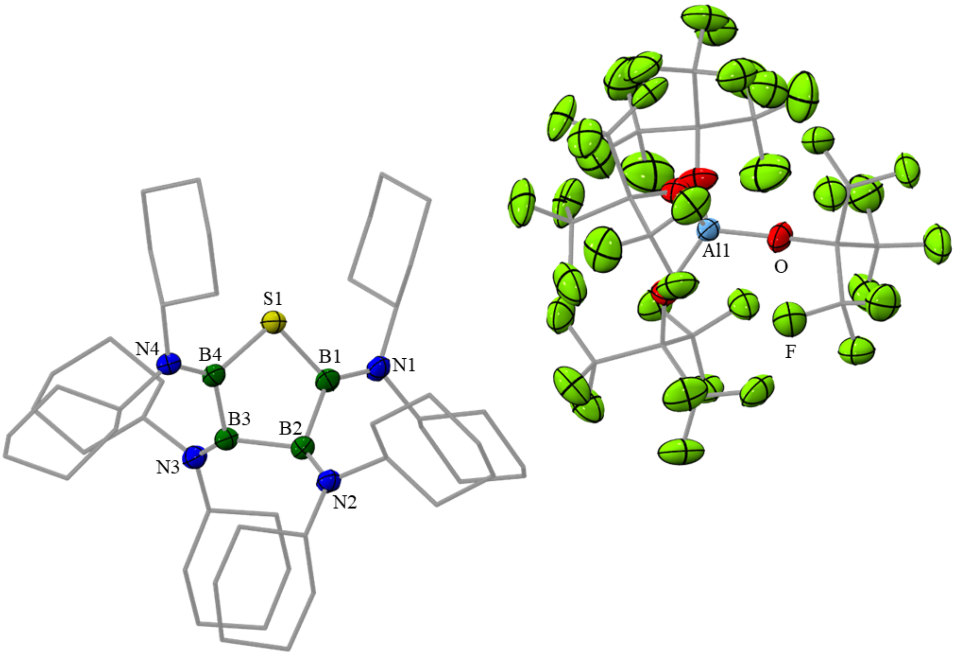

Supplementary Fig. S50 | Solid-state structure of [**2S**][Al(OC(CF$_3$)$_3$)$_4$]. Atomic displacement ellipsoids represented at 50%. Ellipsoids of the ligand periphery and hydrogen atoms have been omitted for clarity.

Refinement details for 4S: One cyclohexyl group is disordered. The atomic displacement parameters of atoms of the disordered cyclohexyl group C1_4 to C6_11 were restrained with the RIGU keyword in ShelXL ('enhanced rigid bond' restraint for all bonds in the connectivity list). The displacement parameters of atoms of the disordered cyclohexyl group C1_4 to C6_11 were restrained to the same value with the similarity restraint SIMU. One triflate group is disordered. The atomic displacement parameters of atoms C1_14 to O3_15 were restrained using DELU keyword in ShelXL input ('rigid bond' restraint for the bonds in the connectivity list). The atomic displacement parameters of atoms C1_14 to O3_15 were restrained with the RIGU keyword in the ShelXL input ('enhanced rigid bond' restraint for all bonds in the connectivity list). The displacement parameters of atoms C1_14 to O3_15 were restrained to

the same value with the similarity restraint SIMU. The 1–2 and 1–3 distances in the disordered TRIF group were restrained to the same values with SAME. One CF_3 group is disordered. The atomic displacement parameters of atoms C1_12 to F3_13 were restrained with the RIGU keyword in the ShelXL input ('enhanced rigid bond' restraint for all bonds in the connectivity list). The displacement parameters of atoms C1_12 to F3_13 were restrained to the same value with the similarity restraint SIMU. The U_{ii} displacement parameters of atoms of the disordered CF_3 group C1_12 to F3_13 were restrained with the ISOR keyword with esd = 0.015 to approximate isotropic behavior. The unit cell contains solvent molecules (144 electrons found in S.A.V) which have been treated as a diffuse contribution to the overall scattering without specific atom positions by SQUEEZE/PLATON.

Crystal data for **4S**: $C_{50}H_{86}B_4F_6N_4O_6S_3$, M_r = 1092.64, colorless block, 0.430×0.330×0.220 mm^3, monoclinic space group $P2_1/n$, a = 14.5369(2) Å, b = 23.1284(2) Å, c = 18.5376(2) Å, β = 91.4530(10)°, V = 6230.62(12) Å^3, Z = 4, ρ_{calcd} = 1.165 g·cm^{-3}, μ = 1.613 mm^{-1}, F(000) = 2336, T = 100(2) K, R_1 = 0.0710, wR_2 = 0.1758, 12709 independent reflections [2θ≤151.614°] and 879 parameters.

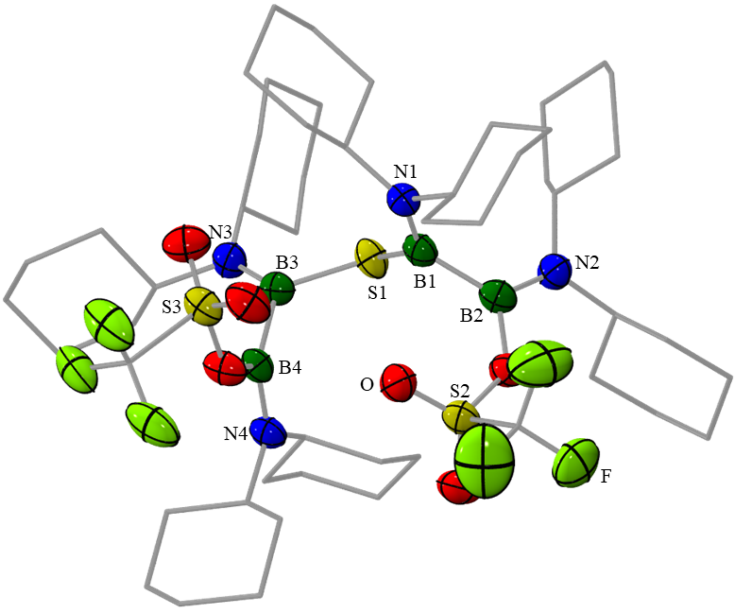

Supplementary Fig. S51 | Solid-state structure of **4S**. Atomic displacement ellipsoids represented at 50%. Ellipsoids of the ligand periphery and hydrogen atoms have been omitted for clarity.

Crystal data for **5F**: $C_{48}H_{88}B_4F_2N_4$, M_r = 802.46, colorless block, 0.160×0.060×0.040 mm^3, monoclinic space group $P2_1/n$, a = 12.7822(2) Å, b = 16.3623(2) Å, c = 24.0141(3) Å, α = 90°,

β = 95.0710(10)°, γ = 90°, V = 5002.80(12) Å^3, Z = 4, ρ_{calcd} = 1.065 g·cm^{-3}, μ = 0.498 mm^{-1}, F(000) = 1768, T = 100(2) K, R_1 = 0.0560, wR_2 = 0.0992, 9492 independent reflections [2θ≤140.148°] and 523 parameters.

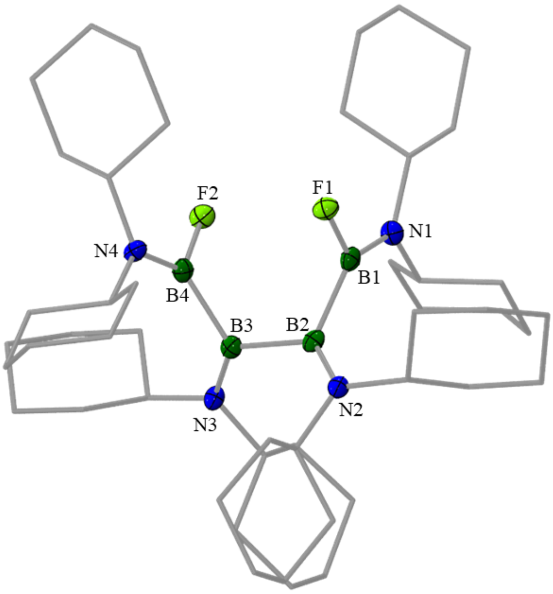

Supplementary Fig. S52 | Solid-state structure of **5F**. Atomic displacement ellipsoids represented at 50%. Ellipsoids of the ligand periphery and hydrogen atoms have been omitted for clarity.

Refinement details for 5Cl: The atomic displacement parameters of atoms C1_11 to C6_111 were restrained with the RIGU keyword in the ShelXL input ('enhanced rigid bond' restraint for all bonds in the connectivity list). The displacement parameters of atoms C1 to C6 of the residues 11 and 111 were restrained to the same value with the similarity restraint SIMU. The U_{ii} displacement parameters of atoms C1_11 to C6_111 were restrained with the ISOR keyword to approximate isotropic behavior.

Crystal data for **5Cl**: $C_{48}H_{88}B_4Cl_2N_4$, M_r = 835.36, colorless block, 0.200×0.120×0.100 mm^3, orthorhombic space group *Pbca*, a = 21.3148(3) Å, b = 22.6255(2) Å, c = 41.1247(5) Å, V = 19832.7(4) Å^3, Z = 16, ρ_{calcd} = 1.119 g·cm^{-3}, μ = 1.429 mm^{-1}, F(000) = 7328, T = 100(2) K, R_1 = 0.0799, wR_2 = 0.1593, 19954 independent reflections [2θ≤150.916°] and 1100 parameters.

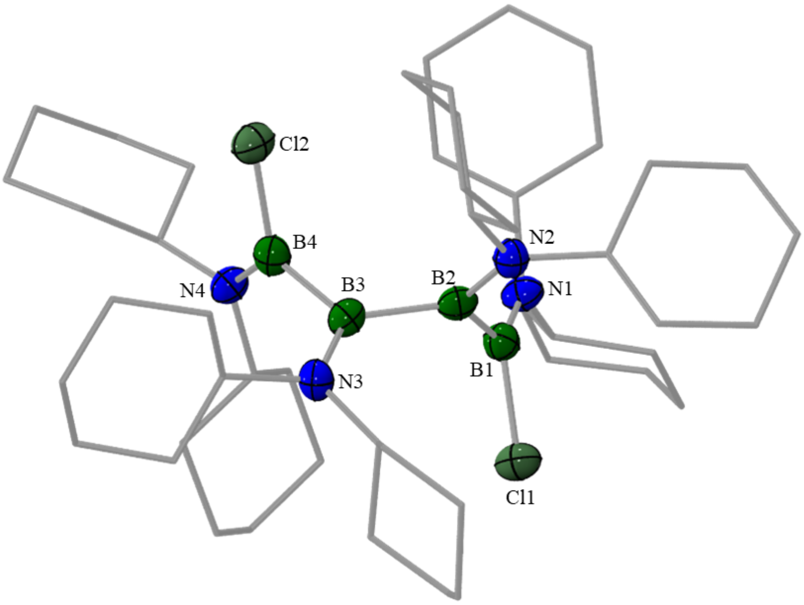

Supplementary Fig. S53 | Solid-state structure of **5Cl**. Atomic displacement ellipsoids represented at 50%. Ellipsoids of the ligand periphery and hydrogen atoms have been omitted for clarity.

Refinement details for 5Br: Two cyclohexyl moieties showed disorder. The atomic displacement parameters (ADPs) of overlapping atoms from different PARTs (C1 to C6 of RESIs 8/18 and 9/19) were restrained using the similarity restraint (SIMU) and the rigid body restraint (RIGU). The 1–2 and 1–3 distances of the disordered cyclohexyl moieties were restrained using the same distance restraint (SAME). The N4_1-C1_8/18 and N4_1-C1_9/19 distances were restrained using the same distance restraint (SADI).

Crystal data for **5Br**: $C_{48}H_{88}B_4Br_2N_4$, M_r = 924.28, colorless block, 0.150×0.130×0.100 mm^3, triclinic space group $P\bar{1}$, a = 11.8121(2) Å, b = 12.5420(2) Å, c = 17.6039(3) Å, α = 81.5890(10)°, β = 77.3600(10)°, γ = 77.7040(10)°, V = 2472.77(7) Å^3, Z = 2, ρ_{calcd} = 1.241 g·cm^{-3}, μ = 2.332 mm^{-1}, F(000) = 988, T = 100(2) K, R_1 = 0.0375, wR_2 = 0.0967, 8984 independent reflections [2θ≤136.474°] and 633 parameters.

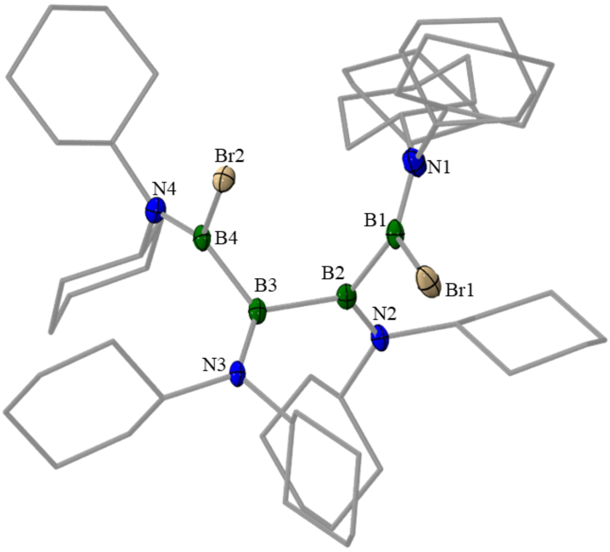

Supplementary Fig. S54 | Solid-state structure of **5Br**. Atomic displacement ellipsoids represented at 50%. Ellipsoids of the ligand periphery and hydrogen atoms have been omitted for clarity.

Crystal data for **6**: $C_{24}H_{44}B_2Br_2N_2$, M_r = 542.05, colorless block, 0.170×0.110×0.100 mm^3, orthorhombic space group *Pbca*, *a* = 15.92470(10) Å, *b* = 17.36570(10) Å, *c* = 19.4179(2) Å, α = 90°, β = 90°, γ = 90°, *V* = 5369.89(7) Å^3, *Z* = 8, ρ_{calcd} = 1.341 g·cm^{-3}, μ = 3.906 mm^{-1}, *F*(000) = 2256, *T* = 100(2) K, R_1 = 0.0240, wR_2 = 0.0623, 4916 independent reflections [2θ≤136.416°] and 271 parameters.

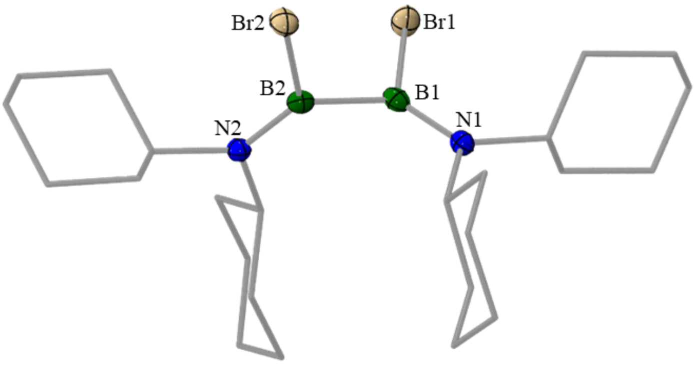

Supplementary Fig. S55 | Solid-state structure of **6**. Atomic displacement ellipsoids represented at 50%. Ellipsoids of the ligand periphery and hydrogen atoms have been omitted for clarity.

Crystal data for **[Cy$_2$NBO]$_3$**: $C_{36}H_{66}B_3N_3O_3$, M_r = 621.34, colorless plate, 0.218×0.148×0.074 mm^3, triclinic space group $P\bar{1}$, a = 11.3580(10) Å, b = 13.6453(11) Å, c = 13.7088(15) Å, α = 60.526(2)°, β = 79.942(4)°, γ = 85.107(3)°, V = 1821.2(3) Å^3, Z = 2, ρ_{calcd} = 1.133 g·cm^{-3}, μ = 0.069 mm^{-1}, $F(000)$ = 684, T = 100(2) K, R_1 = 0.0721, wR^2 = 0.1391, 7196 independent reflections [2θ≤52.248°] and 406 parameters.

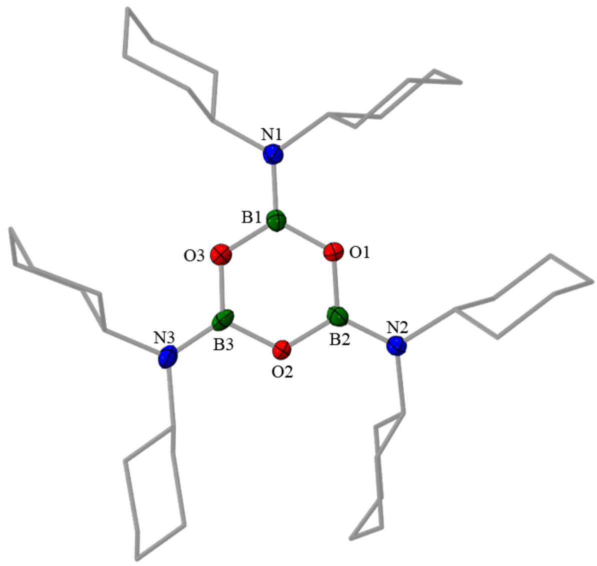

Supplementary Fig. S56 | Solid-state structure of **[Cy$_2$NBO]$_3$**. Atomic displacement ellipsoids represented at 50%. Ellipsoids of the ligand periphery and hydrogen atoms have been omitted for clarity.

Computational details

General. All computations were performed using the Gaussian16 (Revision C.01) package[6]. All structures were fully optimized without symmetry constraints at the ωB97xd level of theory employing def2-SVPP basis sets for all atoms[7,8]. Zero-point vibrational energies and thermal corrections were computed from frequency calculations with a standard state of 298 K and 1 atm; thermal free energies (ΔE_{298}) were obtained from these single-point frequency calculations. The presence of true energy minima on the potential energy surface was verified for all optimized species by the absence of imaginary frequencies. For EPR, NMR, and orbital calculations Def2-TZVP basis sets were used, combined with the SMD solvation model (scrf=smd) for inclusion of tetrahydrofuran solvent effects[9]. Calculated ^{19}F NMR chemical shifts were referenced to CCl_3F, the commonly used external standard in NMR spectroscopy, by first referencing to the calculated chemical shift of optimized C_6F_6 and putting the values into relation to its experimentally observed value of δ = –164.9 ppm. Illustrations of optimized structures, as well as orbital and spin density plots were prepared with IQmol 3.1.3[10]. The four different potential conformers of $B_4(NCy_2)_4F_2$ (**5F**) were generated as follows:

5F^{conf1} – optimization using the coordinates of the “gauche”-type X-ray structure of **5F** as input geometry.

5F^{conf2} – optimization using the coordinates of the “gauche”-type X-ray structure of **5F** and one 180° rotation around the B2−B3 bond as input geometry.

5F^{conf3} – optimization using the coordinates of the “gauche”-type X-ray structure of **5F** and one 180° rotation around the B3−B4 bond as input geometry.

5F^{conf4} – optimization using the coordinates of the “gauche”-type X-ray structure of **5F** and two subsequent 180° rotations around the B2−B3 and B3−B4 bonds as input geometry.

Supplementary Table S1 | Averaged Fermi contact coupling constants calculated at two different levels of theory for the boron and nitrogen centers of **[2S]$^{•+}$** in Hz (SMD(dimethoxyethane)).

	S	B(1)	B(2)	B(3)	B(4)
ωB97xd/cc-pVTZ	-2.4	34.6	-47.5	-26.8	10.9
B3LYP-GD3BJ/cc-pVTZ	-2.1	39.2	-19.3	-6.7	15.0

Supplementary Table S2 | Calculated isotropic ^{19}F NMR chemical shifts δ^{19F}_{calc} of the four potential conformers of $B_4(NCy_2)_4F_2$ (**5F**) in ppm (referenced to CCl_3F as external standard).

	**5F^{conf1}**	**5F^{conf2}**	**5F^{conf3}**	**5F^{conf4}**
δ^{19F}_{calc}	-73.1, -73.1	-76.7, -76.7	-71.8, -72.6	-65.1, -81.8

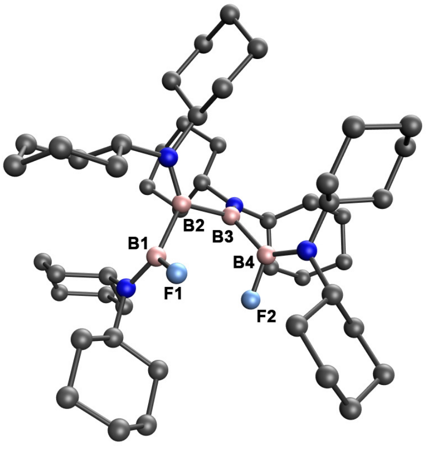

5F^{conf1}

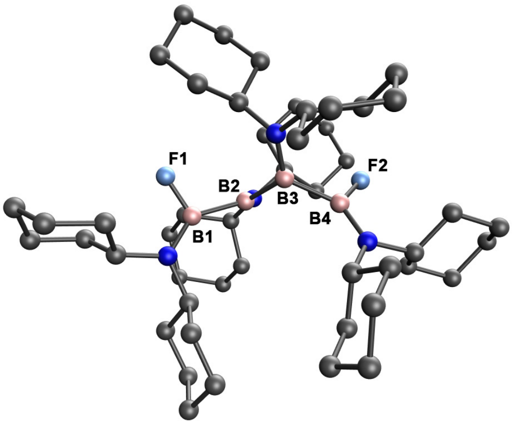

5F^{conf2}

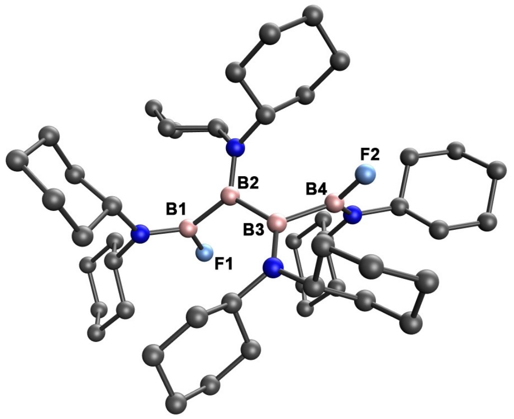

5F^{conf3}

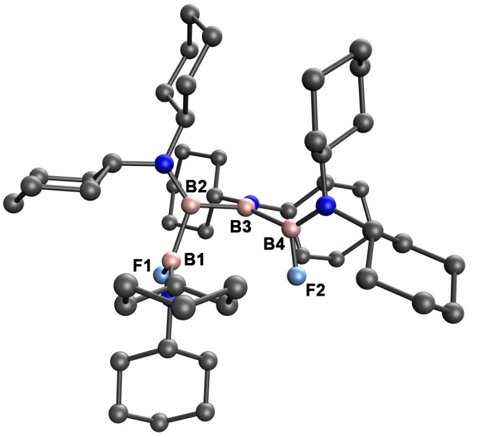

5F^{conf4}

Supplementary Fig. S57 | Optimized structures of the four potential conformers of $B_4(NCy_2)_4F_2$ (**5F**).

Supplementary References

1. Stoll, S. & Schweiger, A. EasySpin, a comprehensive software package for spectral simulation and analysis in EPR. *J. Magn. Reson*. **178**, 42-55 (2006).
2. Beck, E. et al. Structural stability of a bent tetra(amino)tetraborane ring across four charge states. *Chem.* **11**, 102338 (2025).
3. Noviandri, I. et al. The Decamethylferrocenium/Decamethylferrocene Redox Couple: A Superior Redox Standard to the Ferrocenium/Ferrocene Redox Couple for Studying Solvent Effects on the Thermodynamics of Electron Transfer. *J. Phys. Chem. B* **103**, 6713–6722 (1999).
4. Sheldrick, G. SHELXT – Integrated space-group and crystal-structure determination. *Acta Cryst*. **71**, 3–8 (2015).
5. Sheldrick, G. A short history of SHELX. *Acta Cryst*. **64**, 112-122 (2008).
6. Frisch, M. J. et al. Gaussian 16, Revision C.01, Gaussian, Inc.: Wallingford CT (2016).
7. Chai, J.-D. & Head-Gordon, M. Long-range corrected hybrid density functionals with damped atom-atom dispersion corrections. *Phys. Chem. Chem. Phys.* **10**, 6615–6620 (2008).
8. Weigend, F. & Ahlrichs, R. Balanced basis sets of split valence, triple zeta valence and quadruple zeta valence quality for H to Rn: Design and assessment of accuracy. *Phys. Chem. Chem. Phys*. **7**, 3297–3305 (2005).
9. Marenich, A. V., Cramer, C. J. & Truhlar, D. G. Universal Solvation Model Based on Solute Electron Density and on a Continuum Model of the Solvent Defined by the Bulk Dielectric Constant and Atomic Surface Tensions. *J. Phys. Chem. B* **113**, 6378–6396 (2009).
10. Gilbert, A. IQmol (Version v3.1.3), http://www.iqmol.org (2024).
